# Genome-Wide Identification and Characterization of the Aquaporin Gene Family and Transcriptional Responses to Boron Deficiency in *Brassica napus*

**DOI:** 10.3389/fpls.2017.01336

**Published:** 2017-08-02

**Authors:** Dan Yuan, Wei Li, Yingpeng Hua, Graham J. King, Fangsen Xu, Lei Shi

**Affiliations:** ^1^National Key Laboratory of Crop Genetic Improvement, Huazhong Agricultural University Wuhan, China; ^2^Microelement Research Center/Key Laboratory of Arable Land Conservation (Middle and Lower Reaches of Yangtze River), Ministry of Agriculture, Huazhong Agricultural University Wuhan, China; ^3^Southern Cross Plant Science, Southern Cross University Lismore, NSW, Australia

**Keywords:** aquaporins (AQPs), *Brassica napus*, boron (B), transcriptional profile, boron homeostasis

## Abstract

Aquaporins (AQPs) are an abundant protein family and play important roles to facilitate small neutral molecule transport across membranes. Oilseed rape (*Brassica napus* L.) is an important oil crop in China and elsewhere in the world, and is very sensitive to low boron (B) stress. Several AQP family genes have been reported to be involved in B transport across plasma membranes in plants. In this study, a total of 121 full-length AQPs were identified and characterized in *B. napus* (AC genome), and could be classified into four sub-families, including 43 PIPs (plasma membrane intrinsic proteins), 35 TIPs (tonoplast intrinsic proteins), 32 NIPs (NOD26-like intrinsic proteins), and 11 SIPs (small basic intrinsic proteins). The gene characteristics of *BnaAQP*s were similar to those of *BraAQP*s (A genome) and *BolAQP*s (C genome) including the composition of each sub-family, gene structure, and substrate selectivity filters. The *BnaNIP* was the most complex AQP sub-family, reflecting the composition of substrate selectivity filter structures which affect the permeation of solution molecules. In this study, the seedlings of both B-efficient (QY10) and B-inefficient (W10) cultivars were treated with two boron (B) levels: deficient (0.25 μM B) and sufficient (25 μM B). The transcription of AQP genes in root (R), juvenile leaf (JL), and old leaf (OL) tissues of both cultivars was investigated under B deficient and sufficient conditions. Transcription of most *BnaPIP*s and *BnaTIP*s was significantly increased compared with other *BnaAQP*s in all the three tissues, especially in the roots, of both B-efficient and B-inefficient cultivars under both B conditions. With B deprivation, the expression of the majority of the *BnaPIP*s and *BnaTIP*s was down-regulated in the roots. However, the *BnaNIP*s were up-regulated. In addition, the *BnaCnn_random*.*PIP1;4b, BnaPIP2;4*s, *BnaC04.TIP4;1a, BnaAnn_random.TIP1;1b*, and *BnaNIP5;1*s (except for *BnaA07.NIP5;1c* and *BnaC06.NIP5;1c*) exhibited obvious differences at low B between B-efficient and B-inefficient cultivars. These results will help us to understand boron homeostasis in *B. napus*.

## Introduction

Aquaporins (AQPs) are a superfamily of major intrinsic proteins (MIP) that selectively facilitate water and small neutral molecules across biological membranes (Maurel and Chrispeels, 2001). Recently, the AQP genes in the genomes of some plants have been identified, with 35 in *Arabidopsis thaliana* (*A. thaliana*), 60 in *Brassica rapa* (*B. rapa*), 67 in *B. oleracea*, 31 in maize, 33 in rice, 41 in common bean, and 30 in barley (Chaumont and Jung, 2001; Johanson et al., 2001; Sakurai et al., 2005; Tao et al., 2014; Ariani and Gepts, 2015; Diehn et al., 2015; Tombuloglu et al., 2015). Based on sequence similarity and subcellular localization, the AQPs are divided into five distinct sub-families in higher plants which consists of plasma membrane intrinsic proteins (PIPs), tonoplast intrinsic proteins (TIPs), NOD26-like intrinsic proteins (NIPs), small basic intrinsic proteins (SIPs), and uncategorized X intrinsic proteins (XIPs) (Ishikawa et al., 2005; Maurel et al., 2008; Wudick et al., 2009). AQP proteins of different sub-families share a conserved three-dimensional (3D) structure in cell membranes, although they vary in sequence, subcellular localization, and in planta physiological function, especially in substrate selectivity. Each AQP protein contains a six α-helical transmembrane structure (TM1-TM6), five loops (LA-LE), and two additional half-helices (HB, HE) (Daniels et al., 1999; Wallace et al., 2006). There are two selectivity filters within the protein structure that determine substrate specificity. One is composed of two highly conserved NPA motifs located in HB and HE, where HB and HE form a narrow central aqueous pore together, and the other is an ar/R (aromatic/arginine) selectivity filter consisting of four residues, one in transmembrane 2 (TM2), one in transmembrane 5 (TM5), and two in loop E (LE1 and LE2) (Wallace and Roberts, 2006; Hub and Groot, 2008; Mitaniueno et al., 2011).

Boron (B) is an essential micronutrient (Warington, 1923) for plant growth and development. Boron deficiency is a widespread problem for field crop production, causing large losses of crop yield annually both quantitatively (Wei et al., 1998) and qualitatively (Bell and Dell, 2008). *AtNIP5;1* is involved in efficient B uptake in the roots of *A. thaliana* (Brassicaceae) under B deficiency (Takano et al., 2006) and further studies have indicated that the polar localization of NIP5;1 plays an important role in B absorption, which depends on threonine phosphorylation in the conserved three amino acid (ThrProGly, TPG) repeat of the N-terminal region, and is maintained by clathrin-mediated endocytosis (Wang S. et al., 2017). Moreover, the minimum open reading frame (AUG-stop, “AUGTAA”) in 5′UTR of *AtNIP5;1* has been identified as the crucial element in response to cytoplasmic B concentration that regulates gene expression and induces ribosome stalling and mRNA degradation under high B conditions (Tanaka et al., 2016).

The *Brassica* genus diverged from a common ancestor with *Arabidopsis* around 18 MYA (Yang et al., 2006). *B. napus* (A_n_A_n_C_n_C_n_, 2n = 4x = 38) is an important oil crop species in the world, which most likely originated in domestication from the natural spontaneous hybridization between the diploid ancestors *B. oleracea* (C_o_C_o_, 2n = 2x = 18) and *B. rapa* (A_r_A_r_, 2n = 2x = 20) ~7,500 years ago, resulting in chromosome doubling (Chalhoub et al., 2014). In *B. napus*, we have previously identified six orthologous genes of *NIP5;1*, with *BnaA03.NIP5;1b*, and *BnaC02.NIP5;1a* proposed as candidate genes underlying the major-effect B efficiency QTLs *qBEC-A3a* and *qBEC-C2a*, respectively (Hua et al., 2016a,b). *AtNIP6;1* is essential for preferential distribution of B into growing tissues under low B (Tanaka et al., 2008). Yeast complementation assays have confirmed that *HvPIP1;3* and *HvPIP1;4* can facilitate B transport across the membranes into yeast cells (Fitzpatrick and Reid, 2009). However, the overexpression of *AtTIP5;1* and rice *OsPIP2;4, OsPIP2;7, OsPIP1;3*, and *OsPIP2;6* in *Arabidopsis* notably increased its tolerance to high levels of B, and the latter two are involved the influx and efflux transport of B (Pang et al., 2010; Kumar et al., 2014; Mosa et al., 2015). In addition, AQP proteins can also facilitate transport of many other small molecule nutrients, such as Lis1 (OsNIP2;1) for silicon transport (Ma et al., 2006), AtTIP2;1 and AtTIP2;3 for NH_3_ transport (Loqué et al., 2005), AtNIP3;1 for arsenic uptake (Xu et al., 2015), and AtNIP1;2 facilitating Al-malate transport (Wang Y. et al., 2017), respectively.

The ancestral sub-genomes of *Brassica* species may be classified based on gene density and divergence, and have been nominated as LF (the least fractionized sub-genome), MF1 (the moderate gene fractionized sub-genome), and MF2 (the most gene fractionized sub-genome) (Cheng et al., 2012). A reference genome sequence of *B. napus* (cultivar Darmor-*bzh*) has been anchored to all 19 chromosomes (Chalhoub et al., 2014). In this study, we have identified and characterized a complete set of 121 full-length AQPs in the *B. napus* genome, which can be divided into four sub-families based on a phylogenetic tree that also incorporated *A. thaliana* orthologues. Phylogenetic relationships were established, and used as a context for investigating variation in gene structure, chromosomal and sub-genome distribution, and protein structural features of *AQP*s. The transcription of AQP genes in root (R), juvenile leaf (JL), and old leaf (OL) tissues of a B-efficient and a B-inefficient cultivar was also investigated under B deficient and sufficient conditions.

## Materials and methods

### Identification of *BnaAQP* genes in *B. napus*

AQP genes were identified in *B. napus* based on their homology similarity to *Arabidopsis*. The genome sequences and gene IDs of the 35 AQP genes were obtained from the TAIR10 database (http://www.arabidopsis.org/index.jsp) and used to perform a BLAST analysis and search for the AQP homologous genes in the CNS-Genoscope database (http://www.genoscope.cns.fr/brassicanapus/) (Chalhoub et al., 2014) and *Brassica* database (BRAD; http://brassicadb.org/brad/index.php), respectively. The genomic, cDNA, CDS, as well as protein sequences, of *BnaAQP*s were obtained from CNS-Genoscope database with inclusion of each AQP protein determined by checking the candidate genes using the Hidden Markov Model of the Pfam database (http://pfam.sanger.ac.uk/search), SMART database (http://smart.embl-heidelberg.de/), and NCBI Conserved Domain Search database (https://www.ncbi.nlm.nih.gov/Structure/bwrpsb/bwrpsb.cgi). Redundant sequences lacking either NPA motifs or ar/R selectivity filters were removed. Finally, the full-length *BnaAQP* family genes were identified. The *BnaAQP* genes were renamed according to the format: <genus (one letter)> <species (two letters)> <sub-genome (three letters)> <gene name (based on the orthologous gene name with *Arabidopsis*)> <allele (paralogous genes of each sub-genome according to their physical locations from the first chromosome to the next chromosome were named a, b, c, etc.)>, according to the standardized gene nomenclature for *Brassica* genus proposed by Østergaard and King (2008).

### Multiple alignment and phylogenetic analysis of *BnAQP* family genes

Multiple sequence alignment of protein sequences of *B. napus* and *Arabidopsis* was performed using ClustalW tool of MEGA 5.1 (Tamura et al., 2011). An unrooted phylogenetic tree of the 121 full-length AQP protein sequences was then constructed using MEGA 5.1 with the Neighbor Joining (NJ) method, and the analysis of bootstrap values was conducted using 1,000 replicates.

### Chromosome locations and gene structure analysis of *BnaAQP* genes

The chromosome location information of the *BnAQP* genes was obtained from BRAD. The MapInspect software was used to draw the gene chromosome location diagrams (**Figure 2**). The exon-intron structures of the *BnAQP* family genes were determined based on alignments of their coding sequences with the corresponding genomic sequences, and the diagram was drawn using GSDS (Gene structure display server: GSDS; http://gsds.cbi.pku.edu.cn/).

### Conserved motifs and physicochemical parameters analysis of *BnaAQP* proteins

Conserved motifs were identified by the MEME tool (Version 4.11.2) (http://meme-suite.org/tools/meme). The parameter settings were default values apart from: range of optimum width of each motif from 6 to 50 and maximum number of motifs to search: 15. The physicochemical parameters, including molecular weight (MW) and isoelectronic points (pI), of each BnaAQP protein were calculated using the compute pI/MW tool of ExPASy (http://www.expasy.org/tools/). GRAVY (grand average of hydropathy) values were calculated using the PROTPARAM tool (http://web.expasy.org/protparam/). Subcellular localization prediction was conducted using the WoLF PSORT (https://wolfpsort.hgc.jp/) server.

### Syntenic gene analysis between *A. thaliana* and *B. napus*

Syntenic genes between *A. thaliana* and *B. napus* were searched by the online tool “syntenic gene analysis” (http://brassicadb.org/brad/searchSyntenytPCK.php) and each syntenic At-Bn ortholog in *B. napus* of AQP were obtained.

### Transcriptional profile of *BnAQP* family genes

The *B. napus* seedlings of the B-efficient cultivar Qingyou 10 (QY10) and the B-inefficient cultivar Westar10 (W10) were planted in Hoagland solution with 0.25 μM B (low B) and 25 μM B (high B) in an illuminated growth room (300–320 μmol m^−2^ s^−1^; 24°C daytime/22°C night; 16 h light/8 h dark) for 30 d. The roots (R), old leaves (OL, without petiole), and juvenile leaves (JL, without petiole) of QY10 and W10 were individually harvested, and each sample included three independent biological replicates. The total RNA of each sample was extracted using RNA extraction kit (BioTeke, Beijing, China) according to the manufacturer's recommendations. The RNA-seq sequencing libraries were subsequently sequenced using Illumina HiSeq™ 2000 (Illumina Inc., San Diego, CA, USA). All raw data were deposited in the NCBI Sequence Read Archive (Bioproject accession number, PRJNA393069). High-quality clean reads were mapped to the reference Darmor-*bzh* genome co-ordinates, and the RPKM (reads per kilo bases per million reads) measure was used to obtain the gene annotation and expression data. The RPKM values of genes were transformed to logarithmic values, which were used to produce a heat map using the program Multiviewer (MeV) (Saeed et al., 2003).

## Results

### Genome-wide identification and phylogenetic analysis of *BnaAQP* gene family in *B. napus*

The sequences and IDs of 35 *AtAQP* genes were used to carry out BLAST alignment against the *B. napus* reference genome, with annotation search used to identify 121 full-length *BnaAQP* genes as well as 15 non-full-length *BnaAQP* genes (Supplementary Table 1). The latter set lacked the transmembrane domains, resulting in the loss of the NPA motif and/or ar/R selectivity filter (Supplementary Table 1).

**Figure 1:**
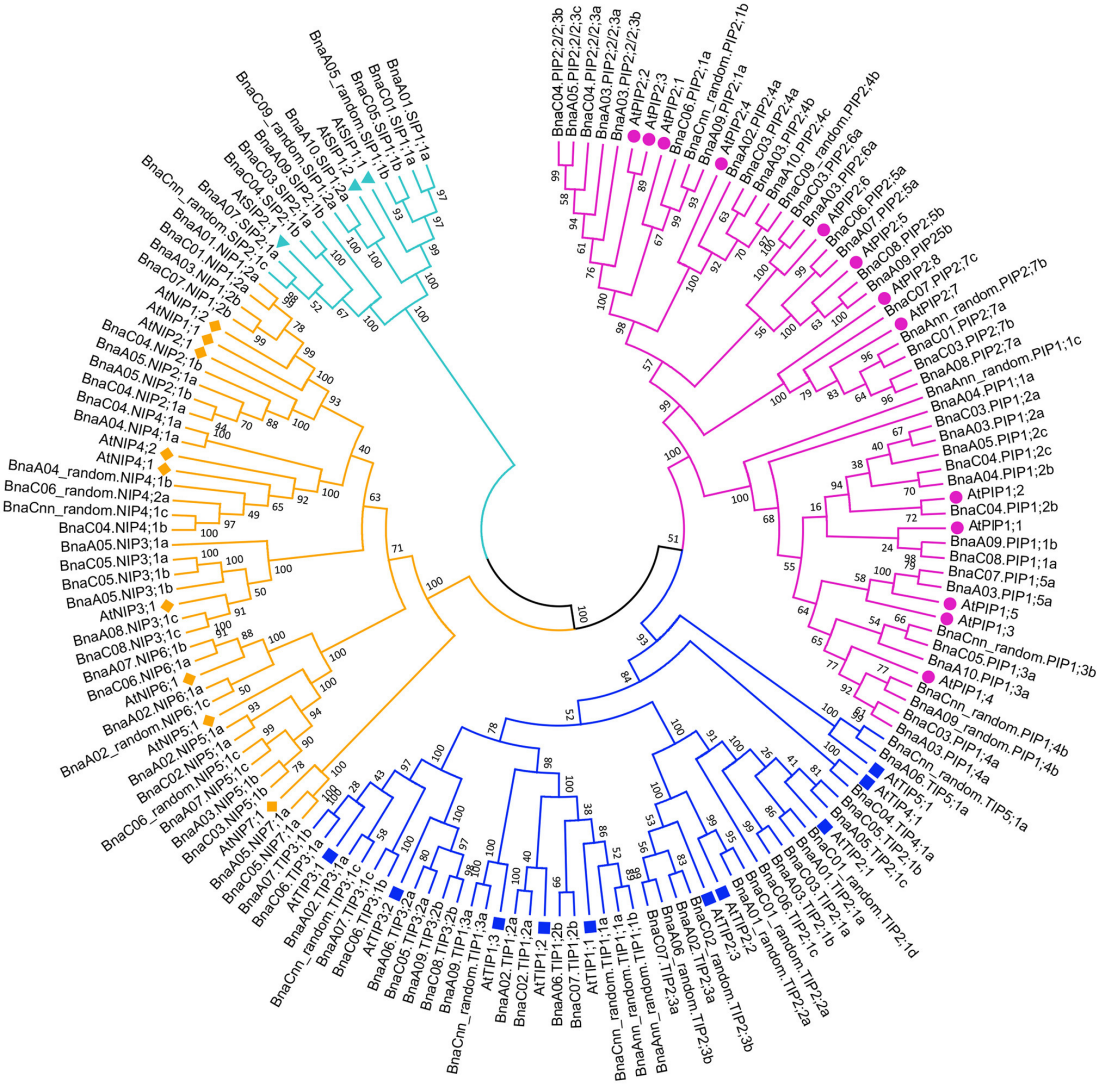
Phylogenetic tree of AQPs in *Brassica* crops. The AQPs phylogenetic tree was generated by MEGA 5.1 with Neighbor Joining (NJ) method and 1,000 replicates bootstraps, based on the amino acid sequences of 121 *BnaAQP* genes and 35 *AtAQP* genes. The gene name with purple, blue, yellow and cyan represented *PIPs, TIPs, NIPs*, and *SIPs*, respectively. AtAQPs, AtPIPs, AtTIPs, AtNIPs, and AtSIPs were marked with circle, square, diamond and triangle, respectively.

A phylogenetic tree was constructed based on the protein sequences of the 121 *BnaAQP* genes together with the 35 *AtAQP* genes (Figure 1). Four subgroups of *BnaAQP*s were readily identified, and coincided with the distribution of *AtAQP*s. All *AtAQP* genes had 1–7 corresponding orthologous genes in *B. napus* except from *AtPIP2;8* and *AtNIP1;1*. Moreover, there were no XIP sub-family genes in either *B. napus* or *A. thaliana*. The *B. napus* sub-families included 43 PIPs, 35 TIPs, 32 NIPs, and 11 SIP members. The PIP sub-family had two sub-groups, 19 members in PIP1 and 24 in PIP2. The TIP sub-family was divided into five sub-groups (TIP1 to TIP5), nine members in TIP1, 13 in TIP2, 10 in TIP3, one in TIP4 and two in TIP5, respectively. SIPs were divided into the SIP1 (6 members) and SIP2 (5 members) sub-groups. The NIPs could be divided into seven sub-groups (NIP1 to NIP7), four members in NIP1, 2 and 6 sub-groups, six in NIP3, 4 and 5 sub-groups and two in NIP7 sub-group. Most (118 members) of the BnaAQPs, were clearly distinguished from each other and within the different sub-families and even different sub-groups of each sub-family. However, the *BnaNIP4;1* and one *BnaNIP4;2* appeared to have conserved amino acid sequences, distinguished not well each other. The orthologous genes of *AtPIP1*s, *BnaA04.PIP1;1a*, and *BnaAnn_random.PIP1;1c* appear to have undergone greater divergence from *PIP1;2*, although *BnaA09.PIP1;1b* and *BnaC08.PIP1;1a*, annotated as *PIP1;2*, were very close to *PIP1;1* in the neighbor joining tree (Figure 1).

### The physicochemical parameters of BnaAQPs

The length of BnaAQPs ranged from 206 aa (*BnaAnn_random.PIP1;1c*) to 323 aa (*BnaC08.NIP3;1c*), with *BnaNIP*s longer than the other *BnaAQP*s. The molecular weight (MW) ranged from 21.31 kDa (*BnaA02.TIP2;3a*) to 34.59 kDa (*BnaA02_random.NIP6;1c*), which was related to the protein length. All the BnaTIP proteins had a low pI (isoelectric point, pI < 7), except for BnaTIP5;1s. The majority of BnaPIPs and BnaNIPs, and all BnaSIPs proteins had pI > 7, while BnaPIP2;1s, BnaPIP2;2/2;3s, BnaPIP2;4s BnaC08.NIP3;1c, BnaC05.NIP7;1a, and BnaA05.NIP7;1a had a relatively low pI (pI < 7). The hydropathy value of all the amino acids was divided by the protein length, which was defined as grand average of hydropathicity (GRAVY). The GRAVY of all BnaAQPs were positive (ranging from 0.21 to 1.07; Table 1), indicating that all they were hydrophobic. Most proteins of each sub-family of BnaAQPs shared similar parameters, while different sub-families of BnaAQPs were distinguished from each other in the values of MW and pI (Table 1 and Supplementary Figure 1).

**Table 1:** Gene sequence characteristics of 121 *BnaAQP*s and their protein physicochemical parameters and putative subcellular localization.

**At isoform**	**Gene ID**	***B. napus* ID**	**AQP name**	**Location**	**Gene length (bp)**	**Protein length (aa)**	**Molecular weight (kDa)**	**GRAVY**	**pI**	**Subcellular localization (Wolf Psort)**
AtPIP1;1	At3g61430	BnaA09g39170D	*BnaA09.PIP1;1b*	27787216..27788864	1,649	286	30.64	0.36	8.86	plas
		BnaC08g31360D	*BnaC08.PIP1;1a*	30756965..30758657	1,693	286	30.63	0.37	8.86	plas
		BnaA04g00710D	*BnaA04.PIP1;1a*	521567..523135	1,569	225	23.81	0.51	9.48	plas
		BnaAnng23190D	*BnaAnn_random.PIP1;1c*	26244087..26247766	3,680	206	21.75	0.63	9.54	vacu
AtPIP1;2	At2g45960	BnaC04g04640D	*BnaC04.PIP1;2b*	3421181..3422815	1,635	286	30.58	0.40	9.16	plas
		BnaC04g50590D	*BnaC04.PIP1;2c*	48172075..48173895	1,821	286	30.53	0.42	9.16	plas
		BnaC03g25510D	*BnaC03.PIP1;2a*	14337490..14339302	1,813	286	30.53	0.42	9.28	plas
		BnaA05g05230D	*BnaA05.PIP1;2c*	2731629..2733344	1,716	286	30.54	0.41	9.16	plas
		BnaA04g26560D	*BnaA04.PIP1;2b*	18805106..18806845	1,740	286	30.53	0.42	9.16	plas
		BnaA03g21210D	*BnaA03.PIP1;2a*	10085704..10087242	1,539	286	30.52	0.42	9.16	plas
AtPIP1;3	At1g01620	BnaA10g00360D	*BnaA10.PIP1;3a*	183464..184716	1,253	286	30.54	0.40	9.03	plas
		BnaCnng08780D	*BnaCnn_random.PIP1;3b*	8157703..8159122	1,420	286	30.62	0.37	8.87	plas
		BnaC05g00440D	*BnaC05.PIP1;3a*	205982..207601	1,620	286	30.62	0.38	9.03	plas
AtPIP1;4	At4g00430	BnaCnng02360D	*BnaCnn_random.PIP1;4b*	2096149..2097741	1,593	286	30.56	0.39	9.02	plas
		BnaC03g32130D	*BnaC03.PIP1;4a*	19779032..19780668	1,637	288	30.73	0.38	9.14	plas
		BnaA09g51960D	*BnaA09_random.PIP1;4b*	166203..167748	1,546	286	30.56	0.39	9.02	plas
		BnaA03g27130D	*BnaA03.PIP1;4a*	13404120..13406027	1,908	288	30.75	0.38	9.14	plas
AtPIP1;5	At4g23400	BnaC07g38190D	*BnaC07.PIP1;5a*	39580905..39582345	1,441	287	30.52	0.41	9.13	plas
		BnaA03g45950D	*BnaA03.PIP1;5a*	23444590..23446032	1,443	287	30.72	0.36	9.13	plas
AtPIP2;1	At3g53420	BnaCnng31040D	*BnaCnn_random.PIP2;1b*	29430225..29432231	2,007	287	30.46	0.55	6.95	plas
		BnaC06g14590D	*BnaC06.PIP2;1a*	17363649..17365803	2,155	287	30.42	0.58	6.50	plas
		BnaA09g33720D	*BnaA09.PIP2;1a*	24799217..24801162	1,946	287	30.40	0.57	7.68	plas
AtPIP2;2	At2g37170	BnaC04g08100D	*BnaC04.PIP2;2/2;3b*	6071014..6072513	1,500	285	30.30	0.51	6.51	plas
AtPIP2;3	At2g37180	BnaA03g17020D	*BnaA03.PIP2;2/2;3a*	7983649..7985012	1,364	283	30.03	0.52	6.51	plas
		BnaC04g08090D	*BnaC04.PIP2;2/2;3a*	6066781..6068303	1,523	285	30.29	0.52	6.95	plas
		BnaA05g07300D	*BnaA05.PIP2;2/2;3c*	3932305..3933770	1,466	310	33.04	0.43	7.68	plas
		BnaA03g17030D	*BnaA03.PIP2;2/2;3b*	7987203..7988660	1,458	285	30.23	0.55	6.95	plas
AtPIP2;4	At5g60660	BnaC03g11160D	*BnaC03.PIP2;4a*	5450719..5452378	1,660	260	27.41	0.40	7.64	plas
		BnaA10g13480D	*BnaA10.PIP2;4c*	10816157..10817535	1,379	260	27.33	0.41	6.88	plas
		BnaA03g08820D	*BnaA03.PIP2;4b*	4009487..4011031	1,545	260	27.36	0.40	7.63	plas
		BnaA02g06180D	*BnaA02.PIP2;4a*	2963246..2964676	1,431	259	27.43	0.35	8.24	plas
		BnaC09g53920D	*BnaC09_random.PIP2;4b*	3860181..3861553	1,373	259	27.16	0.42	6.88	plas
AtPIP2;5	At3g54820	BnaC08g25570D	*BnaC08.PIP2;5b*	27272691..27276118	3,428	286	30.50	0.48	8.99	plas
		BnaC06g15450D	*BnaC06.PIP2;5a*	18158560..18161837	3,278	286	30.62	0.48	8.82	plas
		BnaA09g34600D	*BnaA09.PIP2;5b*	25339104..25341830	2,727	286	30.47	0.48	8.99	plas
		BnaA07g16510D	*BnaA07.PIP2;5a*	13969366..13971846	2,481	286	30.57	0.48	8.83	plas
AtPIP2;6	At2g39010	BnaC03g21800D	*BnaC03.PIP2;6a*	11843142..11847247	4,106	288	30.91	0.47	8.60	plas
		BnaA03g18300D	*BnaA03.PIP2;6a*	8586178..8589413	3,236	288	30.87	0.48	8.60	plas
AtPIP2;7	At4g35100	BnaAnng11630D	*BnaAnn_random.PIP2;7b*	12562296..12563908	1,613	281	29.74	0.49	8.62	plas
		BnaC07g45370D	*BnaC07.PIP2;7c*	43497800..43499645	1,846	254	26.99	0.56	9.49	plas
		BnaC03g65520D	*BnaC03.PIP2;7b*	55224339..55226112	1,774	281	29.77	0.48	8.99	plas
		BnaC01g03410D	*BnaC01.PIP2;7a*	1759948..1761661	1,714	281	29.77	0.48	8.62	plas
		BnaA08g10860D	*BnaA08.PIP2;7a*	10034293..10035945	1,653	281	29.81	0.48	8.99	plas
AtPIP2;8	At2g16850	–	–	–	–	–	–	–	–	
AtTIP1;1	At2g36830	BnaCnng24720D	*BnaCnn_random.TIP1;1a*	23142537..23144230	1,694	221	22.40	0.91	5.65	vacu
		BnaAnng24130D	*BnaAnn_random.TIP1;1b*	27805915..27807206	1,292	221	22.42	0.92	6.00	vacu
		BnaAnng22640D	*BnaAnn_random.TIP1;1a*	25434515..25435770	1,256	221	22.42	0.92	6.00	vacu
AtTIP1;2	At3g26520	BnaC07g23630D	*BnaC07.TIP1;2b*	30124584..30126080	1,497	253	25.76	0.83	5.87	plas
		BnaC02g36210D	*BnaC02.TIP1;2a*	39332961..39334720	1,760	253	25.79	0.83	5.32	plas
		BnaA06g32840D	*BnaA06.TIP1;2b*	21782637..21784173	1,537	253	25.83	0.82	5.61	plas
		BnaA02g28130D	*BnaA02.TIP1;2a*	20756562..20758284	1,723	253	25.79	0.83	5.32	plas
AtTIP1;3	At4g01470	BnaCnng01570D	*BnaCnn_random.TIP1;3a*	1673039..1673797	759	252	25.86	0.81	5.35	plas
		BnaA09g51590D	*BnaA09.TIP1;3a*	5612..6567	956	252	25.86	0.81	5.35	plas
AtTIP2;1	At3g16240	BnaC01g44580D	*BnaC01_random.TIP2;1d*	4074152..4075768	1,617	249	24.98	0.99	5.32	plas
		BnaC06g05270D	*BnaC06.TIP2;1c*	5903845..5905027	1,183	253	25.62	0.92	5.57	vacu
		BnaC05g37160D	*BnaC05.TIP2;1b*	36274289..36275936	1,648	248	24.88	0.97	5.32	plas
		BnaC03g39560D	*BnaC03.TIP2;1a*	24548289..24549868	1,580	249	24.96	0.99	5.30	vacu
		BnaA05g23460D	*BnaA05.TIP2;1c*	17750143..17751914	1,772	248	24.87	0.97	5.30	plas
		BnaA03g34110D	*BnaA03.TIP2;1b*	16580647..16582185	1,539	249	24.94	0.99	5.30	vacu
		BnaA01g28120D	*BnaA01.TIP2;1a*	19628916..19630477	1,562	249	24.99	1.00	5.32	plas
AtTIP2;2	At4g17340	BnaC01g41690D	*BnaC01_random.TIP2;2a*	867957..869098	1,142	250	25.05	1.07	5.10	vacu
		BnaA01g35340D	*BnaA01_random.TIP2;2a*	556242..557354	1,113	250	24.99	1.03	5.10	vacu
AtTIP2;3	At5g47450	BnaC02g46870D	*BnaC02_random.TIP2;3b*	2737513..2738954	1,442	250	25.20	1.00	4.68	vacu
		BnaA06g40020D	*BnaA06_random.TIP2;3b*	1850537..1852013	1,477	251	25.25	1.04	4.98	vacu
		BnaC07g20220D	*BnaC07.TIP2;3a*	26918624..26920233	1,610	251	25.22	1.03	4.98	vacu
		BnaA02g25440D	*BnaA02.TIP2;3a*	18713936..18723357	9,422	210	21.31	0.93	4.76	vacu
AtTIP3;1	At1g73190	BnaCnng50290D	*BnaCnn_random.TIP3;1c*	49836532..49837529	998	266	28.03	0.56	6.70	plas
		BnaC06g34100D	*BnaC06.TIP3;1b*	33719713..33720873	1,161	265	27.94	0.67	6.34	plas
		BnaC06g23750D	*BnaC06.TIP3;1a*	25508189..25509355	1,167	265	27.85	0.64	6.74	plas
		BnaA07g30640D	*BnaA07.TIP3;1c*	21648457..21649601	1,145	265	27.98	0.65	6.54	plas
		BnaA07g22790D	*BnaA07.TIP3;1b*	17293720..17295068	1,349	265	27.85	0.64	6.74	plas
		BnaA02g16380D	*BnaA02.TIP3;1a*	9732696..9733695	1,000	266	28.00	0.56	6.75	plas
AtTIP3;2	At1g17810	BnaC08g37510D	*BnaC08.TIP3;2b*	34263521..34264931	1,410	267	28.50	0.57	6.54	plas
		BnaC05g13770D	*BnaC05.TIP3;2a*	7958454..7959869	1,415	267	28.63	0.54	6.29	plas
		BnaA09g44820D	*BnaA09.TIP3;2b*	30722833..30724255	1,422	267	28.46	0.58	6.54	plas
		BnaA06g12030D	*BnaA06.TIP3;2a*	6242467..6243735	1,268	267	28.55	0.58	6.28	plas
AtTIP4;1	At2g25810	BnaC04g38040D	*BnaC04.TIP4;1a*	39380971..39382403	1,433	249	26.12	0.72	5.31	vacu
AtTIP5;1	At3g47440	BnaCnng15220D	*BnaCnn_random.TIP5;1a*	14194669..14195620	952	258	26.76	0.74	8.46	chlo
		BnaA06g17390D	*BnaA06.TIP5;1a*	9904490..9905449	960	258	26.82	0.71	8.46	chlo
AtSIP1;1	At3g04090	BnaA05g37440D	*BnaA05_random.SIP1;1b*	2999291..3002087	2,797	239	25.58	0.70	9.52	plas
		BnaC05g47470D	*BnaC05.SIP1;1b*	42486578..42489195	2,618	239	25.60	0.68	9.61	plas
		BnaC01g40230D	*BnaC01.SIP1;1a*	38537351..38539338	1,988	256	27.42	0.50	9.93	plas
		BnaA01g33700D	*BnaA01.SIP1;1a*	22789757..22791692	1,936	254	27.18	0.50	9.93	plas
AtSIP1;2	At5g18290	BnaC09g54320D	*BnaC09_random.SIP1;2a*	4140963..4142787	1,825	243	26.21	0.74	10.13	vacu
		BnaA10g16540D	*BnaA10.SIP1;2a*	12543287..12545452	2,166	243	26.21	0.72	10.24	vacu
AtSIP2;1	At3g56950	BnaCnng20470D	*BnaCnn_random.SIP2;1c*	19195494..19196978	1,485	238	26.01	0.66	9.61	vacu
		BnaC04g24760D	*BnaC04.SIP2;1b*	25681472..25683276	1,805	237	25.92	0.67	9.52	vacu
		BnaC03g54990D	*BnaC03.SIP2;1a*	43494567..43496038	1,472	237	25.92	0.67	9.52	vacu
		BnaA09g36250D	*BnaA09.SIP2;1b*	26276941..26277807	867	234	25.57	0.65	9.33	plas
		BnaA07g17050D	*BnaA07.SIP2;1a*	14351012..14352500	1,489	238	26.03	0.67	9.61	vacu
AtNIP1;1	At4g19030	–	–	–	–	–	–	–	–	
AtNIP1;2	At4g18910	BnaC07g35550D	*BnaC07.NIP1;2b*	37948510..37951356	2,847	298	31.66	0.42	8.83	plas
		BnaC01g11410D	*BnaC01.NIP1;2a*	7070500..7072801	2,302	297	31.67	0.42	8.64	plas
		BnaA03g43810D	*BnaA03.NIP1;2b*	22064126..22066329	2,204	298	31.67	0.42	8.61	plas
		BnaA01g09720D	*BnaA01.NIP1;2a*	4783530..4785823	2,294	297	31.56	0.44	7.77	plas
AtNIP2;1	At2g34390	BnaC04g10830D	*BnaC04.NIP2;1b*	8417418..8418594	1,177	286	30.32	0.59	8.95	vacu
		BnaC04g10820D	*BnaC04.NIP2;1a*	8407741..8409070	1,330	286	30.28	0.57	8.57	vacu
		BnaA05g09470D	*BnaA05.NIP2;1b*	5224603..5225713	1,111	286	30.35	0.58	8.95	vacu
		BnaA05g09450D	*BnaA05.NIP2;1a*	5219029..5220548	1,520	286	30.25	0.62	8.25	vacu
AtNIP3;1	At1g31885	BnaC08g07910D	*BnaC08.NIP3;1c*	11521227..11523172	1,946	323	34.59	0.29	6.81	vacu
		BnaC05g29150D	*BnaC05.NIP3;1b*	27895812..27897456	1,645	315	33.76	0.21	8.04	vacu
		BnaC05g29140D	*BnaC05.NIP3;1a*	27854782..27857035	2,254	315	33.76	0.21	8.04	vacu
		BnaA08g07040D	*BnaA08.NIP3;1c*	7086971..7088975	2,005	296	32.10	0.33	9.10	plas
		BnaA05g16550D	*BnaA05.NIP3;1b*	11238953..11240718	1,766	313	33.56	0.24	8.70	vacu
		BnaA05g16540D	*BnaA05.NIP3;1a*	11204268..11206255	1,988	313	33.45	0.37	8.66	vacu
AtNIP4;1	At5g37810	BnaA04g08310D	*BnaA04.NIP4;1a*	7408307..7409766	1,460	283	30.24	0.59	8.24	plas
		BnaC04g34450D	*BnaC04.NIP4;1b*	36058392..36059641	1,250	283	30.03	0.70	8.20	plas
		BnaC04g30520D	*BnaC04.NIP4;1a*	32384514..32385975	1,462	283	30.21	0.58	8.24	plas
		BnaA04g27980D	*BnaA04_random.NIP4;1b*	502473..504538	2,066	228	24.39	0.84	9.51	vacu
		BnaCnng65250D	*BnaCnn_random.NIP4;1c*	64941790..64943809	2,020	267	29.27	0.26	8.43	plas
AtNIP4;2	At5g37820	BnaC06g42210D	*BnaC06_random.NIP4;2a*	1577357..1580899	3,543	283	30.34	0.71	8.81	plas
AtNIP5;1	At4g10380	BnaC03g28980D	*BnaC03.NIP5;1b*	17150140..17157003	6,864	301	31.12	0.52	8.66	plas
		BnaC02g29210D	*BnaC02.NIP5;1a*	28887548..28890855	3,308	301	31.09	0.54	8.90	plas
		BnaA07g16310D	*BnaA07.NIP5;1c*	13840326..13847129	6,804	301	31.28	0.46	9.08	plas
		BnaA03g24370D	*BnaA03.NIP5;1b*	11714083..11722792	8,710	301	31.21	0.53	8.90	plas
		BnaA02g22030D	*BnaA02.NIP5;1a*	14463023..14466088	3,066	301	31.14	0.54	8.66	plas
		BnaC06g42490D	*BnaC06_random.NIP5;1c*	2013292..2036203	22,912	301	31.30	0.45	9.08	plas
		BnaC06g42500D								
AtNIP6;1	At1g80760	BnaA02g36290D	*BnaA02_random.NIP6;1c*	828760..830397	1,638	305	31.98	0.40	8.58	plas
		BnaC06g40240D	*BnaC06.NIP6;1a*	36954103..36955760	1,658	305	31.83	0.43	8.57	plas
		BnaA07g35330D	*BnaA07.NIP6;1b*	23794278..23795974	1,697	305	31.80	0.43	8.57	plas
		BnaA02g19440D	*BnaA02.NIP6;1a*	12064289..12066003	1,715	305	31.86	0.42	8.26	plas
AtNIP7;1	At3g06100	BnaC05g45720D	*BnaC05.NIP7;1a*	41539679..41541271	1,593	272	28.61	0.70	6.57	plas
		BnaA05g31180D	*BnaA05.NIP7;1a*	21548949..21550723	1,775	272	28.60	0.70	6.57	plas

*Plas, Plasma membrane; Vacu, tonoplast membrane; Chlo, chloroplast thylakoid membrane*.

The main types of subcellular localization for all 121 BnaAQPs were predicted to be plasma membrane (plas) and tonoplast membrane (vacu) (Table 1). 43% of TIPs were localized in the tonoplast membrane and almost all the BnaPIPs were localized in the plasma membrane. BnaNIPs were localized in plasma membrane except for BnaNIP2;1s and BnaNIP3;1s (tonoplast membrane). The members of BnaSIPs were equally localized in the plasma membrane and the tonoplast membrane.

### Chromosomal locations of *BnaAQP* genes and syntenic block analysis between *A. thaliana* and *B. napus*

**Figure 2:**
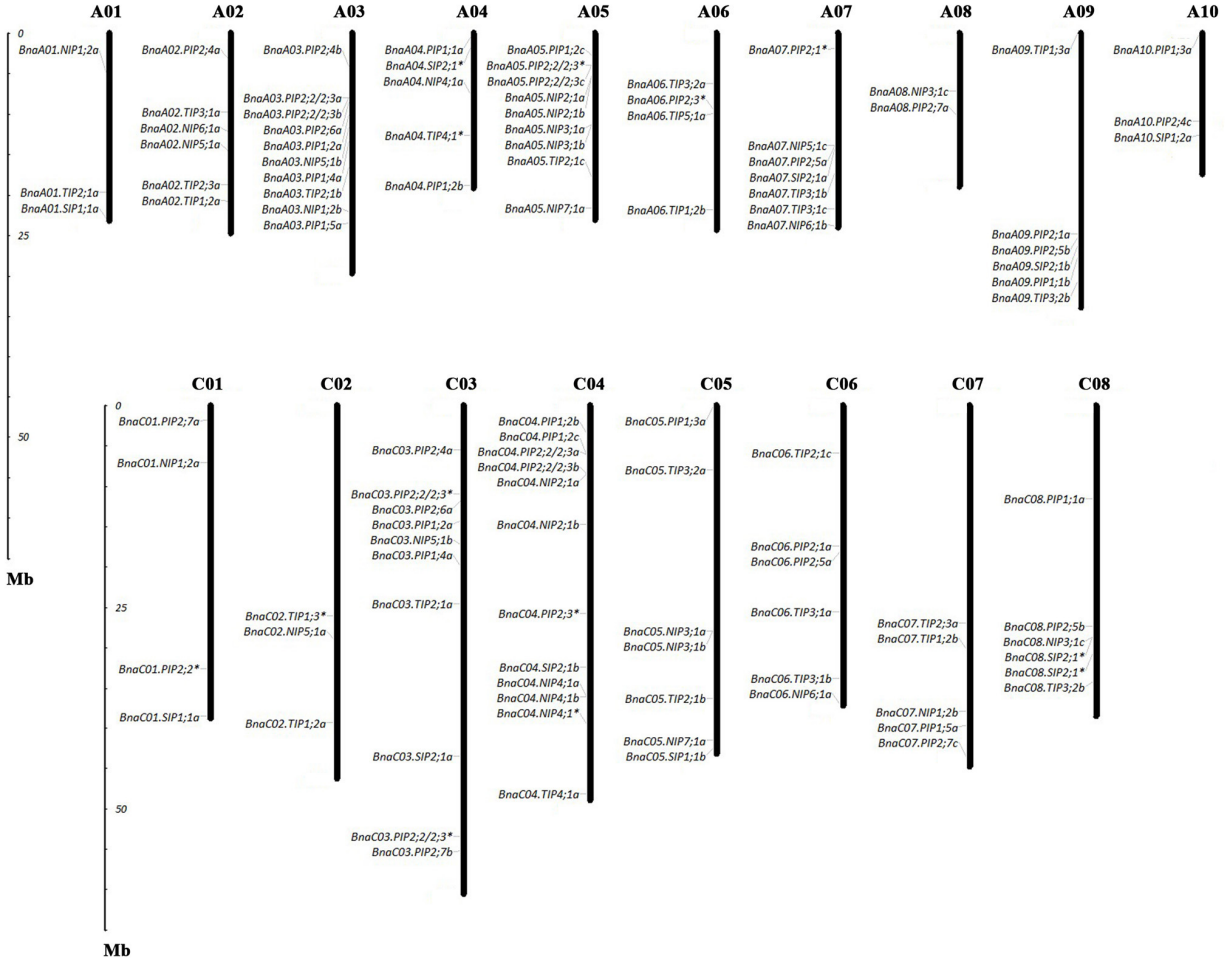
Chromosomal location of AQPs in *Brassica napus*. The gene chromosome location diagram was drawn using the MapInspect software. There were 95 full-length and 13 incomplete *BnaAQP*s located on 18 chromosomes. The genes marked with asterisk (*) represented the incomplete *BnaAQP*s.

Of the genes annotated in this paper as *BnaAQP*s, 95 full-length and 13 non-full-length had previously been incorporated in the genome scaffolds corresponding to 18 of the 19 psedochromosomes of *B. napus* (Figure 2), with the remainder located on scaffolds not yet anchored to a chromosome (Supplementary Table 2). The number of *BnaAQPs* located on each chromosome varied dramatically, with 12 on C04, two on A08 and none on C09. Genes within each *BnaAQP* subfamily were distributed unevenly on the chromosomes. Some clustering was observed at the top of chromosomes A05 and C04, the middle of A03 and C03, and the bottom of chromosome A07 and A09. There was an even distribution on each of the A (55) and C (53) genomes (Figure 2), with 76 full length *BnaAQP*s mapped to 17 syntenic blocks and three non-full-length *BnaAQP*s to two syntenic blocks (Table 2). Thirty three genes were located within the LF sub-genome with the relative proportion of 76 and 55% in MF1 (25) and MF2 (18), respectively, consistent with the total number of paralogues retained in these sub-genomes of both diploid ancestors (Liu et al., 2014).

**Table 2:** Synteny block and distribution of aquaporin genes in the three sub-genomes of *Brassica napus*.

**At isoform**	**Gene ID**	**Synteny block**	**Subgenomes**		
			**LF**	**MF1**	**MF2**
AAtPIP1;1	At3g61430	N	*BnaA09.PIP1;1b*	*BnaA04.PIP1;1a*	–
			*BnaC08.PIP1;1a*	–	–
AtPIP1;2	At2g45960	J	*BnaC04.PIP1;2b*	*BnaC04.PIP1;2c*	–
			*BnaA05.PIP1;2c*	*BnaA04.PIP1;2b*	–
AtPIP1;3	At1g01620	A	*BnaA10.PIP1;3a*	–	–
			*BnaC05.PIP1;3a*	–	–
AtPIP1;4	At4g00430	O	–	*BnaC03.PIP1;4a*	–
			–	*BnaA03.PIP1;4a*	–
AtPIP1;5	At4g23400	U	–	*BnaC07.PIP1;5a*	–
			–	*BnaA03.PIP1;5a*	–
AtPIP2;1	At3g53420	N	*BnaA09.PIP2;1a*	–	*BnaC06.PIP2;1a*
AtPIP2;2	At2g37170	J	–	–	–
AtPIP2;3	At2g37180	J	–	–	–
AtPIP2;4	At5g60660	Wb	*BnaA10.PIP2;4c*	*BnaC03.PIP2;4a*	*BnaA02.PIP2;4a*
			–	*BnaA03.PIP2;4b*	–
AtPIP2;5	At3g54820	N	*BnaC08.PIP2;5b*	–	*BnaA07.PIP2;5a*
			*BnaA09.PIP2;5b*	–	–
AtPIP2;6	At2g39010	J	–	–	*BnaC03.PIP2;6a*
			–	–	*BnaA03.PIP2;6a*
AtPIP2;7	At4g35100	U	*BnaC01.PIP2;7a*	*BnaC07.PIP2;7c*	*BnaC03.PIP2;7b*
			–	–	*BnaA08.PIP2;7a*
AtPIP2;8	At2g16850	H	–	–	–
AtTIP1;1	At2g36830	J	–	–	–
AtTIP1;2	At3g26520	L	*BnaC07.TIP1;2b*	*BnaC02.TIP1;2a*	–
			*BnaA06.TIP1;2b*	*BnaA02.TIP1;2a*	–
AtTIP1;3	At4g01470	O	–	–	–
AtTIP2;1	At3g16240	F	*BnaC05.TIP2;1b*	*BnaA01.TIP2;1a*	*BnaC03.TIP2;1a*
			*BnaA05.TIP2;1c*	–	*BnaA03.TIP2;1b*
AtTIP2;2	At4g17340	U	–	–	–
AtTIP2;3	At5g47450	V	*BnaC07.TIP2;3a*	*BnaA02.TIP2;3a*	–
AtTIP3;1	At1g73190	E	*BnaC06.TIP3;1b*	*BnaA02.TIP3;1a*	–
			*BnaA07.TIP3;1c*	–	–
AtTIP3;2	At1g17810	A	*BnaC05.TIP3;2a*	–	*BnaC08.TIP3;2b*
			*BnaA06.TIP3;2a*	–	*BnaA09.TIP3;2b*
AtTIP4;1	At2g25810	I	–	*BnaC04.TIP4;1a*	–
			–	BnaA04.TIP4;1*	–
AtTIP5;1	At3g47440	M	*BnaA06.TIP5;1a*	–	–
AtSIP1;1	At3g04090	F	–	*BnaC05.SIP1;1b*	–
AtSIP1;2	At5g18290	R	*BnaA10.SIP1;2a*	–	–
AtSIP2;1	At3g56950	N	*BnaA09.SIP2;1b*	*BnaC04.SIP2;1b*	*BnaA07.SIP2;1a*
			BnaC08.SIP2;1*	BnaA04.SIP2;1*	–
AtNIP1;1	At4g19030	U	–	–	–
AtNIP1;2	At4g18910	U	*BnaC01.NIP1;2a*	*BnaC07.NIP1;2b*	–
			*BnaA01.NIP1;2a*	*BnaA03.NIP1;2b*	–
AtNIP2;1	At2g34390	J	*BnaC04.NIP2;1b*	–	–
			*BnaC04.NIP2;1a*	–	–
			*BnaA05.NIP2;1b*	–	–
			*BnaA05.NIP2;1a*	–	–
AtNIP3;1	At1g31885	B	–	*BnaC08.NIP3;1c*	*BnaC05.NIP3;1b*
			–	*BnaA08.NIP3;1c*	*BnaC05.NIP3;1a*
			–	–	*BnaA05.NIP3;1b*
			–	–	*BnaA05.NIP3;1a*
AtNIP4;1	At5g37810	S	–	–	–
AtNIP4;2	At5g37820	S	–	–	–
AtNIP5;1	At4g10380	P	–	*BnaC03.NIP5;1b*	*BnaC02.NIP5;1a*
			–	*BnaA03.NIP5;1b*	*BnaA02.NIP5;1a*
AtNIP6;1	At1g80760	E	*BnaC06.NIP6;1a*	*BnaA02.NIP6;1a*	–
			*BnaA07.NIP6;1b*	–	–
AtNIP7;1	At3g06100	F	*BnaC05.NIP7;1a*	–	–
			*BnaA05.NIP7;1a*	–	–

There were 76 full-length AQP genes in 17 syntenic blocks and 3 incomplete genes in 2 syntenic blocks. LF, Least fractionated subgenome; MF1, medium fractionated subgenome; MF2, more fractionated genome. The genes marked with “^*^” represented the incomplete genes. “–” indicated that no synthetic ortholog of AtAQP was found in the corresponding subgenome in Brassica napus.

### Gene structure and conserved domains of *BnaAQP*s

**Figure 3:**
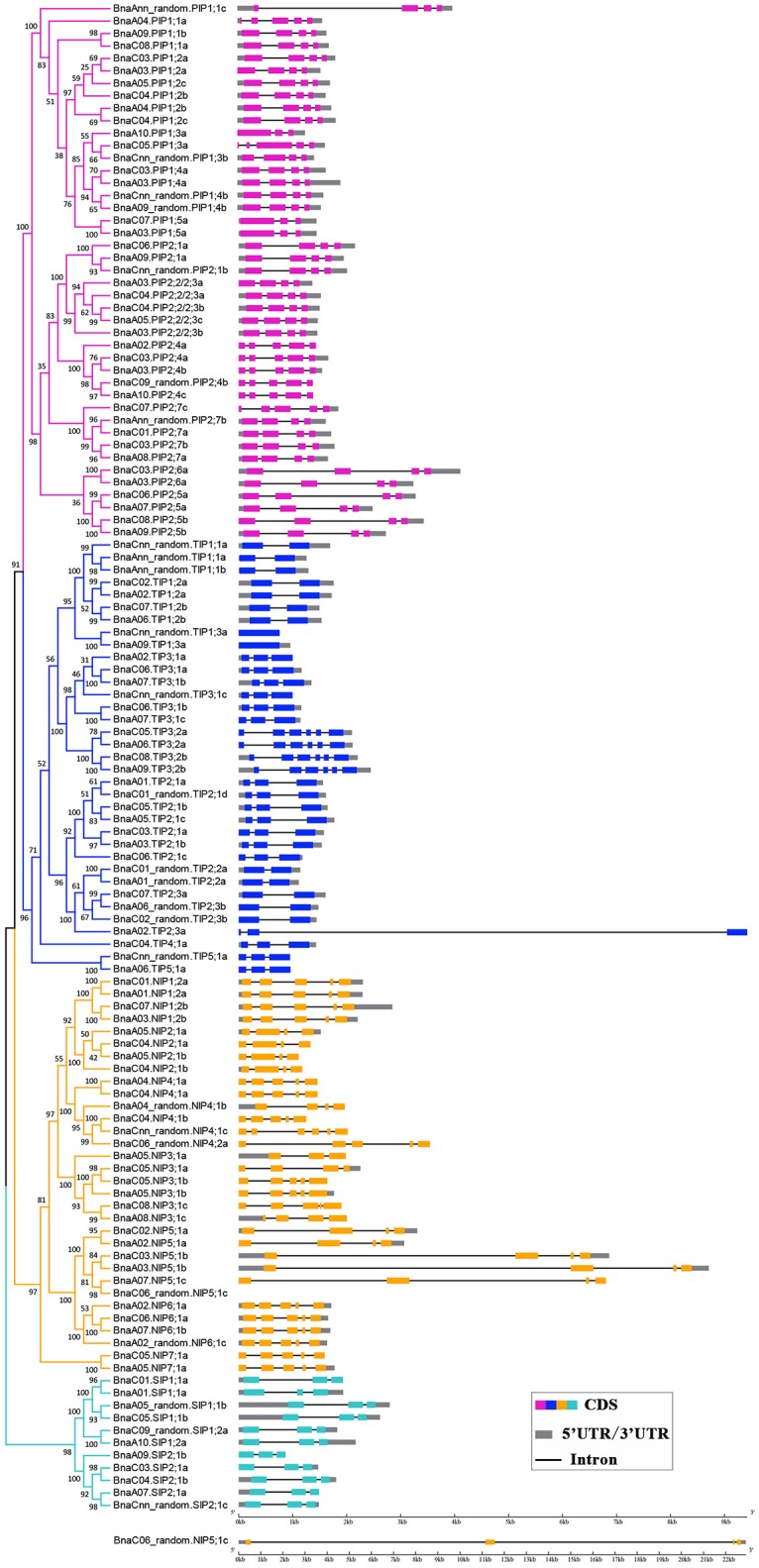
Gene structure of *BnaAQP*s. The exon-intron structures of the *BnAQP*s were determined by the alignments of coding sequences with corresponding genomic sequences, and the diagram was obtained using GSDS (Gene Structure Display Server 2.0) Web server. The purple, blue, yellow, and cyan rectangles represented the exons of *BnaPIP*s, *BnaTIP*s, *BnaNIP*s, and *BnaSIP*s, the gray rectangles represented the UTR and the black lines represented the introns, respectively. *BnaC06_random.NIP5;1c* was list alone because it had the longest gene length.

The availability of the *B. napus* gene sequence enabled us to analyze and compare the gene structures of all 121 *BnAQP*s (Figure 3). As a whole, there was considerable variation in intron number between different *BnaAQP* sub-families. The majority of *BnaPIP*s had three introns, although *BnaA10.PIP1;3a* and *BnaPIP1;5*s had two, and *BnaPIP2;4*s, *BnaA04.PIP1;1a* and *BnaC07.PIP2;7c* had four. Within the *BnaTIP* sub-family 17 genes had two and 12 had one intron. All the *BnaTIP3;2* genes had five introns, while two *BnaTIP1;3*s lacked an intron. Within the *BnaNIP* sub-family, 17 had four introns and 13, mostly *BnaNIP2;1*s and *BnaNIP5;1*s, had three introns. All SIP genes contained two introns. The gene structures of most *BnaAQP*s were conserved within a sub-family, and although there was some variation among homologous genes, they did not always show similarity amongst paralogous genes within the same sub-family. Additionally, the intron length changed frequently within the homologous genes of the isoforms of *BnaPIP2;5, BnaPIP2;6, BnaNIP4;1*, and *BnaNIP5;1*.

**Figure 4:**
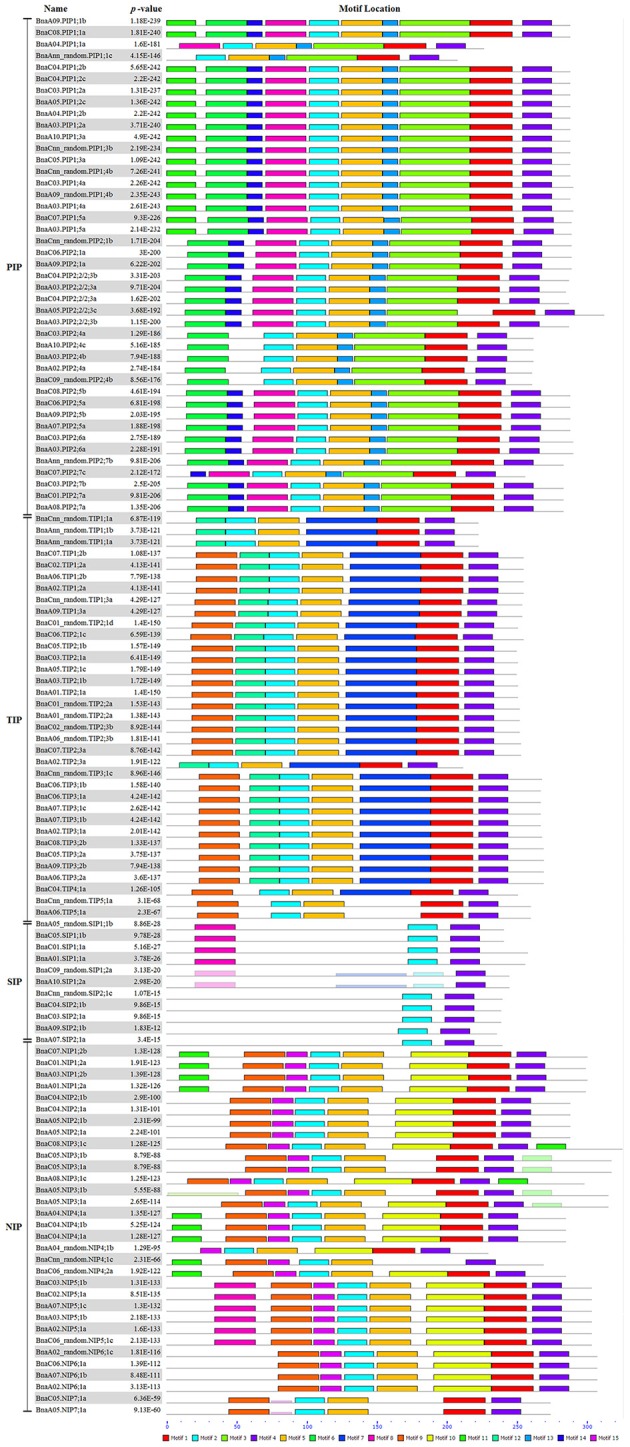
Distribution of conserved motifs in *BnaAQP*s. Conserved motifs of *BnaAQP*s were analyzed by MEME Web serve using the protein sequences of 121 full-length *BnaAQP*s. Fifteen conserved motifs were identified, and different motifs were distinguished by different colors.

In addition, the conserved domains of all BnaAQPs were analyzed and fifteen motifs identified as conserved (Figure 4). Generally, the BnaAQPs within each sub-group shared similar motif compositions, but among different sub-groups their motif compositions varied. Motif 4 was common among all BnaAQPs, and was determined as a conserved region of BnaAQPs (Supplementary Figure 2C). Motif 5 was conserved in BnaPIPs, BnaTIPs and BnaNIPs (Supplementary Figure 2D). Two NPA motifs of all BnaAQPs were included in motif 1 and motif 2 (Supplementary Figures 2A,B). The BnaSIP family had the lowest number of motifs, and shared the lowest similarities with the proteins of other BnaAQP sub-families. The number and type of motifs of BnaNIPs changed frequently, which indicated that the protein diversity of this sub-family was high in *B. napus*.

### The comparison of NPA motifs and ar/R selectivity filters in BnaAQPs

NPA motifs and ar/R selectivity filters in BnaAQPs were identified since they are important for determining the permeation of solution molecules (Table 3). Two NPA motifs were identical in both BnaTIPs and BnaPIPs and all the NPA motifs in each sub-family were conserved. Additionally, the ar/R selectivity filters of BnaPIPs were also identical within different sub-groups, but that of BnaTIPs changed frequently. The second NPA motifs in BnaSIPs were more conserved than the first. The first NPA motifs of *BnaSIP2;1*s were present in two styles (NPV and NPL). The NPA motif and ar/R selectivity filters of BnaNIPs showed the greatest variation, particularly the ar/R selectivity filters of *BnaNIP5;1*s (Table 3). Moreover, the amino acids of H5 (*BnaC06_random.NIP5;1c* and *BnaA07.NIP5;1c*: A, N, A, R) and LE1 (*BnaA03.NIP5;1b* and *BnaC03.NIP5;1b*: A, I, A, R) differed from that of *AtNIP5;1* (A, I, G, R) (Wallace and Roberts, 2004). In addition, we found that all the novel differences in the selectivity filters of *BnaAQPs* were paired in the corresponding A and C genomes (Table 3).

**Table 3:** Amino acid composition of the NPA motifs and ar/R selectivity filters of AQPs in *Brassica napus*.

**BnaAQP subgroup**	**NPA1**	**NPA2**	**H2**	**H5**	**LE1**	**LE2**
PIP1	NPA	NPA	F	H	T	R
PIP2	NPA	NPA	F	H	T	R
TIP1	NPA	NPA	H	I	A	V
TIP2	NPA	NPA	H	I	G	R
TIP3;1	NPA	NPA	H	I	A	R
TIP3;2	NPA	NPA	H	M	A	R
TIP4	NPA	NPA	H	I	A	R
TIP5	NPA	NPA	N	V	G	C
SIP1;1	NPT	NPA	I	V	P	I
SIP1;2	NPC	NPA	V	F	P	I
BnaA09.SIP2;1b	NPV	NPA	S	H	G	A
BnaCnn_random.SIP2;1c BnaC04.SIP2;1b BnaA07.SIP2;1a BnaC03.SIP2;1a	NPL	NPA	S	H	G	A
NIP1	NPA	NPG	W	V	A	R
NIP2	NPA	NPA	W	V	A	R
NIP3	NPA	NPA	W	I	A	R
NIP4	NPA	NPA	W	V	A	R
BnaA07.NIP5;1c BnaC06_random.NIP5;1c	NPS	NPV	A	N	A	R
BnaC03.NIP5;1b BnaA03.NIP5;1b	NPS	NPV	A	I	A	R
BnaA02.NIP5;1a BnaC02.NIP5;1a	NPS	NPV	A	I	G	R
NIP6	NPA	NPV	A	I	A	R
NIP7	NPS	NPA	A	V	G	R

### Gene expression of *BnaAQP*s in response to low B stress

**Figure 5:**
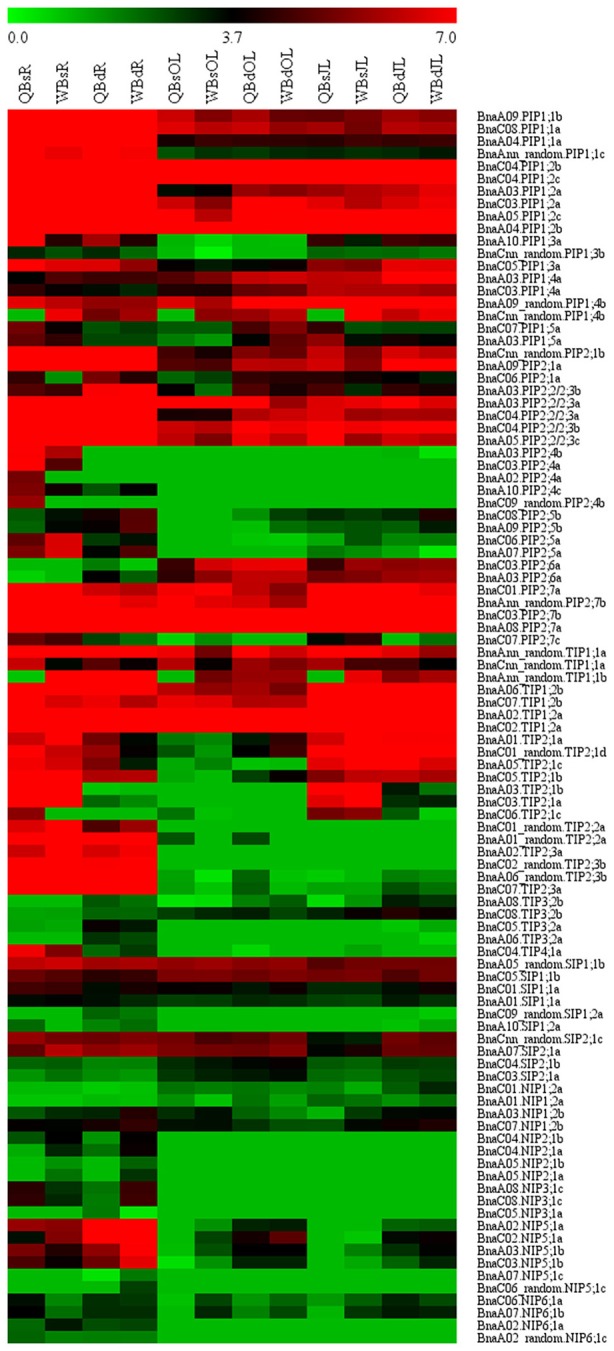
Expression profile of *BnaAQP*s in response to low boron stress in *Brassica napus*. The RPKM (reads per kilo bases per million reads) values of 99 *BnaAQP*s (non-expressed genes were not included) were obtained from the RNA-seq data, and then the RPKM values were converted into the logarithmic values. The heat map of expression profile of *BnaAQP*s in roots (R), old leaves (OL), and juvenile leaves (JL) was generated using the MeV program. The two cultivars of *Brassica napus*, QY10 (Q, B-efficient), and W10 (W, B-inefficient), were cultured in hydroponic solution with B deficient conditions (d, 0.25 μM) and B sufficient conditions (s, 25 μM) for 30 d.

RNA-seq. was used to investigate the transcriptional profile of all the *BnaAQP*s in contrasting B-efficient (“Qingyou10”) and B-inefficient (“Westar10”) cultivar under B sufficient and deficient conditions. A total of 99 *BnaAQP*s (81%) were up-regulated or down-regulated in roots and/or leaves of both cultivars. The heat map displayed the transcript abundance pattern of *BnaAQP*s in roots (R), old leaves (OL), and juvenile leaves (JL) (Figure 5).

Most of the *BnaPIP*s showed relatively higher expression levels in all the three tissues of B-efficient and B-inefficient cultivars compared with other sub-families of *BnaAQP*s. Moreover, approximately half of the *BnaPIP*s, e.g., *BnaPIP1;1*s, *BnaPIP1;2*s, and *BnaPIP2;2/2;3*s, had high expression levels in roots under the B sufficient condition; and they were dramatically down-regulated under low B stress in both cultivars (Figure 5 and Supplementary Table 3). A majority of *BnaPIP*s were expressed in all three tissues in both cultivars and both B levels, including *BnaPIP1;1*s, *BnaPIP1;2*s, *BnaPIP1;4*s (expect for *BnaCnn_random.PIP1;4b*), *BnaPIP2;1*s, *BnaPIP2;2/2;3*s, and *BnaPIP2;7*s (expect for *BnaC07.PIP2;7c*) (Figure 5 and Supplementary Table 3). However, several other *BnaPIP*s had tissue-preferential expression patterns in both cultivars, e.g., *BnaPIP1;3*s, *BnaPIP2;4*s, *BnaPIP2;5*s, and *BnaPIP2;6*s. Under B deprivation, the expression of *BnaCnn_random.PIP1;4b* was significantly up-regulated in all the three tissues of Qingyou10, but not in Westar10. However, the *BnaPIP2;4* members were strikingly down-regulated in the roots of the two cultivars, particularly in Qingyou10. In addition, *BnaC08.PIP2;5b* and *BnaA09.PIP2;5b* showed up-regulation, while *BnaC06.PIP2;*5a and *BnaA07.PIP2;5a* were down-regulated with B deprivation.

*BnaTIP1;1*s and *BnaTIP1;2*s showed high expression levels in all tissues, whereas *BnaTIP1;3*s, *BnaTIP3;1*s, and *BnaTIP5;1*s were rarely expressed in any tissues. *BnaTIP2;2*s and *BnaTIP2;3*s showed an obvious root-preferential expression pattern in both B-efficient and B-inefficient cultivars at both B levels (Figure 5 and Supplementary Table 3). Under B deprivation, *BnaC06.TIP2;1c, BnaC03.TIP2;1a*, and *BnaA03.TIP2;1b* were distinctly down-regulated in roots and leaves of the two cultivars, while *BnaC04.TIP4;1a* was only down-regulated in roots. The degree of down-regulation for *BnaC04.TIP4;1a* in Qingyou10 was much greater than that for Westar10. *BnaAnn_random.TIP1;1b* only showed significantly up-regulated at low B in Qingyou10.

The expression levels of all the *BnaSIP*s in all tissues had slightly changed under B deprivation, and there was no significant difference between the B-efficient and B-inefficient cultivars. Moreover, transcription of all *BnaSIP*s varied in all tissues, apart from *BnaA09.SIP2;1b* which had no transcripts detected in any tissue, and *BnaSIP1;2*s which was not transcribed in leaves.

In comparison with other *BnaAQPs*, the expression levels of *BnaNIP*s were generally relatively lower in all the three tissues of B-efficient and B-inefficient cultivars under both B conditions. *BnaA08.NIP3;1c, BnaC08.NIP3;1c, BnaA02_random.NIP6;1c, BnaA02.NIP6;1a* as well as *BnaNIP2;1*s showed a root-specific expression pattern in both cultivars at low and high B levels (Figure 5 and Supplementary Table 3). The transcription of *BnaC05.NIP3;1b, BnaA05.NIP3;1b, BnaNIP4;1*s, *BnaNIP4;2*s, and *BnaNIP7;1*s was not detected in any tissue in both cultivars. In contrast, the transcription of *BnaC03.NIP5;1b, BnaC02.NIP5;1a, BnaA03.NIP5;1b*, and *BnaA02.NIP5;1a* was induced at low B in all three tissues of both cultivars, especially in the roots. Interestingly, the level of transcription for all the *BnaNIP5;1*s mentioned above in the B-inefficient cultivar (Westar10) was significantly higher than that in the B-efficient cultivar (Qingyou10).

## Discussion

### AQP characterization in *Brassica napus*

Aquaporin is an abundant and high diversity protein family in plants, which facilitates transport of diverse small neutral molecules such as H_2_O, H_2_O_2_, boric acid, arsenic acid, silicic acid, urea and glycerol across membrane (Wallace et al., 2002; Ma et al., 2006, 2008; Takano et al., 2006; Hove and Bhave, 2011). Previously, *AQP* genes have been identified within the Brassicaceae, with 60 in the diploid A genome of *B. rapa* (Tao et al., 2014), 67 in the diploid C genome of *B. oleracea* (Diehn et al., 2015), and 35 in *A. thaliana* (Johanson et al., 2001). *B. napus* is a recent allopolyploid that originated by combining the intact genomes of *B. oleracea* and *B. rapa*. Gene replication and chromosome rearrangement in *B. rapa* and *B. oleracea* is therefore expected to result in a conserved gene distribution of AQP in the respective A and C chromosomes of *B. napus*. In this study, 121 full-length AQP family genes were identified in *B. napus* (Table 1), only slightly fewer than the sum of those reported in *B. oleracea* (Diehn et al., 2015) and *B. rapa* (Tao et al., 2014). Due to the origin and evolutionary independence of the two diploids (*B. rapa* and *B. oleracea*) over the past 4.6 MYA (Liu et al., 2014), the chromosomal location of *BnaAQP*s in the A genome are not completely conserved in homoeologous regions of the C genome (Figure 2).

In this study, we were able to allocate *BnaAQP*s according to the four well-established sub-families (43 *PIP*s, 35 *TIP*s, 32 *NIP*s, and 11 *SIP*s). As expected, no *XIP* sub-family was detected consistent with their absence in *Arabidopsis, B. oleracea* and *B. rapa* (Johanson et al., 2001; Tao et al., 2014; Diehn et al., 2015). There was also evidence of strong conservation within each BnaAQP sub-family since the allopolyploidisation of *B. napus* ~7,500 years ago, with membership closely matching the sum of *B. rapa* (23 *PIP*s, 16 *TIP*s, 15 *NIP*s, and 6 *SIP*s) and *B. oleracea* (25 *PIP*s, 19 *TIP*s, 17 *NIP*s, and 6 *SIP*s).

Comparison of collinearity between *B. napus* and orthologous *Arabidopsis* genes showed that 63% (76 of 121) of the AQP genes were present in 17 syntenic blocks, and these genes were distributed in proportion among the three ancestral *Brassica* sub-genomes of LF, MF1, and MF2 (Table 2). There was evidence of gene loss within the annotated sub-genomes of *B. napus* compared with the genomes of *B. rapa* and *B. oleracea*. The AQP gene membership of the LF, MF1, and MF2 sub-genomes in *B. napus* was 33, 25, and 18, however, there were 24, 16, and 13 AQP gene members in *B. rapa* and 30, 18, and 16 in *B. oleracea*, respectively (Tao et al., 2014; Diehn et al., 2015). The *BnaAQP*s density in the LF sub-genome was much higher than that of the other two MFs in *B. napus*, which is in proportion with the total number of all genes previously allocated to these categories for *B. rapa* and *B. oleracea* (Liu et al., 2014; Tao et al., 2014; Diehn et al., 2015). The two-step triplication that has been proposed for diploid *Brassica* species is based on evidence that a tetraploidization event was followed by substantial genome fractionation (MF1 and MF2), and subsequent hybridization with a third, less-fractionated sub-genome (LF) (Wang et al., 2011; Tang et al., 2012; Cheng et al., 2013). Most homoeologous *AQP* genes are represented in both A and C genomes of *B. napus* (Table 2). However, 17 AQPs were present only in the A or C genome, such as *BnaA04.PIP1;1a*. This may have arisen due to AQP gene loss or chromosome rearrangement either during the evolution of diploid *Brassica* species or subsequent to the formation of *B. napus* (Nicolas et al., 2007; Jeonghwan et al., 2009; Liu et al., 2014). Although 121 AQP genes were identified as full-length *BnaAQP*s (Table 1), 15 had an incomplete protein structure (Supplementary Table 1), mostly lacking fragments containing the conserved motifs corresponding to the NPA and ar/R selectivity filters.

The NPA motifs and ar/R selectivity filters of *Bna*AQPs showed variation among sub-families (Table 3). Within the PIP sub-families these were highly conserved in *A. thaliana, B. rapa, B. oleracea*, and *B. napus*. In addition, compared with those in barley (Tombuloglu et al., 2015), common bean (Ariani and Gepts, 2015) and rubber tree (Zhi et al., 2015), the NPA motifs and ar/R selectivity filters of *BnaPIP*s were also highly conserved, which suggested that the PIPs have been subject to strong selection in different plant taxonomic lineages. The Val (V) and Ile (I) residues near to P3 site have been considered as the conserved amino acids for water channel activity (Suga and Maeshima, 2004), and their differentiation can be used to distinguish PIP1s and PIP2s. In this study, all BnaPIP2s had the Val (V) and Ile (I) residues near P3 site, except for BnaPIP2;5s. However, all PIP1s had the Ile (I) and Ile (I) residues (Supplementary Data 1), which suggested that the BnaPIPs could be separated to BnaPIP1s and BnaPIP2s by the two amino acids.

All TIP sub-families of *B. napus, B. rapa, B. oleracea*, and *A. thaliana* shared two identical NPA motifs in HB and HE, although there still existed some differences in the ar/R selectivity filters. In *Arabidopsis*, the ar/R selectivity filters of TIPs were divided into 3 large subgroups (group I, group II and group III), with group II consisting of group IIA and group IIB (Wallace and Roberts, 2004). AtTIP3;2s belongs to group IIB, although all BnaTIP3;2s had a novel compositions of ar/R selectivity filters (H, M, A, R) (Table 3), which were the same as those of *B. rapa* and *B. oleracea*, but different from *A. thaliana* (H, I, A, R) (Tao et al., 2014; Diehn et al., 2015). The ar/R selectivity filters in TIP5s of the four species were considerably diverged from other TIPs (Table 3) (Tao et al., 2014; Diehn et al., 2015), indicating that TIP5s probably have a special function for solute permeation. Additionally, BnaTIP5;1s had a high pI and a distinct subcellular localization from the other *BnaTIP* members (Table 1). TIPs usually facilitate H_2_O, NH_3_ and urea transport (Wudick et al., 2009). Overexpression of *AtTIP5;1* in *A. thaliana* significantly increases the tolerance to B toxicity (Pang et al., 2010), and the vacuolar compartmentation could explain the high B tolerance of *Arabidopsis* overexpression lines.

*BnaNIP*s and *BnaSIP*s showed larger variation in the third alanine of the NPA motifs, and a greater diversity in ar/R selectivity filters than *BnaPIP*s and *BnaTIP*s (Table 3), suggesting that they may have acquired a more specialized function for substrate absorption than the latter. In *A. thaliana*, the AtNIPs showed a large-scale permeation for substrates such as H_2_O, H_2_O_2_, As, B, Si, urea, and glycerol, which was consistent with their abundant amino acid constitutions of NPAs and ar/R selectivity filters of NIP sub-family (Mitaniueno et al., 2011).

The NPAs and ar/R selectivity filters of *BnaAQP*s were similar to those of the combination of *BraAQP*s and *BolAQP*s, but the specific ar/R selectivity filters in *BraA.NIP4.c* and *BolC.NIP4.d* (W, S, A, R) (Diehn et al., 2015) had been lost during the allopolyploidisation or subsequent domestication of *B. napus* (Table 3). The ar/R selectivity filters of BnaTIP3;2s and BnaNIP5;1s differed from those of their homologous genes in *A. thaliana*, which is consistent with the finding in *B. oleracea* (Tao et al., 2014). Moreover, all the novel differences in the selectivity filters of *BnaAQP*s were paired in the corresponding A and C genomes (Table 3), which suggested that the differentiation may have occurred before the species divergence of *B. rapa* and *B. oleracea*.

The majority of *BnaAQP*s were conserved both in the number and the position of introns among different paralogous genes in *B. napus* (Figure 3), although the intron lengths varied widely, such as *BnaPIP2;5*s, *BnaPIP2;6*s, *BnaTIP2;3*s, *BnaNIP4;1*s, and *BnaNIP5;1*s. The intron length of these paralogous genes had possibly undergone some changes since the relatively recent emergence of *B. napus*. In addition, the conserved domains of the four sub-families were significantly different, especially the SIP sub-family, which is in agreement with the result that the SIP sub-family was distant from the other three sub-families presented in the phylogenetic tree (Figure 1). However, the constitution of the predicted conserved domains of *BnaAQP*s within each sub-family was very similar apart from the NIP sub-family.

### *BnaAQP*s and B homeostasis in *Brassica napus*

Recent studies have shown that *AtNIP5;1, AtNIP6;1, AtTIP5;1, OsPIP2;4, OsPIP2;7, OsPIP1;3, OsPIP2;6*, and *HvNIP2:1* play an important role in plant B homeostasis (Takano et al., 2006; Tanaka et al., 2008; Pang et al., 2010; Kumar et al., 2014; Mosa et al., 2015). In many cases, PIPs function as the water channels which mediate efficient water uptake in plant cells (Alexandersson et al., 2005; Boursiac et al., 2008; Mahdieh et al., 2008). In this study, the gene expression levels of most *BnaPIP*s were relatively higher than other *BnaAQP*s in all the tissues, especially in the roots under both B conditions (Figure 5 and Supplementary Table 3), which might be consistent with their functions as the water channel. Under B deficiency, the expression of most *BnaPIP*s was down-regulated in the roots of both cultivars (Figure 5 and Supplementary Table 3), indicating that they might be involved in the responses to the B deprivation in *B. napus*. Additionally, *BnaPIP2;4*s had high expression levels in the roots of both cultivars under the normal B condition, which was similar to their orthologues in *Arabidopsis* (Alexandersson et al., 2010), however, the gene expression of *PIP2;4*s in *B. rapa* and *B. oleracea* was not detected.

*BnaTIP1;1*s (except for *BnaAnn_random.TIP1;1b*) and *BnaTIP1;2*s displayed high expression levels in all tissues of both cultivars at two B levels and their expression was repressed in the roots with low B stress (Figure 5 and Supplementary Table 3), although recent studies report that the losses of *AtTIP1;1* and *AtTIP1;2* do not influence the growth of *A. thaliana* significantly (Schüssler et al., 2008; Beebo et al., 2009), thus suggesting that new functions for these genes in plants still need to be discovered. *BnaTIP2;2*s and *BnaTIP2;3*s clearly showed root-specific expression patterns, which was similar to that of *B. oleracea* (Diehn et al., 2015), *B. rapa* (Tao et al., 2014), and *Arabidopsis* (Alexandersson et al., 2010). Moreover, the *BnaTIP2;2*s and *BnaTIP2;3*s was strongly down-regulated at low B in both cultivars (Figure 5).

The gene expression levels of all the *BnaSIP*s were relatively low in all the tissues of both B-efficient and B-inefficient cultivars under low B condition (Figure 5). Hence, the function of BnaSIP genes was independent of the B supplement in this study. As a whole, at both B levels, the expression levels of *BnaNIP*s of both cultivars were lower than that of the other three sub-families in all tissues (Figure 5). Four members of *BnaNIP5;1*s (*BnaA02.NIP5;1a, BnaC02.NIP5;1a, BnaA03.NIP5;1b*, and *BnaC03.NIP5;1b*) exhibited significantly up-regulation whether in the root or in the leaves at low B stress, suggesting that they were not only involved in the B absorption in the root, but also in the response to low B in the shoot of *B. napus*.

Some AQP genes have tissue-specific expression characteristics. For instance, *AtTIP1;3*s, *AtTIP5;1*s, *AtNIP4;1*s, and *AtNIP4;2*s have been assigned as pollen-specific genes (Soto et al., 2008; Giorgio et al., 2016) and *AtNIP7;1*s showed an anther-specific expression pattern (Li et al., 2011). Thus, it is not surprising that transcription of *BnaTIP1;3*s, *BnaTIP3;1*s, *BnaTIP5;1*s, *BnaNIP4;1*s, *BnaNIP4;2*s, and *BnaNIP7;1*s was not detected in roots, old leaves and juvenile leaves of 30 d seedlings of QY10 and W10 under both B conditions in this study.

Significant differences in the transcription of several AQP genes between B-efficient cultivar QY10 and B-inefficient cultivar W10 were observed. This included *BnaCnn_random.PIP1;4b, BnaC04.TIP4;1a, BnaAnn_random.TIP1;1b, BnaC03.NIP5;1b, BnaC02.NIP5;1a, BnaA03.NIP5;1b*, and *BnaA02.NIP5;1a* (Figure 5 and Supplementary Table 3). Of these, we have previously mapped *BnaA03.NIP5;1b* as a B-efficient QTL *qBEC-A3a* detected in *B. napus* (Hua et al., 2016a). Moreover, the other member *BnaC02.NIP5;1a*, was also identified as a B-efficient QTL *qBEC-C2a*, and possibly facilitates efficient boron absorption under low B condition in *B. napus* (Hua et al., 2016b).

In summary, we identified and characterized 121 full-length AQP genes which belong to the PIP, TIP, SIP and NIP sub-families. The characteristics of gene structure, selectivity filters and transcriptional patterns for most AQP genes of *B. napus* were similar to those of *B. rapa, B. oleracea*, and *Arabidopsis*. We confirmed the identity and relationship of two candidate genes (*BnaA03.NIP5;1b* and *BnaC02.NIP5;1a*) underlying B-efficient QTL regions in *B. napus*. We found that most *BnaPIP*s and *BnaTIP*s had high expression levels in all the tissues of B-efficient and B-inefficient cultivars under both B conditions, especially in roots. Moreover, under low B condition, the transcription of these genes was down-regulated in roots although their responses to low B stress in leaves were only slight or with no change.

## Author contributions

Conceptual and experiment designs by LS and DY; Experiments were conducted by DY; RNA-seq data analysis performed by WL; Reagents/materials/analysis tools were contributed by LS, and the report was written by DY, YH, GK, FX, and LS. All the authors have commented, read and approved the final manuscript.

### Conflict of interest statement

The authors declare that the research was conducted in the absence of any commercial or financial relationships that could be construed as a potential conflict of interest.

## Supplementary material

Supplementary Figure 1 Putative MW and PI of BnaPIPs, BnaSIPs, BnaNIPs, and BnaTIPs in *Brassica napus*. The genes in the four sub-families were distinguished by different legends.

Supplementary Figure 2 WebLogo plot of the consensus motifs in BnaAQPs. The amino-acid residues of the consensus motifs in FASTA format were obtained from the MEME Web and WebLogo plot graphs were generated using the amino acid sequences, **(A)** Motif 1; **(B)** motif 2; **(C)** motif 4; **(D)** motif 5.

Supplementary Table 1 Gene location and description of incomplete *BnaAQP*s.

Supplementary Table 2 *BnaAQP* genes without chromosome localization information.

Supplementary Table 3 The RPKM values of the 121 *BnaAQP*s.

Supplementary Data 1 The multiple sequence alignment of 121 BnaAQPs. The yellow highlighted amino acids represent the residues near P3 site. The letters in a red font below P1 to P5 indicate the amino acid residues in the Froger's position.
