# Supplementary figures and images for: Genome-Wide Identification and Characterization of the Aquaporin Gene Family and Transcriptional Responses to Boron Deficiency in Brassica napus
Source: Front Plant Sci. 2017 Aug 2;8:1336. doi: 10.3389/fpls.2017.01336 (PMC5539139; doi:10.3389/fpls.2017.01336)

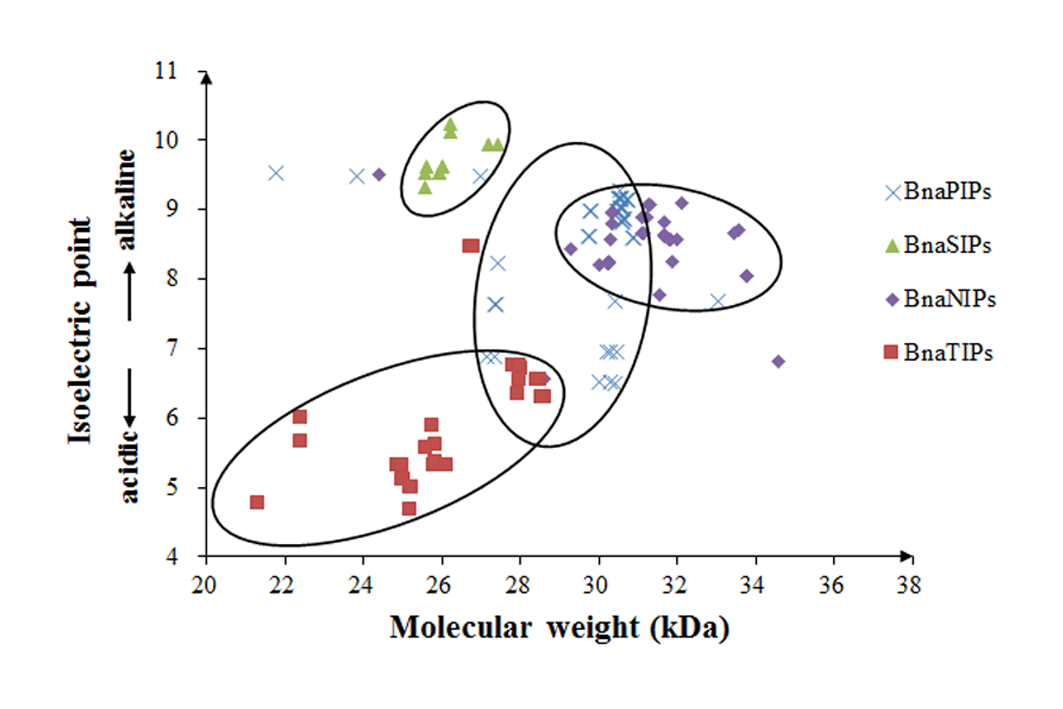

Supplement: Supplementary Figure 1 — Putative MW and PI of BnaPIPs, BnaSIPs, BnaNIPs, and BnaTIPs in Brassica napus. The genes in the four sub-families were distinguished by different legends. [file Image1.TIF]

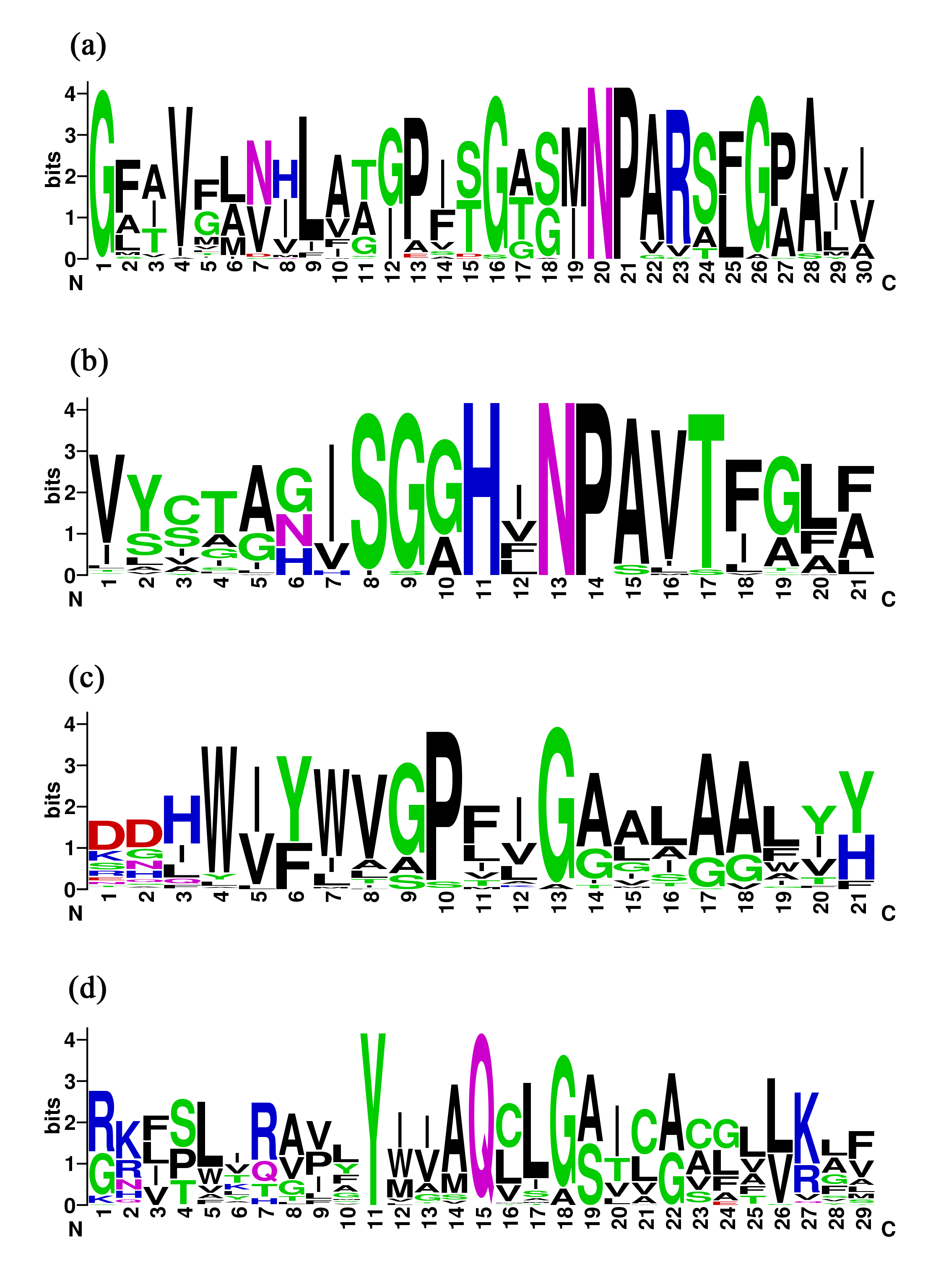

Supplement: Supplementary Figure 2 — WebLogo plot of the consensus motifs in BnaAQPs. The amino-acid residues of the consensus motifs in FASTA format were obtained from the MEME Web and WebLogo plot graphs were generated using the amino acid sequences, (A) Motif 1; (B) motif 2; (C) motif 4; (D) motif 5. [file Image2.TIF]
