# Supplementary material for: Genome-Wide Identification and Characterization of the Aquaporin Gene Family and Transcriptional Responses to Boron Deficiency in Brassica napus
Source: Front Plant Sci. 2017 Aug 2;8:1336. doi: 10.3389/fpls.2017.01336 (PMC5539139; doi:10.3389/fpls.2017.01336)
Supplement: Supplementary Table 1 — Gene location and description of incomplete BnaAQPs. [file Table1.DOCX]

Supplementary table 1. Gene location and description of incomplete *BnaAQP*s

| **At isoform** | **Gene ID** | ***B. napus* ID** | **BnaAQP name** | **Location** | **Description** |
| --- | --- | --- | --- | --- | --- |
| *AtPIP2;1* | *At3g53420* | *BnaA07g02320D* | *BnaA07.PIP2;1* | 1908203..1908602 | Only one transmembrane domain |
| *AtPIP2;2 AtPIP2;3* | *At2g37170 At2g37180* | *BnaC03g72010D* | *BnaC03_random.PIP2;2/2;3* | 477555..478475 | Lost the first two transmembrane domains |
|  |  | *BnaA05g07290D* | *BnaA05.PIP2;2/2;3* | 3928469..3929205 | Lost the first two transmembrane domains |
|  |  | *BnaC03g20550D* | *BnaC03.PIP2;2/2;3* | 10900413..10900722 | Only one transmembrane domain |
|  |  | *BnaC01g33380D* | *BnaC01.PIP2;2* | 32568205..32568687 | No transmembrane domain |
|  |  | *No annotation* | *BnaC03.PIP2;2/2;3* | 53409685..53410167 | No transmembrane domain |
|  |  | *BnaC04g16700D* | *BnaC04.PIP2;3* | 14725444..14725926 | No transmembrane domain |
|  |  | *BnaA06g16680D* | *BnaA06.PIP2;3* | 9404396..9404878 | No transmembrane domain |
| *AtTIP1;3* | *At4g01470* | *BnaC02g27930D* | *BnaC02.TIP1;3* | 26026343..26026772 | Only three transmembrane domains in the middle region |
| *AtTIP4;1* | *At2g25810* | *BnaA04g28330D* | *BnaA04_random.TIP4;1* | 761668..762301 | No transmembrane domain |
|  |  | *BnaA04g15110D* | *BnaA04.TIP4;1* | 12606669..12610923 | Only the last three transmembrane domains |
| *AtSIP2;1* | *At3g56950* | *BnaC08g27820D* | *BnaC08.SIP2;1* | 28643134..28643545 | An early termination mutation result in two truncated genes |
|  |  | *BnaC08g27810D* | *BnaC08.SIP2;1* | 28642633..28642986 |  |
|  |  | *BnaA04g02840D* | *BnaA04.SIP2;1* | 1883108..1883738 | Only one transmembrane domain(A long tandem repeat in upstream of gene) |
| *AtNIP4;1* | *At5g37810* | *BnaC04g34460D* | *BnaC04.NIP4;1* | 36060984..36062996 | Lost the first two transmembrane domains |
