# Supplementary material for: Genome-Wide Identification and Characterization of the Aquaporin Gene Family and Transcriptional Responses to Boron Deficiency in Brassica napus
Source: Front Plant Sci. 2017 Aug 2;8:1336. doi: 10.3389/fpls.2017.01336 (PMC5539139; doi:10.3389/fpls.2017.01336)
Supplement: Supplementary Table 2 — BnaAQP genes without chromosome localization information. [file Table2.DOCX]

Supplementary table 2. *BnaAQP* genes without chromosome localization information

| ***B. napus* ID** | **AQP name** | **Chromosome** |
| --- | --- | --- |
| ***BnaPIP*s** |  |  |
| *BnaAnng23190D* | *BnaAnn_random.PIP1;1c* | Ann_random |
| *BnaCnng08780D* | *BnaCnn_random.PIP1;3b* | Cnn_random |
| *BnaCnng02360D* | *BnaCnn_random.PIP1;4b* | Cnn_random |
| *BnaA09g51960D* | *BnaA09_random.PIP1;4b* | A09_random |
| *BnaCnng31040D* | *BnaCnn_random.PIP2;1b* | Cnn_random |
| *BnaC03g72010D* | *BnaC03_random.PIP2;2/2;3** | C03_random |
| *BnaC09g53920D* | *BnaC09_random.PIP2;4b* | C09_random |
| *BnaAnng11630D* | *BnaAnn_random.PIP2;7b* | Ann_random |
| ***BnaTIPs*** |  |  |
| *BnaCnng24720D* | *BnaCnn_random.TIP1;1a* | Cnn_random |
| *BnaAnng24130D* | *BnaAnn_random.TIP1;1b* | Ann_random |
| *BnaAnng22640D* | *BnaAnn_random.TIP1;1a* | Ann_random |
| *BnaCnng01570D* | *BnaCnn_random.TIP1;3a* | Cnn_random |
| *BnaC01g44580D* | *BnaC01_random.TIP2;1d* | C01_random |
| *BnaC01g41690D* | *BnaC01_random.TIP2;2a* | C01_random |
| *BnaA01g35340D* | *BnaA01_random.TIP2;2a* | A01_random |
| *BnaC02g46870D* | *BnaC02_random.TIP2;3b* | C02_random |
| *BnaA06g40020D* | *BnaA06_random.TIP2;3b* | A06_random |
| *BnaCnng50290D* | *BnaCnn_random.TIP3;1c* | Cnn_random |
| *BnaA04g28330D* | *BnaA04_random.TIP4;1** | A04_random |
| *BnaCnng15220D* | *BnaCnn_random.TIP5;1a* | Cnn_random |
| ***BnaSIPs*** |  |  |
| *BnaA05g37440D* | *BnaA05_random.SIP1;1b* | A05_random |
| *BnaC09g54320D* | *BnaC09_random.SIP1;2a* | C09_random |
| *BnaCnng20470D* | *BnaCnn_random.SIP2;1c* | Cnn_random |
| ***BnaNIPs*** |  |  |
| *BnaA04g27980D* | *BnaA04_random.NIP4;1b* | A04_random |
| *BnaCnng65250D* | *BnaCnn_random.NIP4;1c* | Cnn_random |
| *BnaC06g42210D* | *BnaC06_random.NIP4;2a* | C06_random |
| *BnaC06g42490D* | *BnaC06_random.NIP5;1c* | C06_random |
| *BnaC06g42500D* |  |  |
| *BnaA02g36290D* | *BnaA02_random.NIP6;1c* | A02_random |
