# Supplementary material for: Genome-Wide Identification and Characterization of the Aquaporin Gene Family and Transcriptional Responses to Boron Deficiency in Brassica napus
Source: Front Plant Sci. 2017 Aug 2;8:1336. doi: 10.3389/fpls.2017.01336 (PMC5539139; doi:10.3389/fpls.2017.01336)
Supplement: Supplementary Table 3 — The RPKM values of the 121 BnaAQPs. [file Table3.DOCX]

Supplementary table 3. The RPKM values of the 121 *BnaAQP*s

| **Gene name** | **Root** | | | | **Old Leaf** | | | | **Juvenile Leaf** | | | |
| --- | --- | --- | --- | --- | --- | --- | --- | --- | --- | --- | --- | --- |
|  | **+B** | | **-B** | | **+B** | | **-B** | | **+B** | | **-B** | |
|  | **Q** | **W** | **Q** | **W** | **Q** | **W** | **Q** | **W** | **Q** | **W** | **Q** | **W** |
| ***BnaA09.PIP1;1b*** | 1399.65 | 988.91 | 513.34 | 742.42 | 79.12 | 40.79 | 58.36 | 31.70 | 30.09 | 37.73 | 52.98 | 43.13 |
| ***BnaC08.PIP1;1a*** | 1335.07 | 843.12 | 402.28 | 507.51 | 96.64 | 68.10 | 77.89 | 48.57 | 55.16 | 35.05 | 65.57 | 58.92 |
| ***BnaA04.PIP1;1a*** | 359.62 | 200.90 | 268.73 | 246.81 | 15.15 | 21.22 | 19.56 | 19.13 | 18.04 | 22.35 | 20.04 | 21.49 |
| ***BnaAnn_random.PIP1;1c*** | 177.17 | 106.23 | 136.44 | 118.23 | 4.68 | 6.92 | 6.23 | 7.63 | 8.03 | 7.07 | 7.62 | 9.18 |
| ***BnaC04.PIP1;2b*** | 1404.83 | 615.73 | 1041.89 | 1038.59 | 497.58 | 352.78 | 464.11 | 354.02 | 404.69 | 270.73 | 644.32 | 501.77 |
| ***BnaC04.PIP1;2c*** | 1657.13 | 804.83 | 1125.64 | 1216.12 | 383.38 | 361.08 | 371.18 | 307.78 | 341.51 | 404.53 | 572.29 | 545.22 |
| ***BnaC03.PIP1;2a*** | 474.63 | 307.20 | 313.10 | 429.67 | 81.03 | 42.31 | 145.00 | 135.19 | 100.08 | 64.58 | 91.21 | 113.15 |
| ***BnaA05.PIP1;2c*** | 875.51 | 726.04 | 931.06 | 1039.17 | 167.20 | 67.26 | 212.77 | 126.68 | 321.48 | 188.31 | 358.54 | 199.53 |
| ***BnaA04.PIP1;2b*** | 2391.75 | 1129.73 | 995.36 | 1256.56 | 523.08 | 260.32 | 423.33 | 357.94 | 517.23 | 335.16 | 434.33 | 367.90 |
| ***BnaA03.PIP1;2a*** | 202.72 | 299.85 | 207.51 | 301.43 | 10.42 | 12.89 | 50.05 | 42.21 | 51.92 | 64.27 | 74.00 | 103.14 |
| ***BnaA10.PIP1;3a*** | 192.23 | 17.68 | 54.63 | 16.90 | 1.09 | 0.53 | 0.00 | 0.95 | 20.17 | 8.64 | 22.53 | 20.38 |
| ***BnaCnn_random.PIP1;3b*** | 7.95 | 4.89 | 7.58 | 3.54 | 0.00 | 0.14 | 0.00 | 0.00 | 3.67 | 3.30 | 3.52 | 2.92 |
| ***BnaC05.PIP1;3a*** | 139.33 | 95.35 | 93.08 | 46.43 | 12.55 | 8.94 | 11.03 | 13.53 | 44.19 | 38.94 | 102.59 | 99.83 |
| ***BnaCnn_random.PIP1;4b*** | 0.00 | 109.95 | 34.77 | 53.61 | 0.00 | 45.16 | 50.33 | 68.19 | 1.00 | 224.51 | 74.80 | 109.85 |
| ***BnaC03.PIP1;4a*** | 18.95 | 13.17 | 10.55 | 7.86 | 16.78 | 16.05 | 31.07 | 32.81 | 67.47 | 61.56 | 61.49 | 54.45 |
| ***BnaA09_random.PIP1;4b*** | 100.41 | 69.76 | 47.45 | 48.47 | 92.77 | 58.99 | 143.45 | 159.60 | 436.75 | 206.45 | 192.32 | 190.94 |
| ***BnaA03.PIP1;4a*** | 11.67 | 25.16 | 21.18 | 22.00 | 26.18 | 38.14 | 74.59 | 82.53 | 70.40 | 74.14 | 167.41 | 128.15 |
| ***BnaC07.PIP1;5a*** | 34.69 | 13.32 | 4.76 | 6.27 | 4.28 | 4.21 | 23.88 | 39.63 | 19.09 | 4.90 | 5.68 | 5.83 |
| ***BnaA03.PIP1;5a*** | 30.00 | 19.30 | 5.40 | 5.41 | 2.73 | 1.90 | 11.56 | 29.05 | 45.85 | 10.22 | 15.10 | 11.26 |
| ***BnaCnn_random.PIP2;1b*** | 863.78 | 709.53 | 379.50 | 581.72 | 23.10 | 15.13 | 40.53 | 31.15 | 75.53 | 37.54 | 88.95 | 67.96 |
| ***BnaC06.PIP2;1a*** | 19.65 | 2.07 | 36.69 | 17.37 | 3.75 | 5.86 | 19.89 | 17.86 | 17.63 | 14.38 | 12.17 | 8.89 |
| ***BnaA09.PIP2;1a*** | 1179.36 | 1027.22 | 584.19 | 855.16 | 26.54 | 24.18 | 68.99 | 66.36 | 85.96 | 42.21 | 162.56 | 148.97 |
| ***BnaC04.PIP2;2/2;3b*** | 2370.76 | 1074.41 | 1301.99 | 1522.80 | 78.98 | 66.36 | 152.61 | 108.99 | 170.23 | 113.08 | 163.64 | 167.22 |
| ***BnaA03.PIP2;2/2;3a*** | 3902.66 | 1899.91 | 1971.76 | 2582.24 | 269.67 | 203.86 | 151.21 | 51.03 | 94.94 | 105.91 | 153.44 | 93.43 |
| ***BnaC04.PIP2;2/2;3a*** | 337.58 | 156.37 | 259.35 | 377.33 | 15.21 | 15.78 | 62.81 | 79.65 | 96.22 | 52.91 | 59.15 | 57.22 |
| ***BnaA05.PIP2;2/2;3c*** | 1848.51 | 438.25 | 718.44 | 779.01 | 70.49 | 38.39 | 109.50 | 72.63 | 134.32 | 51.18 | 83.67 | 69.10 |
| ***BnaA03.PIP2;2/2;3b*** | 26.54 | 21.30 | 123.47 | 201.37 | 12.52 | 3.27 | 25.48 | 15.54 | 23.78 | 7.47 | 19.09 | 15.06 |
| ***BnaC03.PIP2;4a*** | 133.58 | 26.46 | 0.00 | 0.00 | 0.00 | 0.00 | 0.00 | 0.00 | 0.00 | 0.00 | 0.00 | 0.00 |
| ***BnaA10.PIP2;4c*** | 39.06 | 14.19 | 4.57 | 11.57 | 0.00 | 0.00 | 0.00 | 0.00 | 0.00 | 0.00 | 0.00 | 0.00 |
| ***BnaA03.PIP2;4b*** | 124.63 | 60.78 | 0.00 | 0.00 | 0.00 | 0.00 | 0.00 | 0.00 | 0.00 | 0.00 | 1.11 | 0.34 |
| ***BnaA02.PIP2;4a*** | 36.20 | 0.00 | 0.00 | 0.00 | 0.00 | 0.00 | 0.00 | 0.00 | 0.00 | 0.00 | 0.00 | 0.00 |
| ***BnaC09_random.PIP2;4b*** | 49.44 | 0.93 | 0.00 | 0.00 | 0.00 | 0.00 | 0.00 | 0.00 | 0.00 | 0.00 | 0.00 | 0.00 |
| ***BnaC08.PIP2;5b*** | 4.41 | 10.06 | 15.11 | 27.27 | 0.00 | 0.00 | 2.02 | 5.79 | 7.61 | 6.19 | 7.89 | 16.66 |
| ***BnaC06.PIP2;5a*** | 29.55 | 91.24 | 6.30 | 9.99 | 0.00 | 0.99 | 0.76 | 0.69 | 1.34 | 4.59 | 2.38 | 2.67 |
| ***BnaA09.PIP2;5b*** | 3.82 | 11.05 | 12.92 | 27.73 | 0.00 | 0.00 | 0.90 | 3.22 | 4.11 | 4.62 | 4.02 | 9.00 |
| ***BnaA07.PIP2;5a*** | 38.52 | 92.01 | 11.25 | 25.69 | 0.00 | 0.00 | 0.00 | 0.00 | 2.80 | 2.14 | 1.35 | 0.29 |
| ***BnaC03.PIP2;6a*** | 0.00 | 0.00 | 2.69 | 0.79 | 20.72 | 85.39 | 105.37 | 110.66 | 20.64 | 50.38 | 44.30 | 47.74 |
| ***BnaA03.PIP2;6a*** | 0.57 | 0.00 | 11.77 | 4.01 | 18.71 | 45.08 | 71.79 | 71.22 | 34.47 | 39.65 | 51.72 | 55.32 |
| ***BnaAnn_random.PIP2;7b*** | 476.85 | 229.19 | 124.99 | 102.02 | 130.60 | 100.27 | 89.66 | 54.53 | 224.80 | 149.34 | 177.00 | 140.27 |
| ***BnaC07.PIP2;7c*** | 31.43 | 23.10 | 5.78 | 3.33 | 0.56 | 2.20 | 0.00 | 0.00 | 10.86 | 18.97 | 0.96 | 3.21 |
| ***BnaC03.PIP2;7b*** | 369.10 | 246.21 | 409.60 | 267.90 | 368.60 | 387.85 | 286.16 | 202.46 | 334.78 | 259.20 | 537.77 | 462.67 |
| ***BnaC01.PIP2;7a*** | 260.73 | 169.38 | 81.13 | 63.86 | 116.49 | 130.17 | 70.46 | 42.38 | 138.59 | 131.50 | 137.75 | 106.64 |
| ***BnaA08.PIP2;7a*** | 305.82 | 386.62 | 479.16 | 327.62 | 215.02 | 267.50 | 232.15 | 239.86 | 271.15 | 492.96 | 540.33 | 593.76 |
| ***BnaCnn_random.TIP1;1a*** | 77.24 | 11.03 | 27.81 | 13.25 | 65.49 | 13.02 | 50.94 | 39.55 | 56.05 | 23.20 | 23.11 | 12.46 |
| ***BnaAnn_random.TIP1;1b*** | 0.78 | 1513.61 | 520.74 | 847.19 | 0.00 | 34.62 | 50.25 | 50.19 | 0.00 | 107.87 | 40.85 | 57.29 |
| ***BnaAnn_random.TIP1;1a*** | 6473.01 | 1574.49 | 2185.31 | 1533.08 | 111.37 | 34.63 | 142.63 | 82.90 | 398.24 | 142.54 | 98.01 | 48.93 |
| ***BnaC07.TIP1;2b*** | 155.62 | 91.04 | 110.49 | 63.76 | 110.87 | 99.26 | 63.05 | 65.56 | 191.05 | 303.53 | 219.23 | 183.08 |
| ***BnaC02.TIP1;2a*** | 1877.75 | 1639.16 | 991.30 | 1158.24 | 169.62 | 145.38 | 268.41 | 251.76 | 616.09 | 648.93 | 657.46 | 568.43 |
| ***BnaA06.TIP1;2b*** | 985.86 | 434.12 | 311.96 | 446.47 | 67.11 | 46.29 | 56.17 | 37.08 | 219.74 | 299.18 | 178.05 | 145.73 |
| ***BnaA02.TIP1;2a*** | 1922.19 | 1205.19 | 1180.29 | 715.10 | 153.20 | 174.75 | 393.63 | 343.22 | 1023.17 | 817.93 | 1173.41 | 1178.65 |
| ***BnaCnn_random.TIP1;3a*** | 0.00 | 0.00 | 0.00 | 0.00 | 0.00 | 0.00 | 0.00 | 0.00 | 0.00 | 0.00 | 0.00 | 0.33 |
| ***BnaA09.TIP1;3a*** | 0.00 | 0.00 | 0.00 | 0.00 | 0.00 | 0.00 | 0.00 | 0.00 | 0.00 | 0.00 | 0.00 | 0.00 |
| ***BnaC01_random.TIP2;1d*** | 139.02 | 76.15 | 49.07 | 12.72 | 4.57 | 1.85 | 12.20 | 21.44 | 129.91 | 135.98 | 184.89 | 148.49 |
| ***BnaC06.TIP2;1c*** | 44.28 | 0.00 | 0.00 | 0.00 | 3.04 | 0.88 | 0.00 | 0.00 | 35.28 | 43.41 | 4.22 | 0.70 |
| ***BnaC05.TIP2;1b*** | 142.84 | 137.76 | 61.66 | 64.67 | 1.41 | 0.00 | 5.77 | 13.07 | 39.57 | 70.52 | 73.34 | 56.72 |
| ***BnaC03.TIP2;1a*** | 297.56 | 191.45 | 3.60 | 2.22 | 0.00 | 0.00 | 0.00 | 0.00 | 97.04 | 209.75 | 7.16 | 8.51 |
| ***BnaA05.TIP2;1c*** | 115.52 | 86.04 | 38.66 | 8.71 | 1.35 | 2.46 | 0.84 | 0.00 | 100.42 | 148.20 | 133.37 | 91.22 |
| ***BnaA03.TIP2;1b*** | 312.54 | 185.06 | 0.76 | 0.00 | 0.00 | 0.00 | 0.00 | 0.00 | 141.48 | 252.35 | 9.21 | 3.02 |
| ***BnaA01.TIP2;1a*** | 79.49 | 132.54 | 34.66 | 11.17 | 2.87 | 2.32 | 9.20 | 19.52 | 84.82 | 149.70 | 122.46 | 123.03 |
| ***BnaC01_random.TIP2;2a*** | 93.25 | 135.20 | 25.34 | 50.85 | 0.00 | 0.00 | 0.00 | 0.00 | 0.00 | 0.00 | 0.00 | 0.00 |
| ***BnaA01_random.TIP2;2a*** | 727.78 | 827.18 | 172.25 | 255.62 | 4.58 | 0.00 | 5.23 | 0.00 | 0.00 | 0.00 | 0.00 | 0.00 |
| ***BnaC02_random.TIP2;3b*** | 230.43 | 411.29 | 123.42 | 225.46 | 0.00 | 0.00 | 0.00 | 0.00 | 0.00 | 0.00 | 0.00 | 0.00 |
| ***BnaA06_random.TIP2;3b*** | 7003.26 | 1213.30 | 1444.20 | 801.21 | 1.53 | 0.34 | 3.72 | 0.84 | 0.68 | 0.00 | 2.55 | 2.09 |
| ***BnaC07.TIP2;3a*** | 5538.82 | 4654.62 | 2168.80 | 2858.94 | 1.61 | 0.80 | 4.17 | 1.01 | 1.48 | 1.44 | 4.80 | 3.13 |
| ***BnaA02.TIP2;3a*** | 79.42 | 367.83 | 88.84 | 117.79 | 0.00 | 0.00 | 0.00 | 0.00 | 0.00 | 0.00 | 0.00 | 0.00 |
| ***BnaCnn_random.TIP3;1c*** | 0.00 | 0.00 | 0.00 | 0.00 | 0.00 | 0.00 | 0.00 | 0.00 | 0.00 | 0.00 | 0.00 | 0.00 |
| ***BnaC06.TIP3;1b*** | 0.00 | 0.00 | 0.00 | 0.00 | 0.00 | 0.00 | 0.00 | 0.00 | 0.00 | 0.00 | 0.00 | 0.00 |
| ***BnaC06.TIP3;1a*** | 0.00 | 0.00 | 0.00 | 0.00 | 0.00 | 0.00 | 0.00 | 0.00 | 0.00 | 0.00 | 0.00 | 0.00 |
| ***BnaA07.TIP3;1c*** | 0.00 | 0.00 | 0.00 | 0.00 | 0.00 | 0.00 | 0.00 | 0.00 | 0.00 | 0.00 | 0.00 | 0.00 |
| ***BnaA07.TIP3;1b*** | 0.00 | 0.00 | 0.00 | 0.00 | 0.00 | 0.00 | 0.00 | 0.00 | 0.00 | 0.00 | 0.00 | 0.00 |
| ***BnaA02.TIP3;1a*** | 0.00 | 0.00 | 0.00 | 0.00 | 0.00 | 0.00 | 0.00 | 0.00 | 0.00 | 0.00 | 0.00 | 0.00 |
| ***BnaC08.TIP3;2b*** | 1.47 | 1.54 | 3.69 | 3.58 | 5.84 | 7.76 | 8.64 | 5.56 | 8.14 | 13.16 | 17.41 | 13.96 |
| ***BnaC05.TIP3;2a*** | 1.56 | 1.35 | 13.19 | 9.14 | 0.00 | 0.00 | 0.00 | 0.00 | 0.00 | 0.00 | 0.76 | 1.33 |
| ***BnaA08.TIP3;2b*** | 0.00 | 0.00 | 4.44 | 3.15 | 0.33 | 0.42 | 2.58 | 4.13 | 0.43 | 1.88 | 8.72 | 6.87 |
| ***BnaA06.TIP3;2a*** | 0.00 | 0.00 | 6.86 | 5.27 | 0.00 | 0.00 | 0.00 | 0.00 | 0.00 | 0.00 | 0.00 | 0.56 |
| ***BnaC04.TIP4;1a*** | 112.47 | 40.31 | 3.56 | 6.21 | 0.00 | 0.00 | 0.51 | 0.00 | 0.00 | 1.45 | 0.00 | 0.00 |
| ***BnaCnn_random.TIP5;1a*** | 0.00 | 0.00 | 0.00 | 0.00 | 0.00 | 0.00 | 0.00 | 0.00 | 0.00 | 0.00 | 0.00 | 0.00 |
| ***BnaA06.TIP5;1a*** | 0.00 | 0.00 | 0.00 | 0.00 | 0.00 | 0.00 | 0.00 | 0.00 | 0.00 | 0.00 | 0.00 | 0.00 |
| ***BnaA05_random.SIP1;1b*** | 70.46 | 81.00 | 54.46 | 58.66 | 46.75 | 52.47 | 42.75 | 46.73 | 27.36 | 34.93 | 35.41 | 34.50 |
| ***BnaC05.SIP1;1b*** | 32.33 | 26.70 | 18.00 | 20.17 | 38.19 | 38.61 | 33.75 | 39.08 | 35.33 | 37.96 | 25.77 | 34.52 |
| ***BnaC01.SIP1;1a*** | 22.73 | 20.76 | 10.41 | 15.19 | 12.86 | 13.12 | 9.32 | 13.98 | 7.77 | 7.79 | 10.36 | 14.67 |
| ***BnaA01.SIP1;1a*** | 10.73 | 11.44 | 10.29 | 7.73 | 5.85 | 6.10 | 7.76 | 7.19 | 5.69 | 5.33 | 9.10 | 6.91 |
| ***BnaC09_random.SIP1;2a*** | 0.00 | 0.00 | 3.71 | 2.81 | 0.00 | 0.00 | 0.00 | 0.00 | 0.00 | 0.00 | 0.00 | 0.71 |
| ***BnaA10.SIP1;2a*** | 3.49 | 0.00 | 2.39 | 3.37 | 0.00 | 0.00 | 0.00 | 0.00 | 0.00 | 0.00 | 0.85 | 1.38 |
| ***BnaCnn_random.SIP2;1c*** | 48.85 | 35.95 | 33.38 | 39.47 | 35.63 | 24.26 | 27.30 | 34.19 | 9.95 | 8.38 | 34.81 | 28.94 |
| ***BnaC04.SIP2;1b*** | 3.71 | 4.12 | 2.40 | 2.50 | 7.36 | 9.44 | 11.19 | 12.98 | 4.26 | 3.83 | 6.20 | 5.85 |
| ***BnaC03.SIP2;1a*** | 1.91 | 3.10 | 2.13 | 1.80 | 6.41 | 8.27 | 9.16 | 10.65 | 3.46 | 3.07 | 4.75 | 5.40 |
| ***BnaA09.SIP2;1b*** | 0.00 | 0.00 | 0.00 | 0.00 | 0.00 | 0.00 | 0.00 | 0.00 | 0.00 | 0.00 | 0.00 | 0.00 |
| ***BnaA07.SIP2;1a*** | 29.92 | 65.81 | 45.30 | 51.79 | 38.26 | 35.31 | 33.73 | 41.10 | 11.30 | 15.70 | 32.19 | 30.59 |
| ***BnaC07.NIP1;2b*** | 12.66 | 11.42 | 15.04 | 19.44 | 9.99 | 10.30 | 6.92 | 4.77 | 4.73 | 10.96 | 13.89 | 16.44 |
| ***BnaC01.NIP1;2a*** | 0.00 | 0.00 | 0.70 | 0.87 | 3.11 | 2.64 | 2.95 | 2.23 | 2.51 | 1.29 | 4.12 | 7.93 |
| ***BnaA03.NIP1;2b*** | 4.44 | 7.42 | 8.06 | 17.05 | 7.23 | 10.23 | 3.89 | 2.25 | 1.13 | 6.20 | 10.94 | 11.29 |
| ***BnaA01.NIP1;2a*** | 0.78 | 0.76 | 0.00 | 0.92 | 2.05 | 0.84 | 3.10 | 1.58 | 3.69 | 3.08 | 2.03 | 3.30 |
| ***BnaC04.NIP2;1b*** | 4.60 | 11.98 | 1.92 | 12.63 | 0.00 | 0.00 | 0.00 | 0.00 | 0.00 | 0.00 | 0.00 | 0.00 |
| ***BnaC04.NIP2;1a*** | 1.27 | 7.92 | 3.20 | 13.30 | 0.00 | 0.00 | 0.00 | 0.00 | 0.00 | 0.00 | 0.00 | 0.00 |
| ***BnaA05.NIP2;1b*** | 0.00 | 1.96 | 0.00 | 3.85 | 0.00 | 0.00 | 0.00 | 0.00 | 0.00 | 0.00 | 0.00 | 0.00 |
| ***BnaA05.NIP2;1a*** | 0.00 | 3.08 | 0.00 | 6.86 | 0.00 | 0.00 | 0.00 | 0.00 | 0.00 | 0.00 | 0.00 | 0.00 |
| ***BnaC08.NIP3;1c*** | 18.68 | 7.80 | 2.77 | 21.43 | 0.00 | 0.00 | 0.00 | 0.00 | 0.00 | 0.00 | 0.00 | 0.00 |
| ***BnaC05.NIP3;1b*** | 0.00 | 0.00 | 0.00 | 0.00 | 0.00 | 0.00 | 0.00 | 0.00 | 0.00 | 0.00 | 0.00 | 0.00 |
| ***BnaC05.NIP3;1a*** | 0.00 | 0.00 | 2.81 | 0.30 | 0.00 | 0.00 | 0.00 | 0.00 | 0.00 | 0.00 | 0.00 | 0.00 |
| ***BnaA08.NIP3;1c*** | 17.70 | 6.80 | 3.03 | 20.86 | 0.00 | 0.00 | 0.00 | 0.00 | 0.00 | 0.00 | 0.00 | 0.00 |
| ***BnaA05.NIP3;1b*** | 0.00 | 0.00 | 0.00 | 0.00 | 0.00 | 0.00 | 0.00 | 0.00 | 0.00 | 0.00 | 0.00 | 0.00 |
| ***BnaA05.NIP3;1a*** | 0.00 | 0.00 | 0.00 | 0.00 | 0.00 | 0.00 | 0.00 | 0.00 | 0.00 | 0.00 | 0.00 | 0.00 |
| ***BnaA04.NIP4;1a*** | 0.00 | 0.00 | 0.00 | 0.00 | 0.00 | 0.00 | 0.00 | 0.00 | 0.00 | 0.00 | 0.00 | 0.00 |
| ***BnaC04.NIP4;1b*** | 0.00 | 0.00 | 0.00 | 0.00 | 0.00 | 0.00 | 0.00 | 0.00 | 0.00 | 0.00 | 0.00 | 0.00 |
| ***BnaC04.NIP4;1a*** | 0.00 | 0.00 | 0.00 | 0.00 | 0.00 | 0.00 | 0.00 | 0.00 | 0.00 | 0.00 | 0.00 | 0.00 |
| ***BnaA04_random.NIP4;1b*** | 0.00 | 0.00 | 0.00 | 0.00 | 0.00 | 0.00 | 0.00 | 0.00 | 0.00 | 0.00 | 0.00 | 0.00 |
| ***BnaCnn_random.NIP4;1c*** | 0.00 | 0.00 | 0.00 | 0.00 | 0.00 | 0.00 | 0.00 | 0.00 | 0.00 | 0.00 | 0.00 | 0.00 |
| ***Bna*C06_random*.NIP4;2a*** | 0.00 | 0.00 | 0.00 | 0.00 | 0.00 | 0.00 | 0.00 | 0.00 | 0.00 | 0.00 | 0.00 | 0.00 |
| ***BnaC03.NIP5;1b*** | 24.61 | 13.10 | 33.17 | 100.63 | 0.39 | 1.96 | 8.31 | 8.26 | 0.00 | 1.34 | 3.83 | 8.12 |
| ***BnaC02.NIP5;1a*** | 10.27 | 40.46 | 133.31 | 285.32 | 0.00 | 5.69 | 14.96 | 27.86 | 0.00 | 0.76 | 10.76 | 13.30 |
| ***BnaA07.NIP5;1c*** | 0.00 | 0.00 | 0.33 | 2.83 | 0.00 | 0.00 | 0.00 | 0.00 | 0.00 | 0.00 | 0.00 | 0.00 |
| ***BnaA03.NIP5;1b*** | 36.83 | 16.74 | 45.47 | 124.08 | 0.93 | 4.77 | 12.03 | 12.08 | 0.00 | 2.50 | 7.05 | 11.44 |
| ***BnaA02.NIP5;1a*** | 49.77 | 43.55 | 170.46 | 324.89 | 0.00 | 2.00 | 8.14 | 8.79 | 0.00 | 0.00 | 3.79 | 4.17 |
| ***BnaC06_random.NIP5;1c*** | 0.00 | 0.00 | 0.00 | 5.84 | 0.00 | 0.00 | 0.00 | 0.00 | 0.00 | 0.00 | 0.00 | 0.00 |
| ***BnaA02_random.NIP6;1c*** | 3.78 | 2.49 | 2.65 | 2.58 | 0.00 | 0.00 | 0.00 | 0.00 | 0.00 | 0.00 | 0.00 | 0.00 |
| ***BnaC06.NIP6;1a*** | 10.24 | 2.37 | 7.11 | 6.46 | 0.86 | 5.55 | 1.90 | 3.71 | 1.79 | 4.03 | 8.22 | 5.12 |
| ***BnaA07.NIP6;1b*** | 12.18 | 3.34 | 7.32 | 7.27 | 0.00 | 7.79 | 2.49 | 5.24 | 1.10 | 6.61 | 9.15 | 8.41 |
| ***BnaA02.NIP6;1a*** | 3.57 | 9.15 | 4.96 | 5.52 | 0.00 | 0.00 | 0.00 | 0.00 | 0.00 | 0.00 | 0.00 | 0.00 |
| ***BnaC05.NIP7;1a*** | 0.00 | 0.00 | 0.00 | 0.00 | 0.00 | 0.00 | 0.00 | 0.00 | 0.00 | 0.00 | 0.00 | 0.00 |
| ***BnaA05.NIP7;1a*** | 0.00 | 0.00 | 0.00 | 0.00 | 0.00 | 0.00 | 0.00 | 0.00 | 0.00 | 0.00 | 0.00 | 0.00 |
