# Supplementary material for: Genome-Wide Identification and Characterization of the Aquaporin Gene Family and Transcriptional Responses to Boron Deficiency in Brassica napus
Source: Front Plant Sci. 2017 Aug 2;8:1336. doi: 10.3389/fpls.2017.01336 (PMC5539139; doi:10.3389/fpls.2017.01336)
Supplement: Supplementary Data 1 — The multiple sequence alignment of 121 BnaAQPs. The yellow highlighted amino acids represent the residues near P3 site. The letters in a red font below P1 to P5 indicate the amino acid residues in the Froger's position. [file DataSheet1.DOCX]

**Supplementary data 1.** The multiple sequence alignment of 121 BnaAQPs. The yellow highlighted amino acids represent the residues near P3 site. The letters in a red font below P1 to P5 indicate the amino acid residues in the Froger's position.

BnaAnn_random.TIP1_1b --------------------------------------------------

BnaAnn_random.TIP1_1a --------------------------------------------------

BnaCnn_random.TIP1_1a --------------------------------------------------

BnaC07.TIP1_2b ---------------------------------MPIRNIAIGGVQGEV-- 15

BnaA06.TIP1_2b ---------------------------------MPIRNIAIGGVQEEV-- 15

BnaC02.TIP1_2a ---------------------------------MPTRNIAIGGVQEEV-- 15

BnaA02.TIP1_2a ---------------------------------MPTRNIAIGGVQEEV-- 15

BnaCnn_random.TIP1_3a ---------------------------------MAINRIAIG-TPGEA-- 14

BnaA09.TIP1_3a ---------------------------------MAINRIAIG-TPGEA-- 14

BnaCnn_random.TIP3_1c -------------------------------MATSARRAYGFGRADEA-- 17

BnaA02.TIP3_1a -------------------------------MATSARRAYGFGRADEA-- 17

BnaC06.TIP3_1a -------------------------------MATSAHRAYAFGRADEA-- 17

BnaA07.TIP3_1b -------------------------------MATSAHRAYAFGRADEA-- 17

BnaC06.TIP3_1b -------------------------------MAASTVRTYGFGRADEA-- 17

BnaA07.TIP3_1c -------------------------------MAASTVRTYGFGRADEA-- 17

BnaC08.TIP3_2b -------------------------------MATYARRTYGFGRADEA-- 17

BnaA09.TIP3_2b -------------------------------MATYARRTYGFGRADEA-- 17

BnaC05.TIP3_2a -------------------------------MATYARRTYGFGRADEA-- 17

BnaA06.TIP3_2a -------------------------------MATYARRTYGFGRTDEA-- 17

BnaC01_random.TIP2_1d ------------------------------------MAGVAFGSFDDS-- 12

BnaA01.TIP2_1a ------------------------------------MAAVAFGSFDDS-- 12

BnaC05.TIP2_1b ------------------------------------MAGVAFGSFDDS-- 12

BnaA05.TIP2_1c ------------------------------------MAGLAFGSFDDS-- 12

BnaC03.TIP2_1a ------------------------------------MAGIAFGSFDDS-- 12

BnaA03.TIP2_1b ------------------------------------MAGIAFGSFDDS-- 12

BnaC06.TIP2_1c ------------------------------------MAGIDF-SCEDS-- 11

BnaC01_random.TIP2_2a ------------------------------------MVKIAIGSLGDS-- 12

BnaA01_random.TIP2_2a ------------------------------------MVKIAIGSLGDS-- 12

BnaC02_random.TIP2_3b ------------------------------------MVKIEVGSVGDS-- 12

BnaA02.TIP2_3a ------------------------------------MVKIEVGSVSDS-- 12

BnaA06_random.TIP2_3b ------------------------------------MVKIAVGSLGDS-- 12

BnaC07.TIP2_3a ------------------------------------MVKIAVGSLGDS-- 12

BnaC04.TIP4_1a ------------------------------------MKKIDLGNHREA-- 12

BnaCnn_random.TIP5_1a --------------------------------MRRMIPTTFSSKFQGA-- 16

BnaA06.TIP5_1a --------------------------------MRRMIPTTFSSKFQGA-- 16

BnaCnn_random.PIP2_1b ----------------MAKDVEAVSGEGFQTRDYQDPPPAPLFDPEEL-- 32

BnaA09.PIP2_1a ----------------MAKDVEAVSGEGFQTRDYQDPPPAPLFDPAEL-- 32

BnaC06.PIP2_1a ----------------MAKDVEAVAGEGFQSRDYQDPPPAPLFDPEEL-- 32

BnaC04.PIP2_2/2_3b ----------------MAKEVEGA--EGFASRDYEDPPPTPFFDAEEL-- 30

BnaA05.PIP2_2/2_3c ----------------MAKEVEGA--EGFASRDYEDPPPTPFFDAEEL-- 30

BnaC04.PIP2_2/2_3a ----------------MAKDVEGA--EGFAARDYEDPPPTPFFDAEEL-- 30

BnaA03.PIP2_2/2_3a ----------------MAKDVEGA--EGVTARDYEDPPPTPFFDAEEL-- 30

BnaA03.PIP2_2/2_3b ----------------MAKDAEGA--EGFATRDYQDPPPAPFFDAEEL-- 30

BnaC03.PIP2_4a ----------------MAKDLEVQEGGATAARDYQDPPPAPLFDMEEL-- 32

BnaA03.PIP2_4b ----------------MAKDLEVQEGGAMAARDYQDPPPAPLFDMEEL-- 32

BnaA10.PIP2_4c ----------------MAKDLEVQDGGATAARDYVDPPPAPLLDMEEF-- 32

BnaC09_random.PIP2_4b ----------------MAKDLEVQEGGATAARDYVDPPPAPLLDMEEF-- 32

BnaA02.PIP2_4a ----------------MAKDLEVQEG--RAARDYQDPPPAPLFDMEEL-- 30

BnaC08.PIP2_5b ----------------MTKEVVGEKG-SFSGKDYQDPPPEPLFDATEL-- 31

BnaA09.PIP2_5b ----------------MTKEVVGEKG-SFSGKDYQDPPPEPLFDATEL-- 31

BnaC06.PIP2_5a ----------------MTKDVAGEKG-SFSGKDYQDPPPEPLFDATEL-- 31

BnaA07.PIP2_5a ----------------MTKDVAGEKG-SFSGKDYQDPPPEPLFDATEL-- 31

BnaC03.PIP2_6a ----------------MSTELTEEE--SLSGKDYQDPPRVKIFEAREL-- 30

BnaA03.PIP2_6a ----------------MSTDLTEEE--SLSGKDYQDPPRVKIFEAREL-- 30

BnaAnn_random.PIP2_7b ----------------MSKEVSEEGQTHSHGKDYVDPPPAPLLDMGEL-- 32

BnaC01.PIP2_7a ----------------MSKEVSEEGQTHSHGKDYVDPPPAPLLDMGEL-- 32

BnaC03.PIP2_7b ----------------MSKEVSEEGHTQSHGKDYVDPPPAPFLDMGEL-- 32

BnaA08.PIP2_7a ----------------MSKEVSEEGHTQSHGKDYVDPPPAPFLDMGEL-- 32

BnaC07.PIP2_7c ----------------MYKITGHD-KRKTHN------------------- 14

BnaC07.PIP1_5a --MEGKEEDVNVGANKFPERQPIGTAAQTEGKDYKEPPPAPFFEPGEL-- 46

BnaA03.PIP1_5a --MEGKEEDVNVGANKFPERQPIGTAAQTEGKDYKEPPPAPFFEPGEL-- 46

BnaCnn_random.PIP1_3b --MEGKEEDVRVGANKFPERQPIGTSAQSD-KDYKEPPPAPLFEPGEL-- 45

BnaC05.PIP1_3a --MEGKEEDVRVGANKFPERQPIGTSAQTD-KDYKEPPPAPLFEPGEL-- 45

BnaA10.PIP1_3a --MEGKEEDVRVGANKFPERQPIGTSAQTD-KDYKEPPPAPLFEPGEL-- 45

BnaCnn_random.PIP1_4b --MEGKEEDVRVGANKFPERQPIGTSAQSD-KDYKEPPPAPLFEPGEL-- 45

BnaA09_random.PIP1_4b --MEGKEEDVRVGANKFPERQPIGTSAQSD-KDYKEPPPAPLFEPGEL-- 45

BnaC03.PIP1_4a --MEGKEEDVRVGANKFPERQPIGTSAQSD-KDYKEPPPAPLFEPGEL-- 45

BnaA03.PIP1_4a --MEGKEEDVRVGANKFPERQPIGTSAQSD-KDYKEPPPAPLFEPGEL-- 45

BnaA09.PIP1_1b --MEGKEEDVRVGANKFPERQPIGTSAQSD-KDYNEPPPAPLFEPGEL-- 45

BnaC08.PIP1_1a --MEGKEEDVRVGANKFPERQPIGTSAQSD-KDYNEPPPAPLFEPGEL-- 45

BnaA04.PIP1_1a --MEGKEEDVR--------------------------------------- 9

BnaAnn_random.PIP1_1c --------------------------------------------------

BnaC04.PIP1_2b --MEGKEEDVRVGANKFPERQPIGTSAQSD-KDYKEPPPAPLFEPGEL-- 45

BnaA05.PIP1_2c --MEGKEEDVRVGANKFPERQPIGTSAQSD-KDYKEPPPAPLFEPGEL-- 45

BnaC03.PIP1_2a --MEGKEEDVRVGANKFPERQPIGTSAQSD-KDYKEPPPAPLFEPGEL-- 45

BnaA03.PIP1_2a --MEGKEEDVRVGANKFPERQPIGTSAQSD-KDYKEPPPAPLFEPGEL-- 45

BnaC04.PIP1_2c --MEGKEEDVRVGANKFPERQPIGTSAQSD-KDYKEPPPAPLFEPGEL-- 45

BnaA04.PIP1_2b --MEGKEEDVRVGANKFPERQPIGTSAQSD-KDYKEPPPAPLFEPGEL-- 45

BnaC07.NIP1_2b -MAEISGNGHGDSKEGAVMVNINQEVELQQQQKEAIHTTKSMKKQDSV-- 47

BnaA03.NIP1_2b -MAEISGNGHGDSKEGAVMVNINQEAELQQQQKEAIHTTKSMKKQDSV-- 47

BnaC01.NIP1_2a -MAEISGNGHGNAREGAVVVNINEEHERQQ-HKEAIHISKPIKKQDSL-- 46

BnaA01.NIP1_2a -MADISGNGHGDAREGAVVVNINEEHEHQQ-HKEAIHISKPMKKQDSL-- 46

BnaC04.NIP2_1b -MDDIS---VGKSNHGNVVVLNIQAPPVSK-------TSLPSSPPTSP-- 37

BnaA05.NIP2_1b -MDDIS---VSKSNHGNVVVLNIQAPPVSK-------TSLPSSPPTSP-- 37

BnaC04.NIP2_1a -MDDIS---VSKSNHGNVVVLNIQAPPVSK-------TSFPSSPSTSP-- 37

BnaA05.NIP2_1a -MDDIS---VSKSNHGNVVVLNIQAPPVSK-------TSFPSSPSTSP-- 37

BnaA04.NIP4_1a ----MTSHVEEIEEEEISKIEKGKGKDCHR-----GIETVICTSPTTV-- 39

BnaC04.NIP4_1a ----MTSHVEEIEEEEISKIEKGKGKDCHR-----GIETVICTSPTTV-- 39

BnaC04.NIP4_1b ----MTSHGEGIEEEQISRIEKGIEKDCHG-----GIETIICTSPSIV-- 39

BnaC06_random.NIP4_2a ----MTSHGEGIEEEKISRIEKGIEKDCHG-----GIERVICSSPSIV-- 39

BnaA04_random.NIP4_1b --------------------------------------------------

BnaC08.NIP3_1c --------------MAEISDTTPQTQTVTFDIEDGGSGGDSRSPGISR-- 34

BnaA08.NIP3_1c --------------MIMSRNIISQKR---------------RKHSL---- 17

BnaC05.NIP3_1b MSKPLTFSEKSILSMAEISSITVRTQTTILNIEDGRSSGDPRSPDSPC-- 48

BnaC05.NIP3_1a MSKPLTFSEKSILSMAEISSITVRTQTTILNIEDGRSSGDPRSPDSPC-- 48

BnaA05.NIP3_1b MSKPLTFSEKSILSMAEISGITVRTQTTILNIEDGRSSGDSRLPDSPC-- 48

BnaA05.NIP3_1a -----------------MANITGQTQIANLDIEDGRSGGGSRSQDSPR-- 31

BnaC03.NIP5_1b -MSPPEAEMGAVAVTAPPTPGTPGGPLITGMR----VDSMSFDHRKPIPP 45

BnaA03.NIP5_1b -MSPPEAEMGAVAVTAPPTPGTPGGPLITGMR----VDSMSFDHRKPIPP 45

BnaC02.NIP5_1a -MGPTEAEMGAVAVTAPPTPGTPGGPLITGMR----VDSMSFDHRKPMPP 45

BnaA02.NIP5_1a -MAPTEAEMGAVAVTAPPTPGTPGGPLITGMR----VDSMSFDHRKPMPP 45

BnaA07.NIP5_1c -MSPPEAEMGAVAVTAPPKPGTPRGPLITGMR----VDSVSFDHRKPIPP 45

BnaC06_random.NIP5_1c -MSPPEAEMGAVAVTAPPKPGTPRGPLITGMR----VDSMSFDHRKPIPP 45

BnaA02_random.NIP6_1c -MD--HEEIPSMPSTPATTPGTPGARLFGGFDGKRSGHNGRYTPKSLLKS 47

BnaA02.NIP6_1a -MD--HEEIPSMPSTPATTPGTPGAPLFGGFDGKRSGHNGRYTPKSLLKS 47

BnaC06.NIP6_1a -MD--HEEIPSMPSTPATTPGTPGAPLFGGFEGKRNGHNGKYTPKSILKS 47

BnaA07.NIP6_1b -MD--HEEIPSMPSTPATTPGTPGAPLFGGFEGKRNGHNGKYTPKSILKS 47

BnaC05.NIP7_1a -----------MNVEVRSRVFDQEAGSTLSSLRDGDPSTQRLFRCIPY-- 37

BnaA05.NIP7_1a -----------MNVEVRSRVFDQEAGSTLSSLRDGDLSTQRLFRCIPY-- 37

BnaA05_random.SIP1_1b --------------------------------------------------

BnaC05.SIP1_1b --------------------------------------------------

BnaC01.SIP1_1a --------------------------------------------------

BnaA01.SIP1_1a --------------------------------------------------

BnaC09_random.SIP1_2a --------------------------------------------------

BnaA10.SIP1_2a --------------------------------------------------

BnaCnn_random.SIP2_1c --------------------------------------------------

BnaA07.SIP2_1a --------------------------------------------------

BnaC04.SIP2_1b --------------------------------------------------

BnaC03.SIP2_1a --------------------------------------------------

BnaA09.SIP2_1b --------------------------------------------------

BnaAnn_random.TIP1_1b -------------------------------------------MIFVFAG 7

BnaAnn_random.TIP1_1a -------------------------------------------MIFVFAG 7

BnaCnn_random.TIP1_1a -------------------------------------------MIFVFAG 7

BnaC07.TIP1_2b ---------------------------THPSALRAALAEFISTLIFVFAG 38

BnaA06.TIP1_2b ---------------------------THPSALRAALAEFISTLIFVFAG 38

BnaC02.TIP1_2a ---------------------------THPSALRAALAEFISTLIFVFAG 38

BnaA02.TIP1_2a ---------------------------THPSALRAALAEFISTLIFVFAG 38

BnaCnn_random.TIP1_3a ---------------------------SGRDAIRAAFAEFFSMVIFVFAG 37

BnaA09.TIP1_3a ---------------------------SGRDAIRAAFAEFFSMVIFVFAG 37

BnaCnn_random.TIP3_1c ---------------------------THPDSIRATLAEFLSTFVFVFAA 40

BnaA02.TIP3_1a ---------------------------THPDSIRATLAEFLSTFVFVFAA 40

BnaC06.TIP3_1a ---------------------------THPDSIRATLAEFLSTFVFVFAA 40

BnaA07.TIP3_1b ---------------------------THPDSIRATLAEFLSTFVFVFAA 40

BnaC06.TIP3_1b ---------------------------THPDSIRATLAEFLSTFVFVFAA 40

BnaA07.TIP3_1c ---------------------------THPDSIRATLAEFLSTFVFVFAA 40

BnaC08.TIP3_2b ---------------------------SHPDSIRATLAEFLSTFVFVFAG 40

BnaA09.TIP3_2b ---------------------------SHPDSIRATLAEFVSTFVFVFAG 40

BnaC05.TIP3_2a ---------------------------THPDSIRATLAEFLSTFVFVFAG 40

BnaA06.TIP3_2a ---------------------------THPDSIRATLAEFLSTFVFVFAG 40

BnaC01_random.TIP2_1d ---------------------------FSLASLRAYLAEFISTLLFVFAG 35

BnaA01.TIP2_1a ---------------------------FSLASLRAYLAEFISTLLFVFAG 35

BnaC05.TIP2_1b ---------------------------FSLASLRAYLAEFISTLLFVFAG 35

BnaA05.TIP2_1c ---------------------------FSLASLRAYLAEFISTLLFVFAG 35

BnaC03.TIP2_1a ---------------------------FSLASLKAYLAEFISTLLFVFAG 35

BnaA03.TIP2_1b ---------------------------FSLASLKAYLAEFISTLLFVFAG 35

BnaC06.TIP2_1c ---------------------------FKLVSLKAYLAEFISTLLFVFAG 34

BnaC01_random.TIP2_2a ---------------------------FSVASLKAYLSEFIATLLFVFAG 35

BnaA01_random.TIP2_2a ---------------------------FSVASLKAYLSEFIATLLFVFAG 35

BnaC02_random.TIP2_3b ---------------------------FSVASLKAYLSEFIATLIFVFAG 35

BnaA02.TIP2_3a ---------------------------FT--------------------- 14

BnaA06_random.TIP2_3b ---------------------------FSVASLKAYLSEFIATLIFVFAG 35

BnaC07.TIP2_3a ---------------------------FSVASLKAYLSEFIATLIFVFAG 35

BnaC04.TIP4_1a ---------------------------AQPECIKALIVEFITTFFFVFAG 35

BnaCnn_random.TIP5_1a ---------------------------VSMNALRCYVSEFISTFFFVLAA 39

BnaA06.TIP5_1a ---------------------------VSMNALRCYVSEFISTFFFVLAA 39

BnaCnn_random.PIP2_1b ---------------------------TKWSFYRAVIAEFVATLLFLYIT 55

BnaA09.PIP2_1a ---------------------------TKWSFYRAVIAEFVATLLFLYIT 55

BnaC06.PIP2_1a ---------------------------TKWSFYRAVIAEFVATLLFLYIT 55

BnaC04.PIP2_2/2_3b ---------------------------TKWSLYRAVIAEFVATLLFLYVT 53

BnaA05.PIP2_2/2_3c ---------------------------TKWSLYRAVIAEFVATLLFLYVT 53

BnaC04.PIP2_2/2_3a ---------------------------TKWSLYRAVIAEFVATLLFLYVT 53

BnaA03.PIP2_2/2_3a ---------------------------TKWSLYRAVIAEFVATLLFLYVT 53

BnaA03.PIP2_2/2_3b ---------------------------TRWSLYRAVIAEFVATLLFLYVT 53

BnaC03.PIP2_4a ---------------------------GKWSLYR---------------- 39

BnaA03.PIP2_4b ---------------------------GKWSLYR---------------- 39

BnaA10.PIP2_4c ---------------------------GKWSLYR---------------- 39

BnaC09_random.PIP2_4b ---------------------------GKWSLYR---------------- 39

BnaA02.PIP2_4a ---------------------------GKWSLYR---------------- 37

BnaC08.PIP2_5b ---------------------------GKWSFYRALIAEFIATLLFLYVT 54

BnaA09.PIP2_5b ---------------------------GKWSFYRALIAEFIATLLFLYVT 54

BnaC06.PIP2_5a ---------------------------GRWSFYRALIAEFIATLLFLYVT 54

BnaA07.PIP2_5a ---------------------------GRWSFYRALIAEFIATLLFLYVT 54

BnaC03.PIP2_6a ---------------------------GKWSFYRAVIAEFIATLLFLYIT 53

BnaA03.PIP2_6a ---------------------------GKWSFYRAVIAEFIATLLFLYVT 53

BnaAnn_random.PIP2_7b ---------------------------KSWSFYRALIAEFIATLLFLYVT 55

BnaC01.PIP2_7a ---------------------------KSWSFYRALIAEFIATLLFLYVT 55

BnaC03.PIP2_7b ---------------------------KSWSFYRALIAEFIATLLFLYVT 55

BnaA08.PIP2_7a ---------------------------KSWSFYRALIAEFIATLLFLYVT 55

BnaC07.PIP2_7c ------------------------------------FCYFVATLLFLYVT 28

BnaC07.PIP1_5a ---------------------------KSWSFYRAGIAEFIAAFLFLYVT 69

BnaA03.PIP1_5a ---------------------------KSWSFYRAGIAEFIATFLFLYVT 69

BnaCnn_random.PIP1_3b ---------------------------SSWSFWRAGIAEFIATFLFLYIT 68

BnaC05.PIP1_3a ---------------------------SSWSFWRAGIAEFIATFLFLYIT 68

BnaA10.PIP1_3a ---------------------------SSWSFWRAGIAEFIATFLFLYIT 68

BnaCnn_random.PIP1_4b ---------------------------SSWSFWRAGIAEFIATFLFLYIT 68

BnaA09_random.PIP1_4b ---------------------------SSWSFWRAGIAEFIATFLFLYIT 68

BnaC03.PIP1_4a ---------------------------SSWSFWRAGIAEFIATFLFLYIT 68

BnaA03.PIP1_4a ---------------------------SSWSFWRAGIAEFIATFLFLYIT 68

BnaA09.PIP1_1b ---------------------------SSWSFWRAGIAEFIATFLFLYIT 68

BnaC08.PIP1_1a ---------------------------SSWSFWRAGIAEFIATFLFLYIT 68

BnaA04.PIP1_1a --------------------------------------------------

BnaAnn_random.PIP1_1c --------------------------------------------------

BnaC04.PIP1_2b ---------------------------ASWSFWRAGIAEFIATFLFLYIT 68

BnaA05.PIP1_2c ---------------------------ASWSFWRAGIAEFIATFLFLYIT 68

BnaC03.PIP1_2a ---------------------------ASWSFWRAGIAEFIATFLFLYIT 68

BnaA03.PIP1_2a ---------------------------ASWSFWRAGIAEFIATFLFLYIT 68

BnaC04.PIP1_2c ---------------------------ASWSFWRAGIAEFIATFLFLYIT 68

BnaA04.PIP1_2b ---------------------------ASWSFWRAGIAEFIATFLFLYIT 68

BnaC07.NIP1_2b -------------------------LSFSVPFLQKLMAEILGTYFLIFAG 72

BnaA03.NIP1_2b -------------------------LSFSVPFLQKLMAEILGTYFLIFAG 72

BnaC01.NIP1_2a -------------------------LSISVPFLQKLMAEILGTYFLIFAG 71

BnaA01.NIP1_2a -------------------------LSISVPFLQKLMAEILGTYFLIFAG 71

BnaC04.NIP2_1b -------------------------PLLSVHFLQKLIAELVGTYYLIFAG 62

BnaA05.NIP2_1b -------------------------PLLSVHFMQKLIAELVGTYYLIFAG 62

BnaC04.NIP2_1a -------------------------PLLSVHFLQKLIAELVGTYYLIFAG 62

BnaA05.NIP2_1a -------------------------PLLSVHFLQKLIAELVGTYYLIFAG 62

BnaA04.NIP4_1a -------------------------CLTQK-----LIAEMIGTYFLIFAG 59

BnaC04.NIP4_1a -------------------------CLTQK-----LIAEMIGTYFLIFAG 59

BnaC04.NIP4_1b -------------------------CLTQK-----LIAELIGTYFIIFSG 59

BnaC06_random.NIP4_2a -------------------------CLTQKPPQYVLIAELIGTYFIIFSG 64

BnaA04_random.NIP4_1b ---------------------------------------MIGTYFVIFSG 11

BnaC08.NIP3_1c -------------------------PLVSVSFVQKLIGEFVGTFSLIFAG 59

BnaA08.NIP3_1c -----------------------------------LIGEFVGTFSLIFAG 32

BnaC05.NIP3_1b -------------------------SLICISFVQKLIGEFVGTFSLVFAG 73

BnaC05.NIP3_1a -------------------------SLICISFVQKLIGEFVGTFSLVFAG 73

BnaA05.NIP3_1b -------------------------SLICISFVQKLIGEFVGTFSLVFAG 73

BnaA05.NIP3_1a -------------------------PPIFISFVQKLFGEFVGTFSLVFAG 56

BnaC03.NIP5_1b CKCLPVMGNPWGQHD----TCFTDFPTPGVSLTRKLGAEFVGTFILIFTA 91

BnaA03.NIP5_1b CKCLPVMGNRWGQHD----TCFADFPSPGVSLTRKLGAEFVGTFILIFTA 91

BnaC02.NIP5_1a CKCLPVMGHAWGQPD----TCFTNFPSPVVSLTRKLGAEFVGTFILIFTA 91

BnaA02.NIP5_1a CKCLPVMGHTWGQPD----TCFTDFPSPVVSLTRKLGAEFVGTFILIFTA 91

BnaA07.NIP5_1c CKCLPMMGNPWGQHD----TCFTDIPSPGVSLTRKLGAEFVGTFILIFTA 91

BnaC06_random.NIP5_1c CKCLPMMGNPWGQHD----TCFTDFPSPGASLTRKLGAEFVGTFILIFTA 91

BnaA02_random.NIP6_1c CKCFSVD-NEWALEDGRLPPVSCALPPPNISLYRKLGAEFVGTLILIFAG 96

BnaA02.NIP6_1a CKCFSVD-NEWALEDGRLPPVSCALPPPNVSLYRKLGAEFVGTLILIFAG 96

BnaC06.NIP6_1a CKCFNVD-NEWALEDGRLTPVSCALPPPNVSLYRKLGAEFVGTLILIFAG 96

BnaA07.NIP6_1b CKCFNVD-NEWALEDGRLPPVSCALPPPNVSLYRKLGAEFVGTLILIFAG 96

BnaC05.NIP7_1a --------------------------ELDLNPLRIVIAELVGTFILMFSV 61

BnaA05.NIP7_1a --------------------------ELDLNPLRIVIAELVGTFILMFSV 61

BnaA05_random.SIP1_1b -----------------------------MGVVKSAIGDMLMTFSWVVLS 21

BnaC05.SIP1_1b -----------------------------MGVVKSAIGDMLMTFSWVVLS 21

BnaC01.SIP1_1a -----------------------------MGVIKSAIGDMLMTFSWVVLS 21

BnaA01.SIP1_1a -----------------------------MGVVKSATGDMLMTFSWVVLS 21

BnaC09_random.SIP1_2a -----------------------------MNAVRSALGDMVITFFWVILS 21

BnaA10.SIP1_2a -----------------------------MNAVRSALGDMVITFFWVILS 21

BnaCnn_random.SIP2_1c -----------------------------MGRISLVVSDLVLSFMWIWAG 21

BnaA07.SIP2_1a -----------------------------MGRISLVVSDLVLSFMWIWAG 21

BnaC04.SIP2_1b -----------------------------MGRIGIVVSDLVLSFMWTWAG 21

BnaC03.SIP2_1a -----------------------------MGRIGIVVSDLVLSFMWTWAG 21

BnaA09.SIP2_1b -----------------------------MSRISIVVSDLVLSFMWIWSG 21

BnaAnn_random.TIP1_1b SGSGMAFNKLTEN-----GATTPAGLVAASLAHAFGLFVAVSVGANISGG 52

BnaAnn_random.TIP1_1a SGSGMAFNKLTEN-----GATTPAGLVAASLAHAFGLFVAVSVGANISGG 52

BnaCnn_random.TIP1_1a SGSGMAFNKLTEN-----GGTTPSGLVAAALAHAFGLFVAVSVGANISGG 52

BnaC07.TIP1_2b SGSGIAFNKLTDN-----GATTPSGLVAAALAHAFGLFVAVSVGANISGG 83

BnaA06.TIP1_2b SGSGIAFNKLTDN-----GATTPSGLVAAALAHAFGLFVAVSVGANISGG 83

BnaC02.TIP1_2a SGSGIAFNKLTDN-----GATTPSGLVAAALAHAFGLFVAVSVGANISGG 83

BnaA02.TIP1_2a SGSGIAFNKLTDN-----GATTPSGLVAAALAHAFGLFVAVSVGANISGG 83

BnaCnn_random.TIP1_3a QGSGMAYGKLTGD-----GPATPSGLVAASLSHAFALFVAVSVGANVSGG 82

BnaA09.TIP1_3a QGSGMAYGKLTGD-----GPATPSGLVAASLSHAFALFVAVSVGANVSGG 82

BnaCnn_random.TIP3_1c EGSILSLDKLYWDRAAHAGTDTPGGLVLVALAHAFALFAAVSAAANVSGG 90

BnaA02.TIP3_1a EGSILSLDKLYWDHAAHAGTNTPGGLVLAALAHAFALFAAVSAAANVSGG 90

BnaC06.TIP3_1a EGSILSLDKLYWSHAAHAGTNTPGGLVLVALAHAFALFAAVSAAINVSGG 90

BnaA07.TIP3_1b EGSILSLDKLYWSHAAHAGTNTPGGLVLVALAHAFALFAAVSAAINVSGG 90

BnaC06.TIP3_1b EGSILSLDKLYWDHAAHVGTNTPGGLVLVALAHAFALFAAVSAAINVSGG 90

BnaA07.TIP3_1c EGSILSLDKLYWDHAAHVGTNTPGGLVLVALAHAFALFAAVSAAINVSGG 90

BnaC08.TIP3_2b EGSILALDKLYWDTAAHTGTDTPGGLVLVALAHALALFAAISAAINVSGG 90

BnaA09.TIP3_2b EGSILALDKLYWDTAAHTGTDTPGGLVLVALAHALALFAAISAAINVSGG 90

BnaC05.TIP3_2a EGSILSLDKLYWDTEAHTGTDTPGGLLLVALAHALALFAAVSAAINVSGG 90

BnaA06.TIP3_2a EGSILSLDTLYWDTAAHTGIDTPGGLLLVALAHALALFAAVSAAINVSGG 90

BnaC01_random.TIP2_1d VGSAIAYAKLTSD-----AALDTPGLVAIAVCHGFALFVAVAIGANISGG 80

BnaA01.TIP2_1a VGSAIAYAKLTSD-----AALDTPGLVAIAVCHGFALFVAVAIGANISGG 80

BnaC05.TIP2_1b VGSAIAYAKLTSD-----AALDTPGLVAIAVCHGFALFVAVAIGANISGG 80

BnaA05.TIP2_1c VGSAIAYAKLTSD-----AALDTPGLVAIAVCHGFALFVAVAVGANISGG 80

BnaC03.TIP2_1a VGSAIAYGKLTSD-----AALDTSGLVAIAVCHGFALFVAVAIGANISGG 80

BnaA03.TIP2_1b VGSAIAYGKLTSD-----AALDTSGLVAIAVCHGFALFVAVAIGANISGG 80

BnaC06.TIP2_1c VGSAIAFGKLTNN-----AALDAPGLVAIAVCHGFALFVAVSIAANHSGG 79

BnaC01_random.TIP2_2a VGSAIAFGKLTSN-----AALDPAGLVAVAVAHAFALFVGVSIAANISGG 80

BnaA01_random.TIP2_2a VGSAIAFGKLTSN-----AALDPAGLVAVAVAHAFALFVGVSIAANISGG 80

BnaC02_random.TIP2_3b VGSAIAFGKLTSD-----AALDPAGLVAIAVAHAFALFVGVSIAANISGG 80

BnaA02.TIP2_3a ------------------------GLVAIAVAHAFALFVGVSIAANISGG 40

BnaA06_random.TIP2_3b VGSAIAFGKITSD-----AALDPAGLVAIAVAHAFALFVGVSVAANISGG 80

BnaC07.TIP2_3a VGSAIAFGKITSD-----AALDPAGLVAIAVAHAFALFVGVSVAANISGG 80

BnaC04.TIP4_1a VGSAMATDSLVGN--------TLVGLLAVAVAHALVVAVMISAG-HISGG 76

BnaCnn_random.TIP5_1a VGSVMASRKLTAG-----DVTGPFSVLIPAIANAFALSSSVYISWNVSGG 84

BnaA06.TIP5_1a VGSVMASRKLTAG-----DVTGPFSVLLPAIANAFALSSSVYISWNVSGG 84

BnaCnn_random.PIP2_1b VLTVIGYKIQTDSTAGG-VDCGGVGILGIAWAFGGMIFILVYCTAGISGG 104

BnaA09.PIP2_1a VLTVIGYKIQTDSTAGG-VDCGGVGILGIAWAFGGMIFILVYCTAGISGG 104

BnaC06.PIP2_1a VLTVICYKIQTDSTAGG-VDCGGVGILGIAWAFGGMIFILVYCTAGISGG 104

BnaC04.PIP2_2/2_3b VLTVIGYKISSDTKAGG-DECGGVGILGISWAFGGMIFILVYCTAGISGG 102

BnaA05.PIP2_2/2_3c VLTVIGYKISSDTKAGG-DECGGVGILGISWAFGGMIFILVYCTAGISGG 102

BnaC04.PIP2_2/2_3a VLTVIGYKISSDTKAGG-DECGGVGILGISWAFGGMIFILVYCTAGISGG 102

BnaA03.PIP2_2/2_3a VLTVIGYKISSDTKAGG-DDCGGVGILGISWAFGGMIFILVYCTAGISGG 102

BnaA03.PIP2_2/2_3b VLTVMGYKISSDTAAGG-VECGGVGILGIAWAFGGMIFILVYCTAGISGG 102

BnaC03.PIP2_4a ---------ATDANAGG-VDCGGVGILGIAWAFGGMIFVLVYCTAGISGG 79

BnaA03.PIP2_4b ---------ATDANAGG-VDCGGVGILGIAWAFGGMIFVLVYCTAGISGG 79

BnaA10.PIP2_4c ---------ATDSNAGG-VDCGGVGILGIAWAFGGMIFVLVYCTAGVSGG 79

BnaC09_random.PIP2_4b ---------ATDANAGG-VDCGGVGILGIAWAFGGMIFVLVYCTAGVSGG 79

BnaA02.PIP2_4a ---------ATDASAGG-ADCGGVGILGIAWAFGGMIFVLVYCTAGISGG 77

BnaC08.PIP2_5b VMTVIGYKSQTDPALNP-DQCAGVGVLGIAWAFGGMIFILVYCTAGISGG 103

BnaA09.PIP2_5b VMTVIGYKSQTDPALNP-DQCAGVGVLGIAWAFGGMIFILVYCTAGISGG 103

BnaC06.PIP2_5a VMTVIGYKSQTDPTLYP-DQCAGVGVLGIAWAFGGMIFILVYCTAGISGG 103

BnaA07.PIP2_5a VMTVIGYKSQTDPTLNP-DQCAGVGVLGIAWAFGGMIFILVYCTAGISGG 103

BnaC03.PIP2_6a VLTVIGFKSQTDLQTGG-GACASVGLLGISWAFGGMIFILVYCTAGISGG 102

BnaA03.PIP2_6a VLTVIGFKSQTDLQTGG-GACASVGLLGISWAFGGMIFILVYCTAGISGG 102

BnaAnn_random.PIP2_7b VATVIGHKKQTGP-------CDGVGLLGIAWAFGGMIFVLVYCTAGISGG 98

BnaC01.PIP2_7a VATVIGHKKQTGP-------CDGVGLLGIAWAFGGMIFVLVYCTAGISGG 98

BnaC03.PIP2_7b VATVIGHKKQTGP-------CDGVGLLGIAWAFGGMIFVLVYCTAGVSGG 98

BnaA08.PIP2_7a VATVIGHKKQTGP-------CDGVGLLGIAWAFGGMIFVLVYCTAGISGG 98

BnaC07.PIP2_7c VATVIGHKKQTGP-------CDGVGLLGIAWAFGGMIFVLVYCTAGISGG 71

BnaC07.PIP1_5a VLTVLGVKRAPNM-------CASVGIQGIAWAFGGMIFALVYCTAGISGG 112

BnaA03.PIP1_5a VLTVMGVKRAPNM-------CASVGIQGIAWAFGGMIFALVYCTAGISGG 112

BnaCnn_random.PIP1_3b VLTNQRVMGAPNM-------CASVGIQGIAWAFGGMIFALVYCTAGISGG 111

BnaC05.PIP1_3a VLTVMGVKRAPNM-------CASVGIQGIAWAFGGMIFALVYCTAGISGG 111

BnaA10.PIP1_3a VLTVMGVKRAPNM-------CASVGIQGIAWAFGGMIFALVYCTAGISGG 111

BnaCnn_random.PIP1_4b VLTVMGVKRAPNM-------CASVGIQGIAWAFGGMIFALVYCTAGISGG 111

BnaA09_random.PIP1_4b VLTVMGVKRAPNM-------CASVGIQGIAWAFGGMIFALVYCTAGISGG 111

BnaC03.PIP1_4a VLTVMGVKRAPNM-------CASVGIQGIAWAFGGMIFALVYCTAGISGG 111

BnaA03.PIP1_4a VLTVMGVKRAPNM-------CASVGIQGIAWAFGGMIFALVYCTAGISGG 111

BnaA09.PIP1_1b VLTVMGVKRSPNM-------CSSVGIQGIAWAFGGMIFALVYCTAGISGG 111

BnaC08.PIP1_1a VLTVMGVKRSPNM-------CASVGIQGIAWAFGGMIFALVYCTAGISGG 111

BnaA04.PIP1_1a --TVMGVKRSPNM-------CASVGIQGIAWAFGGMIFALVYCTAGISGG 50

BnaAnn_random.PIP1_1c ------------M-------CASVGIQGIAWAFGGMIFALVYCTAGISGG 31

BnaC04.PIP1_2b VLTVMGVKRSPNM-------CASVGIQGIAWAFGGMIFALVYCTAGISGG 111

BnaA05.PIP1_2c VLTVMGVKRSPNM-------CASVGIQGIAWAFGGMIFALVYCTAGISGG 111

BnaC03.PIP1_2a VLTVMGVKRSPSM-------CASVGIQGIAWAFGGMIFALVYCTAGVSGG 111

BnaA03.PIP1_2a VLTVMGVKRSPSM-------CASVGIQGIAWAFGGMIFALVYCTAGISGG 111

BnaC04.PIP1_2c VLTVMGVKRAPNM-------CASVGIQGIAWAFGGMIFALVYCTAGISGG 111

BnaA04.PIP1_2b VLTVMGVKRAPNM-------CASVGIQGIAWAFGGMIFALVYCTAGISGG 111

BnaC07.NIP1_2b CASVAVNAQHDKA----------VTLPGIAIVWGLTVMVLVYSLGHISGA 112

BnaA03.NIP1_2b CASVAVNAQHDKA----------VTLPGIAIVWGLTVMVLVYSLGHISGA 112

BnaC01.NIP1_2a CASVAVNAQHDKT----------VTHPGIAIVWGLTVMVLVYSLGHISGA 111

BnaA01.NIP1_2a CASVAVNAQHDKA----------VTLPGIAIVWGLTVMVLVYSLGHISGA 111

BnaC04.NIP2_1b CAAIAVNAQHNNV----------VTLVGIAVVWGLVIMVLVYSLGHIS-A 101

BnaA05.NIP2_1b CAAIAVNAQHNNV----------VTLVGIAVVWGLVVMVLVYSLGHIS-A 101

BnaC04.NIP2_1a CAAIAVNAQHNNV----------VTLVGIAVVWGLVVMVLVYSLGHIS-A 101

BnaA05.NIP2_1a CAAIAVNAQHNNV----------VTLVGIAVVWGLVVMVLVYSLGHIS-A 101

BnaA04.NIP4_1a CGVVVVNVLYGGT----------VTFPGICVTWGLIVMVMIYSTGHISGA 99

BnaC04.NIP4_1a CGVVVVNVLYGGT----------VTFPGICVTWGLIVMVMIYSTGHISGA 99

BnaC04.NIP4_1b CGVVVVNVLYGGK----------VTFPGICVTWGLIVMVMIYSVGHISGA 99

BnaC06_random.NIP4_2a CGVVVVNVLYGGK----------VTFPGICVTWGLIVMVMIYSIGHISGA 104

BnaA04_random.NIP4_1b CGVVVVNVLYGGT----------VTFPGVCVTWGLIVMVMIYSTGHISGA 51

BnaC08.NIP3_1c CAAIVVNDTYGKA----------VTLPGIALVWGLTVMVMIYSIGHVSGA 99

BnaA08.NIP3_1c CAAIVVNDTYGKA----------VTLPGIALVWGLTVMVMIYSIGHVSGA 72

BnaC05.NIP3_1b CSATVVNDTYGEL----------VTLPGIALAWGLTVMVMSYSIGHVSGA 113

BnaC05.NIP3_1a CSATVVNDTYGEL----------VTLPGIALAWGLTVMVMSYSIGHVSGA 113

BnaA05.NIP3_1b CSATVVNDTYGEL----------VTLPGIALAWGLTVMVMTYSIGHVSGA 113

BnaA05.NIP3_1a CSAIVVNDTYGKP----------VTLPGIALTWGLTVMVMTYSIGHISGA 96

BnaC03.NIP5_1b TAGPIVNQKYDGA----------ETLIGNAACAGLAVMIIILSTGHISGA 131

BnaA03.NIP5_1b TAGPIVNQKYDGA----------ETLIGNAACAGLAVMIIILSTGHISGA 131

BnaC02.NIP5_1a TAGPIVNQKYDGA----------ETLIGNAACAGLAVMIIILSTGHISGA 131

BnaA02.NIP5_1a TAGPIVNQKYDGA----------ETLIGNAACAGLAVMIIILSTGHISGA 131

BnaA07.NIP5_1c TAGPIVNQKYDGA----------ETLIGNAACAGLAVMIIILSTGHISGA 131

BnaC06_random.NIP5_1c TAGPIVNQKYDGA----------ETLIGNAACAGLAVMIIILSTGHISGA 131

BnaA02_random.NIP6_1c TATAIVNQKTDGA----------VTLIGCAASAGLAVMIVILSTGHISGA 136

BnaA02.NIP6_1a TATAIVNQKTDGA----------VTLIGCAASAGLAVMIVILSTGHISGA 136

BnaC06.NIP6_1a TATAIVNQKTDGA----------VTLIGCAASAGLAVMIVILSTGHISGA 136

BnaA07.NIP6_1b AATAIVNQKTDGA----------VTLIGCAASAGLAVMIVILSTGHISGA 136

BnaC05.NIP7_1a CGVISSTQLSGGH----------VGLLEYAATAGLSVVVVVYSIGHISGA 101

BnaA05.NIP7_1a CGVISSTQLSGGH----------VGLLEYAATAGLSVVVVVYSIGHISGA 101

BnaA05_random.SIP1_1b ATFGLQTTEIISA--AGFQGIAWAPLAITTFLIFFYVSIFTVV---FGSA 66

BnaC05.SIP1_1b ATFGLQTTEIISA--AGFQGIAWAPLAITTFLIFVYVSIFTVV---FGSA 66

BnaC01.SIP1_1a ATFGLQTTEIISA--AGLHGITWAPLAITTFLIFVYVSLFTVV---FGSA 66

BnaA01.SIP1_1a ATFGLQTTEIISA--AGLHGVTWAPLAITTFLIFVYVSLFTVV---FGSA 66

BnaC09_random.SIP1_2a ATFGLQTAAIVSA--AGFHGITWAPPLITTVVVFFSISVFTVIGNFLGGA 69

BnaA10.SIP1_2a ATFGLQTAAIISA--AGFHGITWAPPLITTVVVFFSISVFTVIGNFLGGA 69

BnaCnn_random.SIP2_1c VLVNILVHGVL-----GFSRKDTTGDIVRYLFSVISMFVFAFLQKLTKGG 66

BnaA07.SIP2_1a VLVNVLVHGVL-----GFSRKDTTGDIVRYLFSVISMFVFAFLQKLTKGG 66

BnaC04.SIP2_1b VLVNILVHGVL-----GFSRKDTTGEIVRYLFSVISMFVFAFLQKLSKGG 66

BnaC03.SIP2_1a VLVNILVHGVL-----GFSRKDTTGEIVRYLFSVISMFVFAFLQKLSKGG 66

BnaA09.SIP2_1b VLVSILVHGVL-----GFSRNVTTGEIVGYTFSVISMFIFAFLQKLTKGG 66

: .

**（P1）**

BnaAnn_random.TIP1_1b HVNPAVTFGAFVGGNIT--LLRGILYWIAQLLGSVVACLLLKFATGGLVV 100

BnaAnn_random.TIP1_1a HVNPAVTFGAFVGGNIT--LLRGILYWIAQLLGSVVACLLLKFATGGLVV 100

BnaCnn_random.TIP1_1a HVNPAVTFGAFVGGNIT--LLRGILYWIAQLLGSVVACLLLKFATGGLVV 100

BnaC07.TIP1_2b HVNPAVTFGAFVGGNIT--LLRGILYWIAQLLGSVVACLLLKFATGGLAV 131

BnaA06.TIP1_2b HVNPAVTFGAFVGGNIT--LLRGILYWIAQLLGSVVACLLLKFATGGLAV 131

BnaC02.TIP1_2a HVNPAVTFGAFLGGNIT--LLRGLLYWIAQLLGSVVACFLLQFATGGLAV 131

BnaA02.TIP1_2a HVNPAVTFGAFLGGNIT--LLRGLLYWIAQLLGSVVACFLLQFATGGLAV 131

BnaCnn_random.TIP1_3a HVNPAVTFGAFIGGNIT--LLRAILYWIAQLLGAVVACLLLKVSTGGMET 130

BnaA09.TIP1_3a HVNPAVTFGAFIGGNIT--LLRAILYWIAQLLGAVVACLLLKVSTGGMET 130

BnaCnn_random.TIP3_1c HVNPAVTFGALIGGRLS--AIRAIYYWIAQLLGAILACLLLRLTTNGMRP 138

BnaA02.TIP3_1a HANPAVTFGALIGGRLS--AIRAIYYWIAQLLGAILACLLLRLATNGMRP 138

BnaC06.TIP3_1a HVNPAVTFGALIGGRLS--AIRAIYYWIAQLLGAILACLLLRLSTNGMRP 138

BnaA07.TIP3_1b HVNPAVTFGALIGGRLS--AIRAIYYWIAQLLGAILACLLLRLSTNGMRP 138

BnaC06.TIP3_1b HVNPAVTFGALIGGRIS--AILAIYYWIAQLLGAILACLLLRLATNGMRP 138

BnaA07.TIP3_1c HVNPAVTFGALIGGRIS--AIRAIYYWIAQLLGAILACLLLRLSTNGMRP 138

BnaC08.TIP3_2b HVNPAVTFAALVGGRLS--VIRAIYYWIAQLLGAILACLLLRLATNGSRP 138

BnaA09.TIP3_2b HVNPAVTFAALVGGRLS--VIRAIYYWIAQLLGAILACLLLRLATNGSRP 138

BnaC05.TIP3_2a HVNPAVTFAALIGGRLS--VIRAIYYWVAQLLGAILACLLLRLSTNGKRP 138

BnaA06.TIP3_2a HVNPAVTFAALVGGRLS--VIRAIYYWVAQLLGAILACLLLRLSTNGKRP 138

BnaC01_random.TIP2_1d HVNPAVTFGLALGGQIT--LITGVFYWIAQLLGSTAACFLLKFVTGGLAV 128

BnaA01.TIP2_1a HVNPAVTFGLALGGQIT--LITGVFYWIAQLLGSTAACFLLKFVTGGLAV 128

BnaC05.TIP2_1b HVNPAVTFGLAVGGQIT--LITGVFYWVAQLLGSTAACFLLKYVTGGLAV 128

BnaA05.TIP2_1c HVNPAVTFGLAVGGQIT--LITGVFYWVAQLLGSTAACFLLKYVTGGLAV 128

BnaC03.TIP2_1a HVNPAVTFGLALGGQIT--LITGLFYWIAQLLGSTAACFLLKFVTGGLAV 128

BnaA03.TIP2_1b HVNPAVTFGLALGGQIT--LITGVFYWIAQLLGSTAACFLLKFVTGGLAV 128

BnaC06.TIP2_1c HVNPAVTFGLVLGGKLK--IVTGVCYWVAQLLGSTAACFLLKFVTGGLAI 127

BnaC01_random.TIP2_2a HLNPAVTLGLAVGGNIT--VITSFFYWIAQCLGSIVACLLLVFVTIGESV 128

BnaA01_random.TIP2_2a HLNPAVTLGLAVGGNIT--VITGFFYWIAQCLGSIVACLLLAFVTNGESV 128

BnaC02_random.TIP2_3b HLNPAVTLGLAVGGNIT--LITGFLYWIAQCLGSIVACLLLVYVTNGESV 128

BnaA02.TIP2_3a HLNPAVTLGLAVGGNIT--LITGFLYWIAQCLGSIVACLLLVYVTNGESV 88

BnaA06_random.TIP2_3b HLNPAVTLGLAVGGNIT--LITGFLYWVAQCLGSTVACLLLVFVTNGESV 128

BnaC07.TIP2_3a HLNPAVTLGLAVGGNIT--LITGFLYWVAQCLGSTVACLLLVFVTNGESV 128

BnaC04.TIP4_1a HLNPAVTIGLLFGGHIS--VFRAFLYWIDQLLASSAACFLLSYLTGGMGT 124

BnaCnn_random.TIP5_1a HVNPAVTFGMAVAGRIS--VPTALFYWASQMIASVMACLVLKVTVIEQHV 132

BnaA06.TIP5_1a HVNPAVTFGMAVAGRIS--VPTAMFYWTSQMIASVMACLVLKVTVVEQHV 132

BnaCnn_random.PIP2_1b HINPAVTFGLLLARKVS--LVRAILYMVAQCLGAICGVGFVKAFQSSYYV 152

BnaA09.PIP2_1a HINPAVTFGLLLARKVS--LVRAILYMVAQCLGAICGVGFVKAFQSSYYV 152

BnaC06.PIP2_1a HINPAVTFGLLLARKVS--LVRAILYMVAQCLGAICGVGFVQAFQSSYYV 152

BnaC04.PIP2_2/2_3b HINPAVTFGLFLARKVS--LVRAVLYMVAQCLGAICGVGFVKAFQSAYYV 150

BnaA05.PIP2_2/2_3c HINPAVTFGLFLARKVS--LVRAVLYMVAQCLGAICGVGFVKAFQSAYYV 150

BnaC04.PIP2_2/2_3a HINPAVTFGLFLARKVS--LIRAVLYMVAQCLGAICGVGFVKAFQSSYYV 150

BnaA03.PIP2_2/2_3a HINPAVTFGLFLARKVS--LVRAVLYMVAQCLGAICGVGFVKAFQSAYYV 150

BnaA03.PIP2_2/2_3b HINPAVTFGLFLARKVT--LVRALLYMVAQCLGAICGVGFVKAFQSAYYV 150

BnaC03.PIP2_4a HINPAVTVGLFLARKVS--LVRTVLYIVAQCLGAICGCGLVKAFQSSYYT 127

BnaA03.PIP2_4b HINPAVTVGLFIARKVS--LVRTVLYIVAQCLGAICGCGLVKAFQSSYYN 127

BnaA10.PIP2_4c HINPAVTFGLFLARKVS--LVRTVLYIVAQCLGAICGCGLVKAFQSSYYT 127

BnaC09_random.PIP2_4b HINPAVTFGLFLARKVS--LVRTVLYIVAQCLGAICGCGLVKAFQSSYYT 127

BnaA02.PIP2_4a HINPAVTFGLFLARKVS--FVRTVLYIVAQCLGAICGCGLVKAFQSSYYN 125

BnaC08.PIP2_5b HINPAVTFGLLLARKVT--LLRAVMYMVAQCLGAICGVALVKSFQSSYYT 151

BnaA09.PIP2_5b HINPAVTFGLLLARKVT--LLRAVMYMVAQCLGAICGVALVKSFQSSYYT 151

BnaC06.PIP2_5a HINPAVTFGLFLARKVT--LVRAVMYMVAQCLGAICGVALVKSFQSSYYT 151

BnaA07.PIP2_5a HINPAVTFGLFLARKVT--LVRAVMYMVAQCLGAICGVALVKSFQSSYYT 151

BnaC03.PIP2_6a HINPAVTFGLFLASKVS--LVRAISYIVAQCLGATCGVGLVKVFQKTYYN 150

BnaA03.PIP2_6a HINPAVTFGLFLASKVS--LVRAISYIVAQCLGATCGVGLVKVFQKTYYN 150

BnaAnn_random.PIP2_7b HINPAVTFGLFLARKVS--LVRAVGYMIAQCLGAICGVGFVKAFMKTPYN 146

BnaC01.PIP2_7a HINPAVTFGLFLARKVS--LVRAVGYMIAQCLGAICGVGFVKAFMKTPYN 146

BnaC03.PIP2_7b HINPAVTFGLFLARKVS--LVRAVGYMIAQCLGAICGVGFVKAFMKTPYN 146

BnaA08.PIP2_7a HINPAVTFGLFLARKVS--LVRAVGYMIAQCLGAICGVGFVKAFMKTPYN 146

BnaC07.PIP2_7c HINPAVTFGLFLARKVS--LVRAVGYMIAQCLGAICGVGFVKAFMKTPYN 119

BnaC07.PIP1_5a HINPAVTFGLFLARKLS--LTRTVFYIVMQCLGAICGAGVVKGFQPGPYH 160

BnaA03.PIP1_5a HINPAVTFGLFLARKLS--LTRTVFYIVMQCLGAICGAGVVKGFQPRPYQ 160

BnaCnn_random.PIP1_3b HINPAVTFGLFLARKLS--LTRAVFYMVMQCLGAICGAGVVKGFQPTPYQ 159

BnaC05.PIP1_3a HINPAVTFGLFLARKLS--LTRAVFYMVMQCLGAICGAGVVKGFQPSPYQ 159

BnaA10.PIP1_3a HINPAVTFGLFLARKLS--LTRAVLYIVMQCLGAVCGAGVVKGFQPTPYQ 159

BnaCnn_random.PIP1_4b HINPAVTFGLFLARKLS--LTRAVFYMIMQCLGAICGAGVVKGFQPTPYE 159

BnaA09_random.PIP1_4b HINPAVTFGLFLARKLS--LTRAVFYMIMQCLGAICGAGVVKGFQPTPYQ 159

BnaC03.PIP1_4a HINPAVTFGLFLARKLS--LTRAVFYMIMQCLGAVCGAGVVKGFQPTPYQ 159

BnaA03.PIP1_4a HINPAVTFGLFLARKLS--LTRAVFYMIMQCLGAICGAGVVKGFQPTPYQ 159

BnaA09.PIP1_1b HINPAVTFGLFLARKLS--LTRALYYIVMQCLGAICGAGVVKGFQPNQYQ 159

BnaC08.PIP1_1a HINPAVTFGLFLARKLS--LTRALYYIVMQCLGAICGAGVVKGFQPNQYQ 159

BnaA04.PIP1_1a HINPAVTFGLFLARKLS--LTRALYYIVMQCLGAICGAGVVKGFQPKQYQ 98

BnaAnn_random.PIP1_1c HINPAVTFGLFLARKLS--LTRALYYIVMQCLGAICGAGVVKGFQPKQYQ 79

BnaC04.PIP1_2b HINPAVTFGLFLARKLS--LTRAVYYIVMQCLGAICGAGVVKGFQPKQYQ 159

BnaA05.PIP1_2c HINPAVTFGLFLARKLS--LTRAVYYIVMQCLGAICGAGVVKGFQPKQYQ 159

BnaC03.PIP1_2a HINPAVTFGLFLARKLS--LTRAVYYIVMQCLGAICGAGVVKGFQPKRYQ 159

BnaA03.PIP1_2a HINPAVTFGLFLARKLS--LTRAVYYIVMQCLGAICGAGVVKGFQPKQYQ 159

BnaC04.PIP1_2c HINPAVTFGLFLARKLS--LTRAVYYIVMQCLGAICGAGVVKGFQPKQYQ 159

BnaA04.PIP1_2b HINPAVTFGLFLARKLS--LTRAVYYIVMQCLGAICGAGVVKGFQPKQYQ 159

BnaC07.NIP1_2b HFNPAVTIAFASCGRFP--LKQVPAYVISQVIGSTLAAATLRLLFGLDQN 160

BnaA03.NIP1_2b HFNPAVTIAFASCGRFP--LKQVPAYVISQVIGSTLAAATLRLLFGLDQD 160

BnaC01.NIP1_2a HFNPALTVAFASCGRFP--LKQVPAYVISQVIGSTLAAATLRLLFGLDQD 159

BnaA01.NIP1_2a HFNPAVTIAFASSGRFP--LKQVPAYVISQVIGSTLAAATLRLLFGLDQD 159

BnaC04.NIP2_1b HFNPAVTIALASCKRFP--LYQLPAYLTVQVIGSTLASATLRILFDLNND 149

BnaA05.NIP2_1b HFNPAVTIALASCKRFP--LYQLPAYLIVQVIGSTLASATLRLLFDLNND 149

BnaC04.NIP2_1a HFNPAVTIALASCKRFP--LYQLPAYLTVQVIGSTLASATLRLLFDLNND 149

BnaA05.NIP2_1a HFNPAVTIALASCKSFP--LYQLPAYLIVQVIGSTLASATLRLLFDLNND 149

BnaA04.NIP4_1a HFNPAVTLTFAVFRRFP--WYQVPLYIGAQLTGSLLGSLTLKLMFHVTP- 146

BnaC04.NIP4_1a HFNPAVTLTFAVFRRFP--WYQVPLYIGAQLTGSLLGSLTLKLMFHVTP- 146

BnaC04.NIP4_1b HFNPAVTICFAIFRRFP--WYQVPSYIGAQLAGSLLASLTLRLMFKVTP- 146

BnaC06_random.NIP4_2a HFNPAVTITFAVFRRFP--WYQVPLYIGAQLSGSLLASLILRLMFNVTP- 151

BnaA04_random.NIP4_1b HFNPAVTLTFAIFRRFP--WYQVPLYVGAQLAGSLLASLTLRLMFKVTP- 98

BnaC08.NIP3_1c HFNPAVSIAFASSRKFP--FKQVPGYIAAQLLGSTLAAEALRLVFHLDDN 147

BnaA08.NIP3_1c HFNPAVSIAFASSRKFP--FKQVPGYIAAQLLGSTLAAEALRLVFHLDDN 120

BnaC05.NIP3_1b HFNPAVSIALASSRKFP--FKQVPGYIAAQLLGSTLAAEALRLMFHLNNN 161

BnaC05.NIP3_1a HFNPAVSIALASSRKFP--FKQVPGYIAAQLLGSTLAAEALRLMFHLNNN 161

BnaA05.NIP3_1b HFNPAVSIALASSRKFP--FKQVPGYIAAQLLGSTLAAEALRLMFHLNNN 161

BnaA05.NIP3_1a HFNPAITIALASSRKFP--LKQVPGYIAAQVLGSTLAIESLRLLFNLNNN 144

BnaC03.NIP5_1b HLNPSLTIAFAALRHFP--WAHVPAYIAAQVSASVCASFALKAVFHPFMS 179

BnaA03.NIP5_1b HLNPSLTIAFAALRHFP--WAHVPAYIAAQVSASVCASFALKAVFHPFMS 179

BnaC02.NIP5_1a HLNPSLTIAFAALRHFP--WAHVPAYIAAQVSASICASFALKAVFHPFMS 179

BnaA02.NIP5_1a HLNPSLTIAFAALRHFP--WAHVPAYIAAQVSASICASFALKAVFHPFMS 179

BnaA07.NIP5_1c HLNPSMTIAFAALRHFP--WAQVPAYIAAQVSASICASFALKAVFHPFMS 179

BnaC06_random.NIP5_1c HLNPSMTIAFAALRHFP--WAQVPAYIAAQVSASICASFALKAVFHPFMS 179

BnaA02_random.NIP6_1c HLNPAVTISFAALKHFP--WKHVPVYIGAQVLASVCAAFALKAVFEPTMS 184

BnaA02.NIP6_1a HLNPAVTISFAALKHFP--WKHVPVYIGAQVLASVCAAFALKAVFEPTMS 184

BnaC06.NIP6_1a HLNPAITIAFAALKHFP--WKHVPVYIGAQVMASLCAAFALKAVFEPTMS 184

BnaA07.NIP6_1b HLNPAITIAFAALKHFP--WKHVPVYIGAQVMASLCAAFALKAVFEPTMS 184

BnaC05.NIP7_1a HLNPSITIAFALFGGFP--WSQVPLYIMAQTLGATAATLAGVSVYGVNPD 149

BnaA05.NIP7_1a HLNPSITIAFALFGGFP--WSQVPLYIMAQTLGATAATLAGVSVYGVNPD 149

BnaA05_random.SIP1_1b SFNPTGNAAFYAAGVPGDTLFTLAIRLPAQAAGAAGGALAIMEFIPEKYK 116

BnaC05.SIP1_1b SFNPTGNAAFYAAGVPGDTLFTLAIRLPAQAAGAAGGALAIMEFIPEKYK 116

BnaC01.SIP1_1a SFNPTGNAAFYAAGIPGDTLFTLAIRLPAQAAGAAGGALAIMEFIPEKYK 116

BnaA01.SIP1_1a SFNPTGNAAFYAAGIPGDTLFTLAIRLPAQAAGAAGGALAIMEFIPEKYK 116

BnaC09_random.SIP1_2a SFNPCGNAAFYTAGVSTDSLFSLAIRSPAQALGAAAGAITIMEMIPEKYK 119

BnaA10.SIP1_2a SFNPCGNAAFYTAGVSTDSLFSLAIRSPAQALGAAAGAITIMEMIPEKYK 119

BnaCnn_random.SIP2_1c LYNPLTALASGVSGGFSSFIFSVAVRIPVEVLGSILAVKHIIHVFPEIGK 116

BnaA07.SIP2_1a LYNPLTALASGVSGGFSSFIFSVVVRIPVEVLGSILAVKHIIHVFPEIGK 116

BnaC04.SIP2_1b LYNPLTALAAGVTGGFSNFIFTVLVRIPVEVLGSILGVKHIIHVFPEIGK 116

BnaC03.SIP2_1a LYNPLTALAAGVTGGFSNFIFTVLVRIPVEVLGSILGVKHIIHVFPEIGK 116

BnaA09.SIP2_1b HYNPVAALAS---GGFGSFIFTIMVRVPAEVIGSILAVKHIIHVFPEIGK 113

** : .: .

BnaAnn_random.TIP1_1b -------PAFGLSAGVGVSNALVFEIVMTFGLVYTVYATAVDPKNGS--- 140

BnaAnn_random.TIP1_1a -------PAFGLSAGVGVSNALVFEIVMTFGLVYTVYATAVDPKNGS--- 140

BnaCnn_random.TIP1_1a -------PAFGLSAGVGVSNALVFEIVMTFGLVYTVYATAVDPKNGS--- 140

BnaC07.TIP1_2b -------PAFGLSAGVESLNGFVFEIVMTFGLVYTVYATAVDPKNGS--- 171

BnaA06.TIP1_2b -------PAFGLSAGVESLNGFVFEIVMTFGLVYTVYATAVDPKNGS--- 171

BnaC02.TIP1_2a -------PAFGLSAGVGTLNGLVFEIVMTFGLVYTVYATAIDPKNGS--- 171

BnaA02.TIP1_2a -------PAFGLSAGVGTLNGLVFEIVMTFGLVYTVYATAIDPKNGS--- 171

BnaCnn_random.TIP1_3a -------AAFSLSHGVTPWNAVVFEIVMTFGLVYTVYATAVDPKKGD--- 170

BnaA09.TIP1_3a -------AAFSLSHGVTPWNAVVFEIVMTFGLVYTVYATAVDPKKGD--- 170

BnaCnn_random.TIP3_1c -------VGFRLASGVGAGNGLVLEIILTFGLVYVVYSTMIDPKRGS--- 178

BnaA02.TIP3_1a -------VGFRLASGVGAVNGLVLEIILTFGLVYVVYSTMIDPKRGS--- 178

BnaC06.TIP3_1a -------VGFRVASGVGAVNGLILEIILTFGLVYVVYSTLIDPKRGS--- 178

BnaA07.TIP3_1b -------VGFRVASGVGAVNGLILEIILTFGLVYVVYSTLIDPKRGS--- 178

BnaC06.TIP3_1b -------VGFSLASGVKAHNGLVLEIILTFGLVYVVYSTLIDPKRGS--- 178

BnaA07.TIP3_1c -------VGFSLASGVKAHNGLVLEIILTFGLVYVVYSTLIDPKRGS--- 178

BnaC08.TIP3_2b -------IGFHVASGVSELHGLLMEIILTFALVYVFYSTVIDPKRGS--- 178

BnaA09.TIP3_2b -------IGFHVASGVSELHGLLMEIILTFALVYVFYSTVIDPKRGS--- 178

BnaC05.TIP3_2a -------IGFHVASGVSELHGLLMEIILTFALVYIFYSTVIDPKRGS--- 178

BnaA06.TIP3_2a -------IGFHVASGVSELHGLLMEIILTFALVYVFYSTVIDPKRGS--- 178

BnaC01_random.TIP2_1d -------PTHSVAAGVGAIEGVVMEIIITFALVYTVYATAADPKKGS--- 168

BnaA01.TIP2_1a -------PTHSVAAGVGAIEGVVMEIIITFALVYTVYATAADPKKGS--- 168

BnaC05.TIP2_1b -------PTHSVAAGVGAIEGVVMEIIITFALVYTVYATAADPKKGS--- 168

BnaA05.TIP2_1c -------PTHSVAAGVGAIEGVVMEIIITFALVYTVYATAADPKKGS--- 168

BnaC03.TIP2_1a -------PTHSVAAGLGAIEGVVMEIIITFALVYTVYATAADPKKGS--- 168

BnaA03.TIP2_1b -------PTHSVAAGLGAIEGVVMEIIITFALVYTVYATAADPKKGS--- 168

BnaC06.TIP2_1c -------PIHSVAAGVGSTEGVVMEIIITFALVYTVYATAVDPKNGT--- 167

BnaC01_random.TIP2_2a -------PTHGVAAGLGAVEGIVMEIVVTFALVYTVYATAADPKKGS--- 168

BnaA01_random.TIP2_2a -------PTHGVAAGLGAVEGIVMEIVVTFALVYTVYATAADPKKGS--- 168

BnaC02_random.TIP2_3b -------PTHGVGAGLGALEGIVMEIVVTFALVYTVYATAADPKKGS--- 168

BnaA02.TIP2_3a -------PTHGVGAGLGALEGIVMEIVVTFALVYTVYATAADPKKGS--- 128

BnaA06_random.TIP2_3b -------PTHGVGAGLGVVEAIVMEIIVTFALVYTVYATAADPKKGS--- 168

BnaC07.TIP2_3a -------PTHGVGAGLGAVEAIVMEIIVTFALVYTVYATAADPKKGS--- 168

BnaC04.TIP4_1a -------PVHTLASGISYTQGIVWEIILTFSLLFTVYATMVDPKKGS--- 164

BnaCnn_random.TIP5_1a -------PIYKIAGEMTGFGASVLEGVLAFVLVYTVF-TANDPRRGL--- 171

BnaA06.TIP5_1a -------PIYKIAGEMTGFGASVLEGVLAFVLVYTVF-TANDPRRGL--- 171

BnaCnn_random.PIP2_1b RY---GGGANSLADGYSTGTGLAAEIIGTFVLVYTVFSATDPKRSAR--- 196

BnaA09.PIP2_1a RY---GGGANSLADGYSTGTGLAAEIIGTFVLVYTVFSATDPKRSAR--- 196

BnaC06.PIP2_1a RY---GGGANSLADGYSTGTGLAAEIIGTFVLVYTVFSATDPKRSAR--- 196

BnaC04.PIP2_2/2_3b RY---GGGANSLADGYSTGTGLAAEIIGTFVLVYTVFSATDPKRNAR--- 194

BnaA05.PIP2_2/2_3c RY---GGGANSLADGYSTGTGLAAEIIGTFVLVYTVFSATDPKRNAR--- 194

BnaC04.PIP2_2/2_3a RY---GGGANSLADGYSTGTGLAAEIIGTFVLVYTVFSATDPKRNAR--- 194

BnaA03.PIP2_2/2_3a RY---GGGANSLADGYSTGTGLAAEIIGTFVLVYTVFSATDPKRNAR--- 194

BnaA03.PIP2_2/2_3b RY---GGGANSLADGYSTGTGLAAEIIGTFVLVYTVFSATDPKRNAR--- 194

BnaC03.PIP2_4a RY---GGGANQLADGYNKGTGLGAEIIGTFVLVYTVFSATDPKRSAR--- 171

BnaA03.PIP2_4b RY---GGGANQLADGYNKGTGLGAEIIGTFVLVYTVFSATDPKRSAR--- 171

BnaA10.PIP2_4c RY---GGGANELADGYNKGTGLGAEIIGTFVLVYTVFSATDPKRSAR--- 171

BnaC09_random.PIP2_4b RY---GGGANELADGYNKGTGLGAEIIGTFVLVYTVFSATDPKRSAR--- 171

BnaA02.PIP2_4a RY---GGGANQLAEGYNKGTGLGAEIIGTFVLVYTVFSATDPKRSAR--- 169

BnaC08.PIP2_5b RY---GGGANGLSNGYSVGTGVAAEIIGTFVLVYTVFSATDPKRSAR--- 195

BnaA09.PIP2_5b RY---GGGANGLSNGYSVGTGVAAEIIGTFVLVYTVFSATDPKRSAR--- 195

BnaC06.PIP2_5a RY---GGGANGLTHGYSIGTGVAAEIIGTFVLVYTVFSATDPKRSAR--- 195

BnaA07.PIP2_5a RY---GGGANGLTHGYSIGTGVAAEIIGTFVLVYTVFSATDPKRSAR--- 195

BnaC03.PIP2_6a RY---GGGANVLADGYNVGVGVGAEIIGTFVLVYTVFSATDPKRNAR--- 194

BnaA03.PIP2_6a RY---GGGANVLADGYNVGVGVGAEIIGTFVLVYTVFSATDPKRNAR--- 194

BnaAnn_random.PIP2_7b TL---GGGANTVADGYSTGTALGAEIIGTFVLVYTVFSATDPKRSAR--- 190

BnaC01.PIP2_7a TL---GGGANTVADGYSTGTALGAEIIGTFVLVYTVFSATDPKRSAR--- 190

BnaC03.PIP2_7b TL---GGGANTVAPGYSKGTALGAEIIGTFVLVYTVFSATDPKRSAR--- 190

BnaA08.PIP2_7a TL---GGGANTVAPGYSKGTALGAEIIGTFVLVYTVFSATDPKRSAR--- 190

BnaC07.PIP2_7c TL---GGGANMVADGYSNGTALGAEIIGTFVLVYTVFSATDPKRSAR--- 163

BnaC07.PIP1_5a AN---GGGANLVAHGYTKGSGLGAEIIGTFVLVYTVFSATDAKRSAR--- 204

BnaA03.PIP1_5a SN---GGGANLVAHGYTKGSGLGAEIVGTFVLVYTVFSATDAKRSAR--- 204

BnaCnn_random.PIP1_3b TL---GGGANTVAPGYTKGSGLGAEIIGTFVLVYTVFSATDAKRSAR--- 203

BnaC05.PIP1_3a TL---GGGANTVAPGYTKGSGLGAEIIGTFVLVYTVFSATDAKRSAR--- 203

BnaA10.PIP1_3a TL---GGGANTVAPGYSKGSGLGAEIIGTFVLVYTVFSATDAKRSAR--- 203

BnaCnn_random.PIP1_4b TL---GGGANTVAPGYSKGSGLGAEIIGTFVLVYTVFSATDAKRSAR--- 203

BnaA09_random.PIP1_4b TL---GGGANTVAPGYSKGSGLGAEIIGTFVLVYTVFSATDAKRSAR--- 203

BnaC03.PIP1_4a TL---GGGANTVAPGYSKGSGLGAEIIGTFVLVYTVFSATDAKRSAR--- 203

BnaA03.PIP1_4a TL---GGGANTVAPGYSKGSGLGAEIIGTFVLVYTVFSATDAKRSAR--- 203

BnaA09.PIP1_1b AL---GGGANTVAPGYTKGSGLGAEIIGTFVLVYTVFSATDAKRNAR--- 203

BnaC08.PIP1_1a AL---GGGANTVAPGYTKGSGLGAEIIGTFVLVYTVFSATDAKRNAR--- 203

BnaA04.PIP1_1a AL---GGGANTVAPGYTKGSGLGAEIIGTFVLVYTVFSATDAKRNAR--- 142

BnaAnn_random.PIP1_1c AL---GGGSNTVAPGYTKGSGLGAEIIGTFVLVYTVFSATDAKRNAR--- 123

BnaC04.PIP1_2b AL---GGGANTVAHGYTKGSGLGAEIIGTFVLVYTVFSATDAKRNAR--- 203

BnaA05.PIP1_2c AL---GGGANTVAPGYTKGSGLGAEIIGTFVLVYTVFSATDAKRNAR--- 203

BnaC03.PIP1_2a AL---GGGANTVAPGYTKGSGLGAEIIGTFVLVYTVFSATDAKRNAR--- 203

BnaA03.PIP1_2a AL---GGGANTVAPGYTKGSGLGAEIIGTFVLVYTVFSATDAKRNAR--- 203

BnaC04.PIP1_2c AL---GGGANTVAPGYTKGSGLGAEIIGTFVLVYTVFSATDAKRNAR--- 203

BnaA04.PIP1_2b AL---GGGANTVAPGYTKGSGLGAEIIGTFVLVYTVFSATDAKRNAR--- 203

BnaC07.NIP1_2b VCSGKHDVFVGTLPSGSDLQSFVIEFIITFYLMFIISGVATDNRAIG-EL 209

BnaA03.NIP1_2b VCSGKHDVFVGTLPSGSDLQSFVIEFIITFYLMFIISGVATDNRAIG-EL 209

BnaC01.NIP1_2a VCSGKHDVFVGTLPAGSDLQSFVIEFIITFYLMFIISGVATDNRAIG-EL 208

BnaA01.NIP1_2a VCSGKHDVFVGTLPAGSDLQSFVIEFIITFYLMFIISGVATDNRAIG-EL 208

BnaC04.NIP2_1b VCSKKHDVFLGSSPSGTDLQAFVMEFIITGFLMIVVCAFTTSKRTTK-EL 198

BnaA05.NIP2_1b VCSKKHDVFLGSSPSGTDLQAFGMEFIITGFLMIVICAITTSKRTTK-EL 198

BnaC04.NIP2_1a VCSKKHDVFLGSTPSGTDLQAFGMEFIITGFLMIVVCAVTTSKRTSE-EL 198

BnaA05.NIP2_1a VCSKKHDVFLGSSPSGTDLQAFGMEFIITGFLMIVVCAVTTSKRTSE-EL 198

BnaA04.NIP4_1a ------AAYFGTIPSDSAAQALAAEIIISFLLMFVISGVATDNRAVG-EL 189

BnaC04.NIP4_1a ------AAYFGTTPSDSAAQALAAEIIISFLLMFVISGVATDNRAVG-EL 189

BnaC04.NIP4_1b ------EAFFGTTPADSAARALVSEIIISFLLMFVISGVATDSRAIG-EL 189

BnaC06_random.NIP4_2a ------EAFFGTTPADSVARALVAEIIISFLLMFVISGVATDSRAIG-EL 194

BnaA04_random.NIP4_1b ------EAYFGTTPTDSAARALVAEIIISFLLMFVISGVSTDSRAIG-EL 141

BnaC08.NIP3_1c VCSLKGDVYVGTYPSSSNTATFVMEFITTFNLMFVISAVATDKRANG-SF 196

BnaA08.NIP3_1c VCSLKGDIYVGTYPSSSNTATFVMEFITTFNLMFVISAVATDKRANG-SF 169

BnaC05.NIP3_1b VCSLKGDVYVGTHPS------------------------ATDKRANR-SF 186

BnaC05.NIP3_1a VCSLKGDVYVGTHPS------------------------ATDKRANR-SF 186

BnaA05.NIP3_1b VCSLKGDVYVGTRPS------------------------ATDKRANR-SF 186

BnaA05.NIP3_1a GCSLKGAIYVGTHPSSSNTASFVVEFIATFNLLFVISAVATDKRANR-SF 193

BnaC03.NIP5_1b GG--------VTVPSVSVGQAFALEFIISFILLFVVTAVATDTRAVG-EL 220

BnaA03.NIP5_1b GG--------VTVPSVSVGQAFALEFIISFILLFVVTAVATDTRAVG-EL 220

BnaC02.NIP5_1a GG--------VTVPSVSVGQAFALEFIITFILLFVVTAVATDTRAVG-EL 220

BnaA02.NIP5_1a GG--------VTVPSVSVGQAFALEFIITFILLFVVTAVATDTRAVG-EL 220

BnaA07.NIP5_1c GG--------VTVPSVSVGQAFALEFIISFILLFVITAVATDTRAVG-EL 220

BnaC06_random.NIP5_1c GG--------VTVPSVSVGQAFALEFIISFILLFVITAVATDTRAVG-EL 220

BnaA02_random.NIP6_1c GG--------VTVPTVTLSQAFALEFIITFNLMFVVTAVATDTRAVG-EL 225

BnaA02.NIP6_1a GG--------VTVPTVALSQAFALEFIISFNLMFVVTAVATDTRAVG-EL 225

BnaC06.NIP6_1a GG--------VTVPTVGLSQAFALEFIISFNLMFVVTAVATDTRAVG-EL 225

BnaA07.NIP6_1b GG--------VTVPTVGLSQAFALEFIISFNLMFVVTAVATDTRAVG-EL 225

BnaC05.NIP7_1a --------LMITKPALSCVSAFFVELVATSIVVFLASALHCGPHQNLSNL 191

BnaA05.NIP7_1a --------LMITKPALSCVSAFFVELVATSIVVFLASALHCGPHQNSSNL 191

BnaA05_random.SIP1_1b HM-ISG-PSLQVDVHTGAIAETILSFGITFAALLIIIRGPRRLLAKT--- 161

BnaC05.SIP1_1b HM-ISG-PSLQVDVHTGAIAETILSFGITFAALLIIIRGPRRLLAKT--- 161

BnaC01.SIP1_1a HM-ISG-PSLLVDVHTGAIAETILSFGITFAVLLIILKGPRRLLAKT--- 161

BnaA01.SIP1_1a HM-ISG-PSLLVDVHTGAIAETILSFGITFAVLLIILKGPRRLLAKT--- 161

BnaC09_random.SIP1_2a TM-IGGRPSFRVDAHSGAISEVILSFCVTFLVLLIILRGPRKLLAKT--- 165

BnaA10.SIP1_2a TM-IGGRPSFRVDAHSGAISEVILSFSVTFLVLLIILRGPRKLLAKT--- 165

BnaCnn_random.SIP2_1c -----G-PKLNVAIHHGALTEGILTFFIVMLSLGLTRKIPGSFFMKT--- 157

BnaA07.SIP2_1a -----G-PKLNVAIHHGALTEGILTFFIVMLSLGLTRKIPGSFFMKT--- 157

BnaC04.SIP2_1b -----G-PKLNVAIHHGALTEGILTFFIVMLSLGLARKIPGSFFMKT--- 157

BnaC03.SIP2_1a -----G-PKLNVAIHHGALTEGILTFFIVMLSLGLARKIPGSFFMKT--- 157

BnaA09.SIP2_1b -----G-PKLNVSIHQGALTEGVLTFFTVLISMELSRKIPGSFFMKT--- 154

BnaAnn_random.TIP1_1b LGTIA----------------------------PIAIGFIVGANILAGGA 162

BnaAnn_random.TIP1_1a LGTIA----------------------------PIAIGFIVGANILAGGA 162

BnaCnn_random.TIP1_1a LGTIA----------------------------PIAIGFIVGADILAGGA 162

BnaC07.TIP1_2b LGTIA----------------------------PIAIGFIVGANILAGGA 193

BnaA06.TIP1_2b LGTIA----------------------------PIAIGFIVGANILAGGA 193

BnaC02.TIP1_2a LGTIA----------------------------PIAIGFIVGANILAGGA 193

BnaA02.TIP1_2a LGTIA----------------------------PIAIGFIVGANILAGGA 193

BnaCnn_random.TIP1_3a IGIIA----------------------------PLAIGLIVGANILVGGA 192

BnaA09.TIP1_3a IGIIA----------------------------PLAIGLIVGANILVGGA 192

BnaCnn_random.TIP3_1c LGVIG----------------------------PLAIGLIVGANILVGGP 200

BnaA02.TIP3_1a LGVIG----------------------------PLAIGLIVGANILVGGQ 200

BnaC06.TIP3_1a LGVIG----------------------------PLAVGLIVGANILVGGP 200

BnaA07.TIP3_1b LGVIG----------------------------PLAVGLIVGANILVGGP 200

BnaC06.TIP3_1b LGIIG----------------------------PLAVGLIVGANILMGGP 200

BnaA07.TIP3_1c LGIIG----------------------------PLAVGLIVGANILMGGP 200

BnaC08.TIP3_2b IGIIA----------------------------PLAIGLIVGANMLVGGP 200

BnaA09.TIP3_2b IGIIA----------------------------PLAIGLIVGANMLVGGP 200

BnaC05.TIP3_2a IGIIA----------------------------PLAIGLIVGANMLVGGP 200

BnaA06.TIP3_2a IGIIA----------------------------PLAIGLIVGANMLVGGP 200

BnaC01_random.TIP2_1d LGTIA----------------------------PLAIGLIVGANILAAGP 190

BnaA01.TIP2_1a LGTIA----------------------------PLAIGLIVGANILAAGP 190

BnaC05.TIP2_1b LGTIA----------------------------PLAIGLIVGANILAAGP 190

BnaA05.TIP2_1c LGTIA----------------------------PLAIGLIVGANILAAGP 190

BnaC03.TIP2_1a LGTIA----------------------------PLAIGLIVGANILAAGP 190

BnaA03.TIP2_1b LGTIA----------------------------PLAIGLIVGANILAAGP 190

BnaC06.TIP2_1c LGIIA----------------------------PLAIGLIVGANILAAGP 189

BnaC01_random.TIP2_2a LGTIA----------------------------PIAIGFIVGANILAAGP 190

BnaA01_random.TIP2_2a LGTIA----------------------------PIAIGFIVGANILAAGP 190

BnaC02_random.TIP2_3b LGTIA----------------------------PIAIGFIVGANILAAGP 190

BnaA02.TIP2_3a LGTIA----------------------------PIAIDFIVGANILAAGP 150

BnaA06_random.TIP2_3b LGTIA----------------------------PIAIGFIVGANILAAGP 190

BnaC07.TIP2_3a LGTIA----------------------------PIAIGFIVGANILAAGP 190

BnaC04.TIP4_1a LDGLG----------------------------PLLTGFVVGANILAGGA 186

BnaCnn_random.TIP5_1a PLAVG----------------------------PIFIGFVAGANVLAAGP 193

BnaA06.TIP5_1a PLAVG----------------------------PIFIGFVAGANVLAAGP 193

BnaCnn_random.PIP2_1b DSHVP-------------------------VLAPLPIGFAVFMVHLATIP 221

BnaA09.PIP2_1a DSHVP-------------------------VLAPLPIGFAVFMVHLATIP 221

BnaC06.PIP2_1a DSHVP-------------------------VLAPLPIGFAVFMVHLATIP 221

BnaC04.PIP2_2/2_3b DSHVP-------------------------VLAPLPIGFAVFMVHLATIP 219

BnaA05.PIP2_2/2_3c DSHVPVSVGATPNWICRVHGTLGHYSNHRNVLAPLPIGFAVFMVHLATIP 244

BnaC04.PIP2_2/2_3a DSHVP-------------------------VLAPLPIGFAVFMVHLATIP 219

BnaA03.PIP2_2/2_3a DSHVP-------------------------VLAPLPIGFAVFMVHLATIP 219

BnaA03.PIP2_2/2_3b DSHVP-------------------------VLAPLPIGFAVFMVHLATIP 219

BnaC03.PIP2_4a DSHIP-------------------------VLAPLPIGFAVFMVHLATIP 196

BnaA03.PIP2_4b DSHIP-------------------------VLAPLPIGFAVFMVHLATIP 196

BnaA10.PIP2_4c DSHIP-------------------------VLAPLPIGFAVFMVHLATIP 196

BnaC09_random.PIP2_4b DSHIP-------------------------VLAPLPIGFAVFMVHVATIP 196

BnaA02.PIP2_4a DSHIP-------------------------VLAPLPIGFAVFMVHLATIP 194

BnaC08.PIP2_5b DSHVP-------------------------VLAPLPIGFAVFIVHLATIP 220

BnaA09.PIP2_5b DSHVP-------------------------VLAPLPIGFAVFIVHLATIP 220

BnaC06.PIP2_5a DSHVP-------------------------VLAPLPIGFAVFIVHLATIP 220

BnaA07.PIP2_5a DSHVP-------------------------VLAPLPIGFAVFIVHLATIP 220

BnaC03.PIP2_6a DSHIP-------------------------VLAPLPIGFSVFMVHLATIP 219

BnaA03.PIP2_6a DSHIP-------------------------VLAPLPIGFSVFMVHLATIP 219

BnaAnn_random.PIP2_7b DSHIP-------------------------VLAPLPIGFAVFMVHLATIP 215

BnaC01.PIP2_7a DSHIP-------------------------VLAPLPIGFAVFMVHLATIP 215

BnaC03.PIP2_7b DSHIP-------------------------VLAPLPIGFAVFMVHLATIP 215

BnaA08.PIP2_7a DSHIP-------------------------VLAPLPIGFAVFMVHLATIP 215

BnaC07.PIP2_7c DSHIP-------------------------VLAPLPIGFAVFMVHLATIP 188

BnaC07.PIP1_5a DSHVP-------------------------ILAPLPIGFAVFLVHLATIP 229

BnaA03.PIP1_5a DSHVP-------------------------ILAPLPIGFAVFLVHLATIP 229

BnaCnn_random.PIP1_3b DSHVP-------------------------ILAPLPIGFAVFLVHLATIP 228

BnaC05.PIP1_3a DSHVP-------------------------ILAPLPIGFAVFLVHLATIP 228

BnaA10.PIP1_3a DSHVP-------------------------ILAPLPIGFAVFLVHLATIP 228

BnaCnn_random.PIP1_4b DSHVP-------------------------ILAPLPIGFAVFLVHLATIP 228

BnaA09_random.PIP1_4b DSHVP-------------------------ILAPLPIGFAVFLVHLATIP 228

BnaC03.PIP1_4a DSHVP-------------------------ILAPLPIGFAVFLVHLATIP 228

BnaA03.PIP1_4a DSHVP-------------------------ILAPLPIGFAVFLVHLATIP 228

BnaA09.PIP1_1b DSHVP-------------------------ILAPLPIGFAVFLVHLATIP 228

BnaC08.PIP1_1a DSHVP-------------------------ILAPLPIGFAVFLVHLATIP 228

BnaA04.PIP1_1a DSHVP-------------------------ILAPLPIGFAVFLVHLATIP 167

BnaAnn_random.PIP1_1c DSHVP-------------------------ILAPLPIGFAVFLVHLATIP 148

BnaC04.PIP1_2b DSHVP-------------------------ILAPLPIGFAVFLVHLATIP 228

BnaA05.PIP1_2c DSHVP-------------------------ILAPLPIGFAVFLVHLATIP 228

BnaC03.PIP1_2a DSHVP-------------------------ILAPLPIGFAVFLVHLATIP 228

BnaA03.PIP1_2a DSHVP-------------------------ILAPLPIGFAVFLVHLATIP 228

BnaC04.PIP1_2c DSHVP-------------------------ILAPLPIGFAVFLVHLATIP 228

BnaA04.PIP1_2b DSHVP-------------------------ILAPLPIGFAVFLVHLATIP 228

BnaC07.NIP1_2b AG--------------------------------LAVGSTVLLNVIIAGP 227

BnaA03.NIP1_2b AG--------------------------------LAVGSTVLLNVIIAGP 227

BnaC01.NIP1_2a AG--------------------------------LAVGSTVLLNVIIAGP 226

BnaA01.NIP1_2a AG--------------------------------LAVGSTVLLNVIIAGP 226

BnaC04.NIP2_1b EG--------------------------------LIIGATVTLNVIFAGE 216

BnaA05.NIP2_1b EG--------------------------------LIIGATVTLNVIFAGE 216

BnaC04.NIP2_1a EG--------------------------------LIIGATVTLNVIFAGE 216

BnaA05.NIP2_1a EG--------------------------------LIIGATVTLNVIFAGE 216

BnaA04.NIP4_1a AG--------------------------------IAVGMTIMLNVFVAGP 207

BnaC04.NIP4_1a AG--------------------------------IAVGMTIILNVFVAGP 207

BnaC04.NIP4_1b AG--------------------------------IAVGMTIILNVFVAGP 207

BnaC06_random.NIP4_2a AG--------------------------------IAVGMTIILNVFVAGP 212

BnaA04_random.NIP4_1b AG--------------------------------IAVGMTIMLNVFVAGP 159

BnaC08.NIP3_1c AG--------------------------------IAIGATVVLDILFCGP 214

BnaA08.NIP3_1c AG--------------------------------IAIGATVVLDILFCGP 187

BnaC05.NIP3_1b AG--------------------------------VAIGATVVLNILFSGP 204

BnaC05.NIP3_1a AG--------------------------------VAIGATVVLNILFSGP 204

BnaA05.NIP3_1b AG--------------------------------VAIGATVVLNILFSGP 204

BnaA05.NIP3_1a AG--------------------------------LAIGATVVLDILFGGP 211

BnaC03.NIP5_1b AG--------------------------------IAVGATVMLNILVAGP 238

BnaA03.NIP5_1b AG--------------------------------IAVGATVMLNILVAGP 238

BnaC02.NIP5_1a AG--------------------------------IAVGATVMLNILVAGP 238

BnaA02.NIP5_1a AG--------------------------------IAVGATVMLNILVAGP 238

BnaA07.NIP5_1c AG--------------------------------IAVGATVMLNNLVAGP 238

BnaC06_random.NIP5_1c AG--------------------------------IAVGATVMLNNLVAGP 238

BnaA02_random.NIP6_1c AG--------------------------------IAVGATVMLNILIAGP 243

BnaA02.NIP6_1a AG--------------------------------IAVGATVMLNILIAGP 243

BnaC06.NIP6_1a AG--------------------------------IAVGATVMLNILIAGP 243

BnaA07.NIP6_1b AG--------------------------------IAVGATVMLNILIAGP 243

BnaC05.NIP7_1a TG--------------------------------LVIGAVISLGVLITGP 209

BnaA05.NIP7_1a TG--------------------------------LVIGAVISLGVLITGP 209

BnaA05_random.SIP1_1b ----------------------------------FLLALATICFVVAGSK 177

BnaC05.SIP1_1b ----------------------------------FLLALATICFVVAGSK 177

BnaC01.SIP1_1a ----------------------------------LLLSLATICFVVAGSK 177

BnaA01.SIP1_1a ----------------------------------LLLSLATICFVVAGSK 177

BnaC09_random.SIP1_2a ----------------------------------FLLAIATVSVFIAGST 181

BnaA10.SIP1_2a ----------------------------------FLLAIATVSVFIAGST 181

BnaCnn_random.SIP2_1c ----------------------------------WIGSIAKLTLHVLGAD 173

BnaA07.SIP2_1a ----------------------------------WIGSIAKLTLHVLGAD 173

BnaC04.SIP2_1b ----------------------------------WIGSIAKLTLHVLGAD 173

BnaC03.SIP2_1a ----------------------------------WIGSIAKLTLHVLGAD 173

BnaA09.SIP2_1b ----------------------------------WISSIAKLSLHVLGAD 170

.

**（P2）（P3） （P4, P5）**

BnaAnn_random.TIP1_1b FSGASMNPAVAFGPAVVSWS---WNNHW-VYWAGPLVGGGLAGLIYEVFF 208

BnaAnn_random.TIP1_1a FSGASMNPAVAFGPAVVSWS---WNNHW-VYWAGPLVGGGLAGLIYEVFF 208

BnaCnn_random.TIP1_1a FSGASMNPAVAFGPAVVSWS---WNNHW-VYWAGPLIGGGVAGLIYEVFF 208

BnaC07.TIP1_2b FSGASMNPAVAFGPAVVSWT---WTNHW-IYWAGPLVGGGLAGLIYEFVF 239

BnaA06.TIP1_2b FSGASMNPAVAFGPAVVSWT---WTNHW-IYWAGPLVGGGLAGLIYEFVF 239

BnaC02.TIP1_2a FSGASMNPAVAFGPAVVSWS---WSNHW-IYWVGPLVGGGLAGIIYDFVY 239

BnaA02.TIP1_2a FSGASMNPAVAFGPAVVSWS---WSNHW-IYWVGPLVGGGLAGIIYDFVY 239

BnaCnn_random.TIP1_3a FDGASMNPAVSFGPAVVSWT---WTNHW-VYWVGPFIGAAIAAIVYDTIF 238

BnaA09.TIP1_3a FDGASMNPAVSFGPAVVSWT---WTNHW-VYWVGPFIGAAIAAIVYDTIF 238

BnaCnn_random.TIP3_1c FSGASMNPARAFGPALVGWR---WDDHW-IYWVGPFIGGALAALIYEYMV 246

BnaA02.TIP3_1a FSGASMNPARAFGPALVGWR---WDDHW-IYWVGPFIGGALAALIYEYMV 246

BnaC06.TIP3_1a FSGASMNPARAFGPALVGWR---WDDHW-IYWVGPFIGGALAALIYEYMV 246

BnaA07.TIP3_1b FSGASMNPARAFGPALVGWR---WDDHW-IYWVGPFIGGALAALIYEYMV 246

BnaC06.TIP3_1b FSGASMNPARAFGPALVGWR---WDDHW-IYWVGPFIGGALAAFIYEFMV 246

BnaA07.TIP3_1c FSGASMNPARAFGPALVGWR---WDDHW-IYWVGPFIGGALAAFIYEFMV 246

BnaC08.TIP3_2b FEGASMNPARAFGPSLVGWR---WHNHW-IYWVGPFIGGALAALIYEYMI 246

BnaA09.TIP3_2b FEGASMNPARAFGPSLVGWR---WHNHW-IYWVGPFIGGALAALIYEYMI 246

BnaC05.TIP3_2a FDGASMNPARAFGPSLVGWR---WENHW-IYWVGPFIGGALAALIYEYMI 246

BnaA06.TIP3_2a FDGASMNPARAFGPSLVGWR---WDNHW-IYWVGPFIGGALAALIYEYMI 246

BnaC01_random.TIP2_1d FSGGSMNPARSFGPAVAAGD---FSGHW-VYWVGPLIGGGLAGLVYGNVF 236

BnaA01.TIP2_1a FSGGSMNPARSFGPAVAAGD---FSGHW-VYWVGPLIGGGLAGLVYGNVF 236

BnaC05.TIP2_1b FSGGSMNPARSFGPAVAAGD---FSGHW-VYWVGPLIGGGLAGITYGNVF 236

BnaA05.TIP2_1c FSGGSMNPARSFGPAVAAGD---FSGHW-VYWVGPLIGGGLAGITYGNVF 236

BnaC03.TIP2_1a FSGGSMNPARSFGPAVAAGD---FSGHW-VYWVGPLIGGGLAGLIYGNVF 236

BnaA03.TIP2_1b FSGGSMNPARSFGPAVAAGD---FSGHW-VYWVGPLIGGGLAGLIYGNVF 236

BnaC06.TIP2_1c FSGGSMNPARSFGPALVAGD---FSGHW-VYWVGPLVGGGLAGVIYSNAF 235

BnaC01_random.TIP2_2a FSGGSMNPARSFGPAVVSGD---FSQIW-IYWVGPLVGGALAGLIYGDVF 236

BnaA01_random.TIP2_2a FSGGSMNPARSFGPAVVSGD---FSQIW-IYWVGPLVGGALAGLIYGDVF 236

BnaC02_random.TIP2_3b FSGGSMNPARSFGPSVVSGD---LSQIW-IYWVGPLVGGGLAGLIYGDVF 236

BnaA02.TIP2_3a FSGGSMNPARSFGPAVVSGD---LSQIW-IYWVGPLVGGGLAGLIYGDVF 196

BnaA06_random.TIP2_3b FSGGSMNPARSFGPAIVSGD---LSQIW-IYWVGPLVGGALAGLIYGDVF 236

BnaC07.TIP2_3a FSGGSMNPARSFGPAIVSGD---LSQIW-IYWVGPLVGGALAGLIYGDVF 236

BnaC04.TIP4_1a FSGASMNPARSFGPALVSGN---WTDHW-VYWVGPLIGGGLAGFIYENVL 232

BnaCnn_random.TIP5_1a FSGGAMNPACAFGSAMIYGS---FKNQA-VYWVGPLLGGATAALVYDNMV 239

BnaA06.TIP5_1a FSGGAMNPACAFGSAMIYGS---FKNQA-VYWVGPLLGGATAAFVYDNMV 239

BnaCnn_random.PIP2_1b ITGTGINPARSFGAAVIFNESKPWDDHW-IFWVGPFVGAAIAAFYHQFVL 270

BnaA09.PIP2_1a ITGTGINPARSFGAAVIFNESKPWDDHW-IFWVGPFVGAAIAAFYHQFVL 270

BnaC06.PIP2_1a ITGTGINPARSLGAAVIFNESKPWDDHW-IFWLGPFIGAAIAAVYHQFVL 270

BnaC04.PIP2_2/2_3b ITGTGINPARSFGAAVIYNESKPWDDHW-IFWVGPFIGAAIAAFYHQFVL 268

BnaA05.PIP2_2/2_3c ITGTGINPARSFGAAVIYNESKPWDDHW-IFWVGPFIGAAIAAFYHQFVL 293

BnaC04.PIP2_2/2_3a ITGTGINPARSFGAAVIFNESKPWNDHW-IFWVGPFIGAAIAAFYHQFVL 268

BnaA03.PIP2_2/2_3a ITGTGINPARSFGAAVIYNESKPWDDHW-IFWVGPFIGAAIAAFYHQFVL 268

BnaA03.PIP2_2/2_3b ITGTGINPARSFGAAVIYNESKPWDDHW-IFWVGPFIGAAIAAFYHQFVL 268

BnaC03.PIP2_4a ITGTGINPARSFGAAVIYNHEKAWDDQW-IFWVGPMIGAAAAALYHQFVL 245

BnaA03.PIP2_4b ITGTGINPARSFGAAVIYNQEKAWDDQW-IFWVGPMIGAAAAALYHQFVL 245

BnaA10.PIP2_4c ITGTGINPARSFGAAVIYNQEKAWDDQW-IFWVGPMIGAAAAALYHQFVL 245

BnaC09_random.PIP2_4b ITGTGINPARSFGAAVIYNQEKAWDDQ--IFWVGQMIGAAAAALYHQFVL 244

BnaA02.PIP2_4a ITGTGINPARSFGAAVIYNQEKAWDDQQWIFWVGPMIGAAAAALYHQFVL 244

BnaC08.PIP2_5b ITGTGINPARSLGAAIIYNKDQAWDHHW-IFWAGPFAGATIAAFYHQFVL 269

BnaA09.PIP2_5b ITGTGINPARSLGAAIIYNKDQAWDHHW-IFWAGPFAGAAIAAFYHQFVL 269

BnaC06.PIP2_5a ITGTGINPARSLGAAIIYNKDQAWDHHW-IFWVGPFAGAALAAFYHQFVL 269

BnaA07.PIP2_5a ITGTGINPARSLGAAIIYNKDQAWDHHW-IFWVGPFAGAALAAFYHQFVL 269

BnaC03.PIP2_6a ITGTGINPARSFGAAVIYNNQKAWDDQW-IFWVGPFVGASIAALYHQFVL 268

BnaA03.PIP2_6a ITGTGINPARSFGAAVIYNNQKAWDDQW-IFWVGPFVGAAIAALYHQFVL 268

BnaAnn_random.PIP2_7b ITGTGINPARSFGAAVIYNNEKAWDDHW-IFWVGPFVGALAAAAYHQYIL 264

BnaC01.PIP2_7a ITGTGINPARSFGAAVIYNNEKAWDDHW-IFWVGPFVGALAAAAYHQYIL 264

BnaC03.PIP2_7b ITGTGINPARSFGAAVIYNNEKAWDDHW-IFWVGPFVGALAAAAYHQYIL 264

BnaA08.PIP2_7a ITGTGINPARSFGAAVIYNHEKAWDDHW-IFWVGPFVGALAAAAYHQYIL 264

BnaC07.PIP2_7c ITGTGINPARSFGAAVIYNNEKAWDDHW-IFWVGPFVGALAAAAYHQYIL 237

BnaC07.PIP1_5a ITGTGINPARSLGAAIIYNKDHAWGDHW-IFWVGPFIGAALAALYHQIVI 278

BnaA03.PIP1_5a ITGTGINPARSLGAAIIYNKDHAWDDHW-IFWVGPFIGAALAALYHQIVI 278

BnaCnn_random.PIP1_3b ITGTGINPARSLGAAIIYNKDHAWDDHW-IFWVGPFIGAALAALYHQLVI 277

BnaC05.PIP1_3a ITGTGINPARSLGAAIIYNKDHSWDDHW-IFWVGPFIGAALAALYHQLVI 277

BnaA10.PIP1_3a ITGTGINPARSLGAAIIYNKDHSWDDHW-IFWVGPFIGAALAALYHQLVI 277

BnaCnn_random.PIP1_4b ITGTGINPARSLGAAIIYNKDHSWDNHW-IFWVGPFIGAALAALYHTIVI 277

BnaA09_random.PIP1_4b ITGTGINPARSLGAAIIYNKDHSWDDHW-IFWVGPFIGAALAALYHTIVI 277

BnaC03.PIP1_4a ITGTGINPARSLGAAIIYNKDHSWDDHW-IFWVGPFIGAALAALYHTIVI 277

BnaA03.PIP1_4a ITGTGINPARSLGAAIIYNKDHSWDDHW-IFWVGPFIGAALAALYHTIVI 277

BnaA09.PIP1_1b ITGTGINPARSLGAAIIYNKDHSWDDHW-VFWVGPFIGAALAALYHVIVI 277

BnaC08.PIP1_1a ITGTGINPARSLGAAIIYNKDHSWDDHW-VFWVGPFIGAALAALYHVIVI 277

BnaA04.PIP1_1a ITGTGINPARSLGAAIIYNKDHSWDDHW-VFWVGPFIGAALAALYHVIVI 216

BnaAnn_random.PIP1_1c ITGTGINPARSLGAAIIYNKDHSWDDHW-VFWVGPFIGAAFAALYHVIVI 197

BnaC04.PIP1_2b ITGTGINPARSLGAAIIFNKDNAWDDHW-VFWVGPFIGAALAALYHVIVI 277

BnaA05.PIP1_2c ITGTGINPARSLGAAIIFNKDNAWDDHW-VFWVGPFIGAALAALYHVIVI 277

BnaC03.PIP1_2a ITGTGINPARSLGAAIIFNKDNAWDDHW-VFWVGPFIGAALAALYHVIVI 277

BnaA03.PIP1_2a ITGTGINPARSLGAAIIFNKDNAWDDHW-VFWVGPFIGAALAALYHVIVI 277

BnaC04.PIP1_2c ITGTGINPARSLGAAIIFNKDNAWDDHW-VFWVGPFIGAALAALYHVIVI 277

BnaA04.PIP1_2b ITGTGINPARSLGAAIIFNKDNAWDDHW-VFWVGPFIGAALAALYHVIVI 277

BnaC07.NIP1_2b VSGASMNPGRSLGPAMVYNC---YKGIW-IYIASPILGAVAGAWVYNTVR 273

BnaA03.NIP1_2b VSGASMNPGRSLGPAMVYNC---YKGIW-IYIASPILGAVAGAWVYNTVR 273

BnaC01.NIP1_2a VSGASMNPGRSLGPAMVYNC---YRGIW-IYIASPILGAVAGAWVYNTVR 272

BnaA01.NIP1_2a VSGASMNPGRSLGPAMVYNC---YKGIW-IYIASPILGAVAGAWVYNTVR 272

BnaC04.NIP2_1b VSGASMNPARSLGPALVWGC---YKGIW-IYLVAPTLGAVSAALIHKLLP 262

BnaA05.NIP2_1b VSGASMNPARSIGPALVWGC---YKGIW-IYLLAPTLGAVSAALIHKLLP 262

BnaC04.NIP2_1a VSGASMNPARSLGPALVWGC---YKGIW-IYLLAPTLGAVSAALIHKLLP 262

BnaA05.NIP2_1a VSGASMNPARSLGPALVWGC---YKGIW-IYLLAPTLGAVLAALIHKLLP 262

BnaA04.NIP4_1a VSGASMNPARSLGPAIVMGV---YDGLW-IYIVGPLVGIMAGGFVYNLIR 253

BnaC04.NIP4_1a VSGASMNPARSLGPAIVMGV---YDGLW-IYIVGPLVGIMAGGFVYNLIR 253

BnaC04.NIP4_1b ISGASMNPARSLGPAIVMGV---YKGIW-VYIVGPIIGIVAGGFVYNFIR 253

BnaC06_random.NIP4_2a ISGASMNPARSLGPAIVMGV---YKGIW-IYIVGPIVGIMAGGFVYNFIR 258

BnaA04_random.NIP4_1b ISGASMNPARSLGPAIVMGV---YKDIW-VYIVGPIAGVMAGGFVYNFIR 205

BnaC08.NIP3_1c ISGASMNPARSLGPALIWGC---YKDLW-LYIVSPVIGALTGAWTYDMLR 260

BnaA08.NIP3_1c ISGASMNPARSLGPALIWGC---YKDLW-LYIVSPVIGALTGAWTYDMLR 233

BnaC05.NIP3_1b ISGASMNPARSLAPAYIWGC---YKDLW-LYIVSPVIGALTGAWIYNILR 250

BnaC05.NIP3_1a ISGASMNPARSLAPAYIWGC---YKDLW-LYIVSPVIGALTGAWIYNILR 250

BnaA05.NIP3_1b ISGASMNPARSLAPAYIWGC---YKNLW-LYIVSPVIGALIGAWTYNMLR 250

BnaA05.NIP3_1a ISGASMNPARSLAPAYIWGC---YKDLW-IYIVAPVVGALTGAWTYNMLR 257

BnaC03.NIP5_1b STGASMNPVRTLGPALASGN---YRSLW-VYLVAPTLGAISGAAVYTGVK 284

BnaA03.NIP5_1b STGASMNPVRTLGPALASGN---YRLLW-VYLVAPTLGAISGAAVYTGVK 284

BnaC02.NIP5_1a SSGGSMNPVRTLGPALASGN---YRSLW-VYLVAPTLGAISGAAVYTGVK 284

BnaA02.NIP5_1a SSGGSMNPVRTLGPALASGN---YRSLW-VYLVAPTLGAISGAAVYTGVK 284

BnaA07.NIP5_1c SSGASMNPVRTLGPALASGN---YRSLW-VYMVAPTLGAISGAAVYTGVK 284

BnaC06_random.NIP5_1c SSGASMNPVRTLGPALASGN---YRSLW-VYLVAPTLGAISGAAVYTGVK 284

BnaA02_random.NIP6_1c ATSASMNPVRTLGPAIAANN---YRAIW-VYLTAPILGALIGAGTYTVVK 289

BnaA02.NIP6_1a ATSASMNPVRTLGPAIAANN---YRAIW-VYLTAPILGALIGAGTYTVVK 289

BnaC06.NIP6_1a ATSASMNPVRTLGPAIAANN---YRAIW-VYLTAPILGALIGAGTYTIVK 289

BnaA07.NIP6_1b ATSASMNPVRTLGPAIAANN---YRAIW-VYLTAPILGALIGAGTYTIVK 289

BnaC05.NIP7_1a ISGGSMNPARSLGPAVVAWD---FEYIW-VYMTAPVIGAIMGVLTYRTIS 255

BnaA05.NIP7_1a ISGGSMNPARSLGPAVVAWD---FEYIW-VYMTAPVIGAIMGVLTYRTIS 255

BnaA05_random.SIP1_1b YTGPAMNPAIAFGWAYMYSSHNTWDHFY-VYWISSFVGALSAALVFRTIF 226

BnaC05.SIP1_1b YTGPAMNPAIAFGWAYMYSSHNTWDHFY-VYWISSFVGALSAALVFRTIF 226

BnaC01.SIP1_1a YTGPAMNPAIAFGWAYMTSSHNTWDHFY-VYWISSFVGALSAALVFRTIF 226

BnaA01.SIP1_1a YTGPAMNPAIAFGWAYMTSSHNTWDHFY-VYWISSFVGALSAALVFRTIF 226

BnaC09_random.SIP1_2a FTRPFMNPAIAFGWAYIHKSHNTWNHFY-VYWFSSFTGAILSAILFRSLF 230

BnaA10.SIP1_2a FTRPFMNPAIAFGWAYIHKSHNTWNHFY-VYWFSSFTGAILSAILFRSLF 230

BnaCnn_random.SIP2_1c LTGGCMNPAAVMGWAYARGEHITQEHLL-VYWLGPVKATLLAVWFFNVVF 222

BnaA07.SIP2_1a LTGGCMNPAAVMGWAYARGEHITQEHLL-VYWLGPVKATLLAVWFFNVVF 222

BnaC04.SIP2_1b LTGGCMNPAAVMGWAYARGEHITQEHLL-VYWLGPIKATLLAVWFFNVVF 222

BnaC03.SIP2_1a LTGGCMNPAAVMGWAYARGEHITQEHLL-VYWLGPIKATLLAVWFFNVVF 222

BnaA09.SIP2_1b LTGGYMNPAAVMGWAYARGEHITKEHLL-VYCLGPVMATLLAVWFYNVVL 219

:** :. : :: . . . .

BnaAnn_random.TIP1_1b IN---------TTHEQLPTTDY---------------------------- 221

BnaAnn_random.TIP1_1a IN---------TTHEQLPTTDY---------------------------- 221

BnaCnn_random.TIP1_1a IN---------TTHEQLPTTDY---------------------------- 221

BnaC07.TIP1_2b INQ--------NGHEQLPTTDY---------------------------- 253

BnaA06.TIP1_2b INQ--------NGHEQLPTTDY---------------------------- 253

BnaC02.TIP1_2a IIE--------NGHEQLPTTDY---------------------------- 253

BnaA02.TIP1_2a IIE--------NGHEQLPTTDY---------------------------- 253

BnaCnn_random.TIP1_3a IDS--------NGHEPLPSSDF---------------------------- 252

BnaA09.TIP1_3a IDS--------NGHEPLPSSDF---------------------------- 252

BnaCnn_random.TIP3_1c IP--TEPPTQHTHHQPLAPEDY---------------------------- 266

BnaA02.TIP3_1a IP--TEPPTQHTHHQPLAPEDY---------------------------- 266

BnaC06.TIP3_1a IP--TEPPT-HPTHQPLAPEDY---------------------------- 265

BnaA07.TIP3_1b IP--TEPPT-HPTHQPLAPEDY---------------------------- 265

BnaC06.TIP3_1b IP--TEPPA-HHTHQPLAPEDY---------------------------- 265

BnaA07.TIP3_1c IP--TEPPA-HHTHQPLAPEDF---------------------------- 265

BnaC08.TIP3_2b IPNVNEPPR-HSTHQPLAPEDY---------------------------- 267

BnaA09.TIP3_2b IPSVNEPPR-HSTHQPLAPEDY---------------------------- 267

BnaC05.TIP3_2a IPNVNEPPR-HSVHQPLAPEDY---------------------------- 267

BnaA06.TIP3_2a IPNVNEPPR-HSVHQPLAPEDY---------------------------- 267

BnaC01_random.TIP2_1d MPSS--------EHVPLASEF----------------------------- 249

BnaA01.TIP2_1a MPSS--------EHVPLASEF----------------------------- 249

BnaC05.TIP2_1b MTS---------EHVPLASEF----------------------------- 248

BnaA05.TIP2_1c MTS---------EHVPLASDF----------------------------- 248

BnaC03.TIP2_1a MSSS--------EHVPLASDF----------------------------- 249

BnaA03.TIP2_1b MSSS--------EHVPLASDF----------------------------- 249

BnaC06.TIP2_1c IAESNQETE--SEHVPLISA------------------------------ 253

BnaC01_random.TIP2_2a IGS--------YAPAPTTESYP---------------------------- 250

BnaA01_random.TIP2_2a IGS--------YAPAPTTESYP---------------------------- 250

BnaC02_random.TIP2_3b IGS--------YQEVETCEIRV---------------------------- 250

BnaA02.TIP2_3a IGS--------YQEVETREIRV---------------------------- 210

BnaA06_random.TIP2_3b IGSP-------YEAVETREIRV---------------------------- 251

BnaC07.TIP2_3a IGSP-------YEAVETREIRV---------------------------- 251

BnaC04.TIP4_1a IDR---------SDVPLADDEQPFLN------------------------ 249

BnaCnn_random.TIP5_1a VVTAAE------DDRGSSTGDATGV------------------------- 258

BnaA06.TIP5_1a VVPAAE------DDRGSSTGDATGV------------------------- 258

BnaCnn_random.PIP2_1b RASGSK------SLGSFRSAANV--------------------------- 287

BnaA09.PIP2_1a RASGSK------SLGSFRSAANV--------------------------- 287

BnaC06.PIP2_1a RASGSK------SLGSFRSAANV--------------------------- 287

BnaC04.PIP2_2/2_3b RASGSK------SLGSFRSAANV--------------------------- 285

BnaA05.PIP2_2/2_3c RASGSK------SLGSFRSAANV--------------------------- 310

BnaC04.PIP2_2/2_3a RASGSK------SLGSFRSAANV--------------------------- 285

BnaA03.PIP2_2/2_3a RASGSK------SLGSFRSAA----------------------------- 283

BnaA03.PIP2_2/2_3b RASGSK------SLGSFRSAANV--------------------------- 285

BnaC03.PIP2_4a RAAGIK------SLGSFRSYA----------------------------- 260

BnaA03.PIP2_4b RAAGIK------SLGSFRSSA----------------------------- 260

BnaA10.PIP2_4c RAAGIK------SLGSFRSSA----------------------------- 260

BnaC09_random.PIP2_4b RAAGIK------SLGSFRSSA----------------------------- 259

BnaA02.PIP2_4a RAAGIK------SLGSFRSSA----------------------------- 259

BnaC08.PIP2_5b RAGAVK------ALGSFRSQSRV--------------------------- 286

BnaA09.PIP2_5b RAGAVK------ALGSFRSQSRV--------------------------- 286

BnaC06.PIP2_5a RAGAVK------ALGSFRSQSHV--------------------------- 286

BnaA07.PIP2_5a RAGAVK------ALGSFRSQSHV--------------------------- 286

BnaC03.PIP2_6a RAGAMK------AYGSVRSQLHELHA------------------------ 288

BnaA03.PIP2_6a RAGAMK------AYGSVRSQLHELHA------------------------ 288

BnaAnn_random.PIP2_7b RAAAVK------ALASFRSSATN--------------------------- 281

BnaC01.PIP2_7a RAAAVK------ALASFRSNATN--------------------------- 281

BnaC03.PIP2_7b RAAAVK------ALASFRSSATN--------------------------- 281

BnaA08.PIP2_7a RAAAVK------ALASFRSSATN--------------------------- 281

BnaC07.PIP2_7c RAAAVK------ALASFRSNATN--------------------------- 254

BnaC07.PIP1_5a RAIPFK------SKT----------------------------------- 287

BnaA03.PIP1_5a RAIPFK------SKT----------------------------------- 287

BnaCnn_random.PIP1_3b RAIPFK------TRT----------------------------------- 286

BnaC05.PIP1_3a RAIPFK------TRT----------------------------------- 286

BnaA10.PIP1_3a RAIPFK------TRS----------------------------------- 286

BnaCnn_random.PIP1_4b RAIPFK------SKN----------------------------------- 286

BnaA09_random.PIP1_4b RAIPFK------SKN----------------------------------- 286

BnaC03.PIP1_4a RAIPFK------SKSKS--------------------------------- 288

BnaA03.PIP1_4a RAIPFK------SKSKS--------------------------------- 288

BnaA09.PIP1_1b RAIPFK------SRN----------------------------------- 286

BnaC08.PIP1_1a RAIPFK------SRN----------------------------------- 286

BnaA04.PIP1_1a RAIPFK------SRS----------------------------------- 225

BnaAnn_random.PIP1_1c RAFPFK------SRS----------------------------------- 206

BnaC04.PIP1_2b RAIPFK------SRS----------------------------------- 286

BnaA05.PIP1_2c RAIPFK------SRS----------------------------------- 286

BnaC03.PIP1_2a RAIPFK------SRS----------------------------------- 286

BnaA03.PIP1_2a RAIPFK------SRS----------------------------------- 286

BnaC04.PIP1_2c RAIPFK------SRS----------------------------------- 286

BnaA04.PIP1_2b RAIPFK------SRS----------------------------------- 286

BnaC07.NIP1_2b YTDK--------PLREITKSGSF-------------------LKSARNGS 296

BnaA03.NIP1_2b YTDK--------PLREITKSGSF-------------------LKSVRNGS 296

BnaC01.NIP1_2a YTDK--------PLREITKSGSF-------------------LKALQNSS 295

BnaA01.NIP1_2a YTDK--------PLREITKSGSF-------------------LKALQNSS 295

BnaC04.NIP2_1b ATQK--------ANSEFSKTGSS-------------------HKRVTDLP 285

BnaA05.NIP2_1b ATQK--------TNPEFSKTGSS-------------------HKRVTDLP 285

BnaC04.NIP2_1a ATQK--------ANSEFSKTGSS-------------------HKRVTDLP 285

BnaA05.NIP2_1a ATQK--------ANSEFSKTGSS-------------------HKRITDLP 285

BnaA04.NIP4_1a FTDK--------PLKELTRNGSF-------------------LRSASPKH 276

BnaC04.NIP4_1a FTDK--------PLKELTRNGSF-------------------LRSASPKH 276

BnaC04.NIP4_1b FTDK--------PLGELTKSSSF-------------------LRKASANN 276

BnaC06_random.NIP4_2a FTDK--------PLRELTKSASF-------------------LRSASPGP 281

BnaA04_random.NIP4_1b FTDK--------PLRELTKSASF-------------------LRSISPKQ 228

BnaC08.NIP3_1c STKK--------SYGEIIRPNCNKIPSRDRQEASQDEI--CVLQVVNQAN 300

BnaA08.NIP3_1c STKK--------SYGEIIRPNCNKISSRDRQEASQDEI--CVLQVVNQAN 273

BnaC05.NIP3_1b STNK--------SYGEIIRPNCNKVSSNDHQEASQDDSDSCVLRVVDPNN 292

BnaC05.NIP3_1a STNK--------SYGEIIRPNCNKVSSNDHQEASQDDSDSCVLRVVDPNN 292

BnaA05.NIP3_1b STNK--------SYGEIIRPNCNKVSSNDHQEASQDDS--CVLRVVDPNN 290

BnaA05.NIP3_1a STNK--------SFGEIIRPNCN-------KEASLEEF--CVLQMVDPNN 290

BnaC03.NIP5_1b LNDS--------ATDPPRQVRSF--------------------RR----- 301

BnaA03.NIP5_1b LNDN--------VTDPPRQVRSF--------------------RR----- 301

BnaC02.NIP5_1a LNDS--------ATDPPRQVRSF--------------------RR----- 301

BnaA02.NIP5_1a LNDS--------ATDPPRQVRSF--------------------RR----- 301

BnaA07.NIP5_1c LNDN--------VSDPPRQVRSF--------------------RR----- 301

BnaC06_random.NIP5_1c LNDN--------VSDPPRQVRSF--------------------RR----- 301

BnaA02_random.NIP6_1c LPE---------EDEEHKEKRSF--------------------RR----- 305

BnaA02.NIP6_1a LPE---------EDEEHKEKRSF--------------------RR----- 305

BnaC06.NIP6_1a LPE---------EDEAPKEKRSF--------------------RR----- 305

BnaA07.NIP6_1b LPE---------EDEAPKEKRSF--------------------RR----- 305

BnaC05.NIP7_1a LKSR--------PSPHSPPVSSLLR------------------------- 272

BnaA05.NIP7_1a LKSR--------PSPHSPPVSSLLR------------------------- 272

BnaA05_random.SIP1_1b PP-----------PTPQQK-----------KQKKA--------------- 239

BnaC05.SIP1_1b PP-----------HKPQQK-----------KQKKA--------------- 239

BnaC01.SIP1_1a PPSPSPSP-----SRPQKKQKKQKKQKKAEKQKKA--------------- 256

BnaA01.SIP1_1a PPSPSP-------PRPQKKQKKQKKPKKAEKQKKA--------------- 254

BnaC09_random.SIP1_2a PP-----------PLPVQK-----------KQKKA--------------- 243

BnaA10.SIP1_2a PP-----------PLPVQK-----------KQKKA--------------- 243

BnaCnn_random.SIP2_1c KPLTEEQQ-----EKPKAKSE----------------------------- 238

BnaA07.SIP2_1a KPLTEEQQ-----EKPKAKSE----------------------------- 238

BnaC04.SIP2_1b RPLTEEE------EKPKAKTD----------------------------- 237

BnaC03.SIP2_1a RPLTEEE------EKPKAKTD----------------------------- 237

BnaA09.SIP2_1b KPLTEEH------EKPKAKSE----------------------------- 234

BnaAnn_random.TIP1_1b -----------------------

BnaAnn_random.TIP1_1a -----------------------

BnaCnn_random.TIP1_1a -----------------------

BnaC07.TIP1_2b -----------------------

BnaA06.TIP1_2b -----------------------

BnaC02.TIP1_2a -----------------------

BnaA02.TIP1_2a -----------------------

BnaCnn_random.TIP1_3a -----------------------

BnaA09.TIP1_3a -----------------------

BnaCnn_random.TIP3_1c -----------------------

BnaA02.TIP3_1a -----------------------

BnaC06.TIP3_1a -----------------------

BnaA07.TIP3_1b -----------------------

BnaC06.TIP3_1b -----------------------

BnaA07.TIP3_1c -----------------------

BnaC08.TIP3_2b -----------------------

BnaA09.TIP3_2b -----------------------

BnaC05.TIP3_2a -----------------------

BnaA06.TIP3_2a -----------------------

BnaC01_random.TIP2_1d -----------------------

BnaA01.TIP2_1a -----------------------

BnaC05.TIP2_1b -----------------------

BnaA05.TIP2_1c -----------------------

BnaC03.TIP2_1a -----------------------

BnaA03.TIP2_1b -----------------------

BnaC06.TIP2_1c -----------------------

BnaC01_random.TIP2_2a -----------------------

BnaA01_random.TIP2_2a -----------------------

BnaC02_random.TIP2_3b -----------------------

BnaA02.TIP2_3a -----------------------

BnaA06_random.TIP2_3b -----------------------

BnaC07.TIP2_3a -----------------------

BnaC04.TIP4_1a -----------------------

BnaCnn_random.TIP5_1a -----------------------

BnaA06.TIP5_1a -----------------------

BnaCnn_random.PIP2_1b -----------------------

BnaA09.PIP2_1a -----------------------

BnaC06.PIP2_1a -----------------------

BnaC04.PIP2_2/2_3b -----------------------

BnaA05.PIP2_2/2_3c -----------------------

BnaC04.PIP2_2/2_3a -----------------------

BnaA03.PIP2_2/2_3a -----------------------

BnaA03.PIP2_2/2_3b -----------------------

BnaC03.PIP2_4a -----------------------

BnaA03.PIP2_4b -----------------------

BnaA10.PIP2_4c -----------------------

BnaC09_random.PIP2_4b -----------------------

BnaA02.PIP2_4a -----------------------

BnaC08.PIP2_5b -----------------------

BnaA09.PIP2_5b -----------------------

BnaC06.PIP2_5a -----------------------

BnaA07.PIP2_5a -----------------------

BnaC03.PIP2_6a -----------------------

BnaA03.PIP2_6a -----------------------

BnaAnn_random.PIP2_7b -----------------------

BnaC01.PIP2_7a -----------------------

BnaC03.PIP2_7b -----------------------

BnaA08.PIP2_7a -----------------------

BnaC07.PIP2_7c -----------------------

BnaC07.PIP1_5a -----------------------

BnaA03.PIP1_5a -----------------------

BnaCnn_random.PIP1_3b -----------------------

BnaC05.PIP1_3a -----------------------

BnaA10.PIP1_3a -----------------------

BnaCnn_random.PIP1_4b -----------------------

BnaA09_random.PIP1_4b -----------------------

BnaC03.PIP1_4a -----------------------

BnaA03.PIP1_4a -----------------------

BnaA09.PIP1_1b -----------------------

BnaC08.PIP1_1a -----------------------

BnaA04.PIP1_1a -----------------------

BnaAnn_random.PIP1_1c -----------------------

BnaC04.PIP1_2b -----------------------

BnaA05.PIP1_2c -----------------------

BnaC03.PIP1_2a -----------------------

BnaA03.PIP1_2a -----------------------

BnaC04.PIP1_2c -----------------------

BnaA04.PIP1_2b -----------------------

BnaC07.NIP1_2b SR--------------------- 298

BnaA03.NIP1_2b SR--------------------- 298

BnaC01.NIP1_2a SR--------------------- 297

BnaA01.NIP1_2a SR--------------------- 297

BnaC04.NIP2_1b L---------------------- 286

BnaA05.NIP2_1b L---------------------- 286

BnaC04.NIP2_1a L---------------------- 286

BnaA05.NIP2_1a L---------------------- 286

BnaA04.NIP4_1a KTSTSKS---------------- 283

BnaC04.NIP4_1a KTSTSKS---------------- 283

BnaC04.NIP4_1b NASSSNS---------------- 283

BnaC06_random.NIP4_2a NR--------------------- 283

BnaA04_random.NIP4_1b -----------------------

BnaC08.NIP3_1c RKEFICSSPTDINDKRNVTCKLP 323

BnaA08.NIP3_1c RKEFICSSPTDINDKRNVTCKLP 296

BnaC05.NIP3_1b RKFFILSSPTDINETCNVTCKLA 315

BnaC05.NIP3_1a RKFFILSSPTDINETCNVTCKLA 315

BnaA05.NIP3_1b RKIFILSSPTDINETCNVTCKLA 313

BnaA05.NIP3_1a RKIFILSSPIDINDTCNITCKLA 313

BnaC03.NIP5_1b -----------------------

BnaA03.NIP5_1b -----------------------

BnaC02.NIP5_1a -----------------------

BnaA02.NIP5_1a -----------------------

BnaA07.NIP5_1c -----------------------

BnaC06_random.NIP5_1c -----------------------

BnaA02_random.NIP6_1c -----------------------

BnaA02.NIP6_1a -----------------------

BnaC06.NIP6_1a -----------------------

BnaA07.NIP6_1b -----------------------

BnaC05.NIP7_1a -----------------------

BnaA05.NIP7_1a -----------------------

BnaA05_random.SIP1_1b -----------------------

BnaC05.SIP1_1b -----------------------

BnaC01.SIP1_1a -----------------------

BnaA01.SIP1_1a -----------------------

BnaC09_random.SIP1_2a -----------------------

BnaA10.SIP1_2a -----------------------

BnaCnn_random.SIP2_1c -----------------------

BnaA07.SIP2_1a -----------------------

BnaC04.SIP2_1b -----------------------

BnaC03.SIP2_1a -----------------------

BnaA09.SIP2_1b -----------------------
